# Serology change-based clinical interpretation of indeterminate serostatus post-hepatitis B virus infection in people living with HIV

**DOI:** 10.1371/journal.pone.0336924

**Published:** 2025-11-20

**Authors:** Ei Kinai, Mio Ishikura, Ryui Miyashita, Tomoko Yamaguchi, Yushi Chikasawa, Akito Ichiki, Ryoko Sekiya, Masato Bingo, Takashi Muramatsu, Mihoko Yotsumoto, Takeshi Hagiwara, Kagehiro Amano

**Affiliations:** 1 Department of Laboratory Medicine, Tokyo Medical University, Tokyo, Japan; 2 Department of Infectious Disease, Tokyo Metropolitan Komagome Hospital, Tokyo, Japan; Centers for Disease Control and Prevention, UNITED STATES OF AMERICA

## Abstract

After hepatitis B virus (HBV) infection in people living with HIV (PLWH), various forms of indeterminate serostatuses, including “isolated anti-HBc (IAHBc)”, are observed, but the current interpretation for their HBV immunity seems to be optimistic. This single-center, retrospective cohort study of 1,461 PLWH included individuals with past natural HBV infection and negative history of HBV vaccination. Further, based on their most recent serology status, the selected individuals were classified into either *1)* seroconversion, *2)* chronic infection, or *3)* indeterminate groups. PLWH of the latter group (with indeterminate serostatus) were defined as neither seroconversion [HBsAg(-)/anti-HBs(+)/anti-HBc(+)] nor chronic infection [HBsAg(+)/anti-HBs(-)/anti-HBc(+)]; chronological sub-serostatus of these individuals and clinical interpretations were determined based on long-term serological changes. Of the 878 PLWH with past-HBV infection and no vaccination, seroconversion was documented in 640 (73%), chronic infection in 60 (7%), and 178 (20%) were considered the indeterminate group. Based on a review of 13-year serologic tests (9 test repeats), patients of the indeterminate group were classified as either “isolated anti-HBc (IAHBc)” (n = 118, 66%), “anti-HBs alone” (n = 35, 20%), or “lost anti-HBs/anti-HBc” (n = 25, 14.0%). None showed “resolved infection” pattern. IAHBc was significantly associated with weak HBV immunity, such as viral rebound or non-seroconversion [odds ratio (OR) 2.181, 95% confidence interval (95%CI) (1.064–4.469)], while anti-HBs alone was not [OR: 0.143, 95%CI: 0.041–0.492]. Clinical interpretations of lost anti-HBs/anti-HBc were identical to those of IAHBc. In PLWH, IAHBc and lost anti-HBs/anti-HBc do not indicate resolved but weak/unstable immunity against HBV, whereas anti-HBs alone infers robust immunity.

## Introduction

After natural hepatitis B virus (HBV) infection in people living with HIV (PLWH), an indeterminate serostatus, such as “isolated anti-HBc (IAHBc)”, representing negative hepatitis B surface antibody (anti-HBs) and positive hepatitis B core antibody (anti-HBc) coupled with negative hepatitis B surface antigen (HBsAg), is more often observed in such individuals than in non-PLWH [1–3]. While IAHBc is clinically interpreted as either *1)* resolved infection, *2)* occult infection, *3)* false positive, or *4)* a mutant hepatitis B surface antigen (HBsAg) strain [4,5], the prevailing consensus considers IAHBc mainly reflects resolved infection, based on stable serologic values associated with absence of viral rebound and re-infection [1,2,6]. Hence, the major guidelines assume that the majority of IAHBc cases reflect robust HBV immunity where “anti-HBs levels had waned” years or decades after stable seroconversion [4,7,8]. However, some studies reported strong association between IAHBc and occult hepatitis B infection (OBI) [9–12] and hepatocellular carcinoma [13]. To our knowledge, there are no long-term IAHBc follow-up studies with repeat serology that have demonstrated a decline/disappearance of anti-HBs over years or decades.

“Anti-HBs alone” with positive anti-HBs and negative anti-HBc is interpreted as “immune from receipt of prior vaccination (if documented complete vaccine series), and “negative anti-HBs/anti-HBc” with negative anti-HBs and negative anti-HBc is interpreted as “never infected (if no documentation of vaccine series completion)” [4]. However, several studies have reported that both are not rare after HBV infection in unvaccinated patients [1–3], but to our knowledge, the clinical interpretation of these two patterns has never been discussed extensively.

There is a growing need for reassessment of HBV immunity in PLWH. Evidence suggests that the rate of new HBV infection among men-who-have-sex-with-men (MSM) is high [14], and that the efficacy of HBV vaccines in PLWH is low [15–17]. Even in those PLWH who had been vaccinated during infancy, HBV infection can occur during adulthood [18]. Furthermore, HBV reactivation has been observed in individuals with past infection under various settings, such as administration of anti-hepatitis C therapy and immunosuppressive therapy [19,20]. Today, PLWH are protected against HBV infection or reactivation because the majority currently use antiretroviral therapy that includes the combination of tenofovir (TFV) [tenofovir disoproxil fumarate (TDF) or tenofovir alafenamide (TAF)] plus [lamivudine (3TC) or emitricitabine (FTC)], both of which have potent anti-HBV activity. However, the recent use of two-drug antiretroviral therapies, such as those that exclude TFV (dolutegravir/3TC) [21] or completely omit anti-HBV agents (dolutegravir/rilpivirine and cabotegravir/ rilpivirine) [22,23], raises concern about the potential for new HBV infection [23,24] or reactivation [25]. Although current guidelines recommend the use of TFV plus FTC/3TC in chronic or occult HBV infection, there are no recommendations for PLWH with indeterminate serostatus [7].

The major aims of the present study were first to clarify whether IAHBc is the result of waning and disappearance of anti-HBs, and second, to provide clinical interpretation to “anti-HBs alone” and “lost anti-HBs/anti-HBc” as well as IAHBc, based on long-term follow-up of serostatus following HBV infection.

## Methods

### Study participants and study protocol

**Fig 1:**
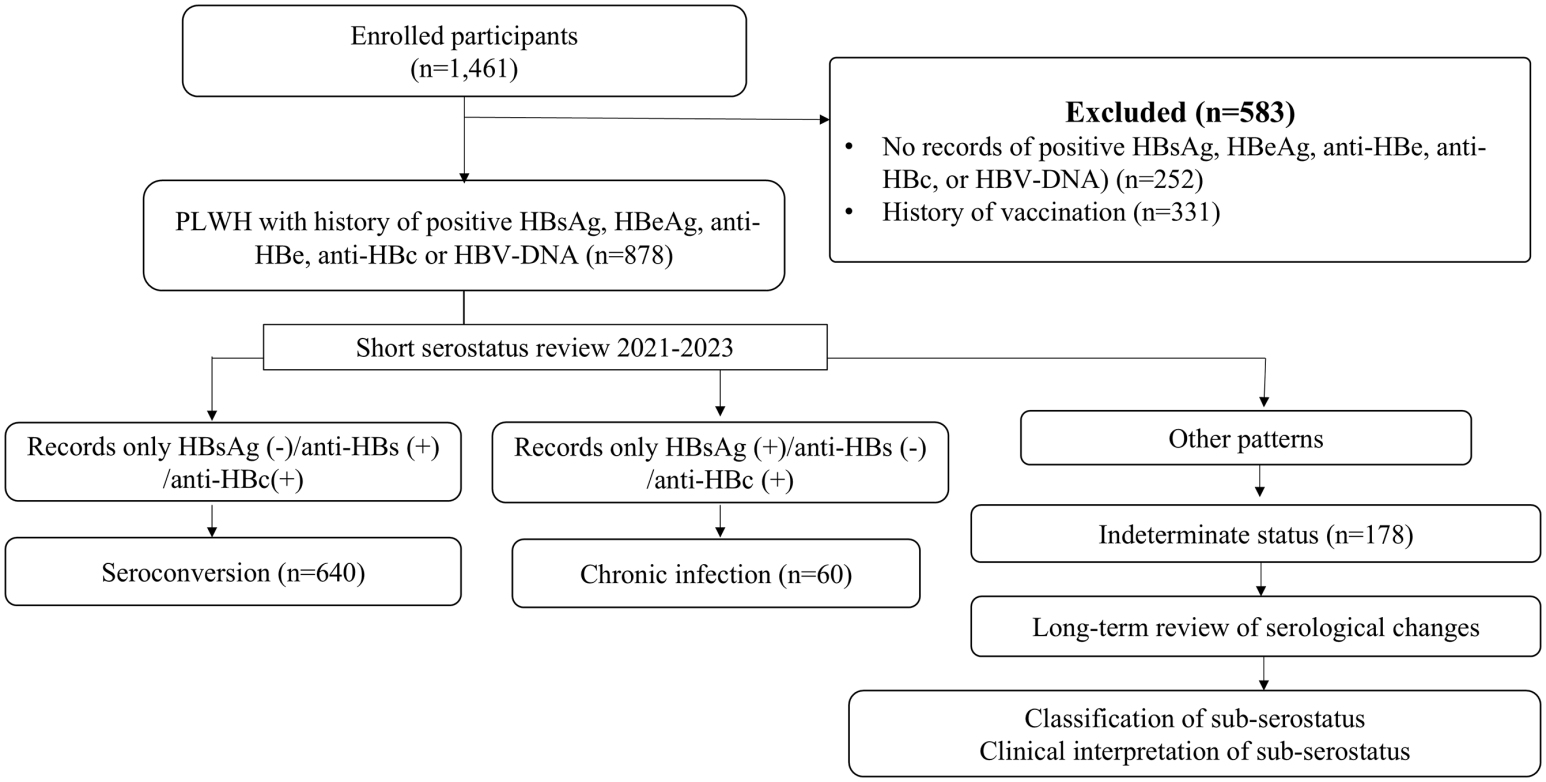
Flow diagram of subject enrolment and classification. Serostatus screening conducted in 878 people living with HIV (PLWH) and past hepatitis B virus (HBV) infection allowed their classification into either seroconversion, chronic infection, or indeterminate pattern. Serial changes in serological tests occurring during the long observation period were reviewed and analyzed in PLWH with indeterminate serostatus.

Fig 1 shows the study flow and subject classification. First, in this single-center and retrospective cohort study, we included only PLWH with past natural HBV infection and negative history for HBV vaccination based on comprehensive review of the medical record of each individual. Past natural HBV infection was defined as any record of a positive test for HBsAg, HBeAg, anti-HBe, anti-HBc or HBV-DNA. Second, based on the most recent serology status during the period between January 2021 and September 2023, the selected 878 individuals were classified into either *1)* seroconversion (n = 640), *2)* chronic infection (n = 60), or *3)* indeterminate (n = 178) groups. The seroconversion group represented PLWH who were HBsAg-negative, anti-HBs-positive, and anti-HBc-positive. The chronic infection group represented PLWH who were HBsAg-positive, anti-HBs-negative, and anti-HBc-positive. All other PLWH were categorized as indeterminate serostatus, and they included those with inconsistent test results of several tests or a single test results inconsistent with seroconversion and chronic infection.

The study was approved by the Ethics Review Committee of Tokyo Medical University (T2023-0184) and conducted according to the principles of the Declaration of Helsinki. All data were accessed in fully anonymized style on January 29, 2024, and any information that identifies individuals was not accessible. According to the Japanese ethical guidelines for medical and biological research involving human subjects, the requirement for consent was waived due to the retrospective nature of the study.

### Serological tests and other information

For serological tests of HBsAg, anti-HBs, HBeAg, anti-HBe, and total anti-HBc, Lumipulse Prest II (FUJIREBIO, Co., Tokyo, Japan) was used from April 2003 to July 2013. This was replaced in August 2013 with HBsAg QT Abbott (CLIA), Ausab Abbott (CLIA), HBeAg Abbott (CLIA), HBeAb Abbott (CLIA), and HBc Abbott (CLIA) (Abbott Laboratories, Chicago, IL) with the ARCHITECT analyzer i2000SR (Abbott Laboratories). HBV-DNA was measured using COBAS TaqMan HBV ver 2.0 from 2012 to 2019, followed by COBAS 6800/8800 system HBV since 2019 (Roche Diagnostics, Indianapolis, IN). Hepatitis B core-related antigen (HBcrAg) was measured by Lumipulse Prest iTACT HBcrAg (FUJIREBIO, Tokyo). HIV-RNA was measured using Amplicorv ver 1.5 from 2002 to 2007, and by TaqMan ver. 1 between 2007–2012, and TaqMan ver. 2 since 2012 (Roche Diagnostics). The S1 Table lists the quantification ranges and decision thresholds of the above assays. CD4 and HIV viral load were obtained at the latest date when HBV serology data were available during the screening period (2021–2023), and history of anti-retroviral regimen were obtained by reviewing the entire medical records.

### Definitions of sub-serostatuses

The sub-serostatus of the group of PLWH with an indeterminate serostatus was determined by reviewing the titers of HBsAg, HBsAb, HBeAg, HBeAb, HBcAb, HBV-DNA and HBcrAg measured between October 2003 and September 2023. Generally, serostatus, such as IAHBc, is defined by each single set of anti-HBV antibodies; however, this definition could not be applied in our study to determine the long-term serostatus since the serostatus changed during the period, with unstable titers of anti-HBV antibody in PLWH with indeterminate serostatus. For this reason, we applied new definitions for the long-term sub-serostatus, which were based on chronological changes in serology (Table 1). Thus, PLWH with one or more tests negative for anti-HBs and positive for anti-HBc were defined as “IAHBc”, irrespective of the results of other serologic tests. Similarly, “anti-HBs alone” was defined as any history of serostatus with positive anti-HBs and negative anti-HBc, whereas “lost anti-HBs/anti-HBc” represented any history of disappearance of both anti-HBs and anti-HBc.

**Table 1:** Definition of sub-serostatus classification.

Classification of sub-serostatus	Definition
Isolated anti-HBc	At least one recorded instance of anti-HBs (–) with anti-HBc (+).
Anti-HBs alone	At least one recorded instance of anti-HBs (+), with subsequent disappearance of anti-HBc following prior positivity.
Lost anti-HBs/anti-HBc	At least one recorded instance of anti-HBs (–), with disappearance of anti-HBc following prior positivity.

### Clinical interpretations of serological changes

Based on the assessment of serological changes during the study period, we assigned clinical interpretation to PLWH with indeterminate serostatus (Table 2). First, the majority of PLWH without primary infection data (positive HBsAg or HBV-DNA) were classified as “unknown” since other possible interpretations could be assigned, while definite clinical interpretations were assigned to those PLWH with primary infection data. Second, we classified the HBV immunity robustness into “weak”, “robust”, and “resolved infection”, based on the chronological stability of anti-HBs or the appearance of HBsAg/HBV-DNA (Table 2). Weak immunity was defined as unstable anti-HBs after primary infection or positive HBsAg/HBV-DNA, whereas robust immunity was defined as persistently positive anti-HBs after initial seroconversion. Third, we further assigned detailed interpretations to address highly variable serologic patterns. For example, when PLWH had no record of positive anti-HBs after primary infection, they were labeled as “non-seroconversion”. When PLWH showed repeated appearance/disappearance of anti-HBs, they were labeled as “weak/unstable seroconversion”.

**Table 2:** Simplified classification of HBV serologic profiles and inferred immune status.

Category	Primary Infection	Definition
**Weak HBV immunity**	Yes	Unstable or incomplete serologic response after infection.
Chronic infection	Yes	Persistent HBsAg despite negative anti-HBc.
Virologic rebound	Yes/ No	Re-elevation of HBV-DNA after prior clearance.
Non-seroconversion	Yes	No anti-HBs detected post-infection.
Unstable seroconversion	Yes	Fluctuating anti-HBs presence.
Early loss of anti-HBs	Yes	Anti-HBs lost within 3 years of seroconversion.
Late seroconversion	Yes	Anti-HBs appears >3 years post-infection.
**Robust HBV immunity**	Yes	Stable anti-HBs following seroconversion.
Sustained seroconversion	Yes	Persistent high anti-HBs, ± anti-HBc.
Post-exposure reaction	Yes	Abrupt anti-HBs rise (≥3× or >100 IU/mL).
**Resolved infection**	Yes	Gradual decline or loss of anti-HBs after seroconversion.
**Unknown**	No	No infection record; interpretation inferred from serologic trends.
Possible weak response	No	No or unstable anti-HBs throughout.
Possible late conversion	No	Anti-HBs rise after years of negativity.
Possible resolution	No	Decline in modest anti-HBs levels.
Possible post-exposure	No	Abrupt anti-HBs rise without prior seroconversion.

Furthermore, for the “unknown” group, the adopted “possible” clinical interpretations were based on definitions. PLWH with primary infection data of the “Definite resolution” group represented those who showed a gradual fall in anti-HBs level after seroconversion, while those without primary infection data of the “possible resolution” group showed gradual fall of anti-HBs.

### Review of chronological changes in serostatus

To quantify the chronological changes in anti-HBs and anti-HBc in PLWH classified with the indeterminate serostatus, we counted the number of changes in serostatus during the long-term follow-up. For example, for a PLWH who showed a swing from a baseline serostatus of negative anti-HBs and positive anti-HBc, to a positive anti-HBs and positive anti-HBc serostatus, and again to negative anti-HBs and positive anti-HBc, the count of serostatus changes was two. We also evaluated the association of the serostatus count with sub-serostatus and clinical interpretations.

### Statistical analysis

The demographic characteristics of the participating patients were compared according to the serostatus (seroconversion, chronic infection and indeterminate serostatus). For the sub-serostatus of the indeterminate group, we compared the demographic characteristics and chronological observatory information (including the duration of the observation period, frequency of serological tests, duration of test intervals, and duration of treatment with anti-HBV agents). For comparison among the three serostatuses and three sub-serostatuses, one-way analysis of variance (ANOVA) was used for comparison of continuous variables with normal distribution while Kruskal-Wallis test was used for those with skewed distribution. Either the chi-square or Fisher’s exact test was applied for categorical variables. Adjusted logistic multivariate models were generated to evaluate the association between sub-serostatus and weak HBV immunity. All statistical analyses were performed using the Statistical Package for Social Sciences (ver. 23.0, SPSS, Chicago).

## Results

### Clinical characteristics of study participants

Screening of 878 cases with past HBV infection without history of vaccination showed seroconversion (positive anti-HBs and anti-HBc) in 640 (73%) patients, chronic infection (positive HBsAg, negative anti-HBs and positive anti-HBc) in 60 (7%), and indeterminate patterns in 178 (20%). Comparison of the three serostatus groups demonstrated that PLWH of the indeterminate group were older, while those of the seroconversion group had lower CD4 cell count. Anti-HBV agents were more often used by the chronic infection and indeterminate groups than the seroconversion group (Table 3).

**Table 3:** Characteristics of participants at screening.

	Total (n = 878)	Seroconversion (n = 640)	Chronic Infection (n = 60)	Indeterminate status (n = 178)	p-value
Age, mean (SD)	47.5 (10.8)	48.7 (9.8)	47 (12.6)	51.4 (10.2)	0.003
Females, n (%)	11 (1.3)	6 (0.9)	1 (1.7)	4 (2.2)	0.364
MSM, n (%)	842 (95.9)	612 (95.6)	57 (95.0)	173 (97.2)	0.892
HCV-Ab positive, n (%)	54 (6.2)	33 (5.2)	2 (3.3)	19 (10.7)	0.016
CD4 at screening, mean (SD)	605 (41.5)	591 (229)	640 (287.0)	598 (336.0)	0.028
CD4 < 200/μL at screening, n (%)	28 (3.2)	20 (3.1)	2 (3.3)	6 (3.4)	0.984
CD 4/8 ratio, mean (SD)	0.99 (0.48)	1.00 (0.47)	0.94 (0.35)	1.00 (0.41)	0.701
CD 4/8 < 1.00, n (%)	498 (56.7)	360 (56.3)	37 (61.7)	101 (56.7)	0.720
HIV RNA < 50 at screening, n (%)	819 (93.3)	597 (93.3)	54 (90.0)	168 (94.4)	0.503
On HIV treatment, n (%)	872 (99.3)	635 (99.2)	60 (100.0)	178 (100.0)	0.883
History of anti-HBV agents, n (%)					
TDF	544 (62.0)	372 (58.1)	36 (60.0)	136 (76.4)	0.000
TAF	687 (78.2)	480 (75.0)	58 (96.7)	149 (83.7)	0.000
TDF or TAF	749 (85.3)	529 (82.7)	58 (96.7)	162 (91.0)	0.001
3TC	430 (49.0)	334 (52.2)	11 (18.3)	85 (47.8)	0.000
FTC	749 (85.3)	529 (82.7)	58 (96.7)	162 (91.0)	0.001
3TC or FTC	863 (99.4)	635 (99.2)	60 (100.0)	178 (100.0)	0.338
ETV	3 (0.3)	0 (0.0)	1 (1.7)	2 (1.1)	0.014

TDF: tenofovir disoproxil fumarate, TAF: tenofovir alafenamide, TDF: tenofovir disoproxil fumarate, 3TC: lamivudine, ETV: entecavir.

### Clinical characteristics of indeterminate group by sub-serostatus

Analysis of the serological tests showed a median [interquartile range (IQR)] observation period of 13.0 (9.3–15.5) years, during which various serologic tests were performed (average: 9.0, range: 7–13) times per person, with inter-serologic test interval of 15.5 (range: 9.5–20.5) years. Of the 178 PLWH of the indeterminate group, 118 (66%) were classified as IAHBc, 35 (20%) as anti-HBs alone, and 25 (14%) were lost anti-HBs/anti-HBc. The type of sub-serostatus was independent of age, sex, observation period, test numbers, and test intervals (Table 4).

**Table 4:** Characteristics of patients with indeterminate serostatus against hepatitis B virus.

	All indeterminate status	isolated anti-HBc	anti-HBs alone	Lost anti-HBs/anti-HBc	P-value
Number of cases (% of total cases)	178 (100)	118 (66.3)	35 (19.7)	25 (14.0)	
Age at screening, median (IQR)	50 (44.0-57.0)	50 (44.0-57.0)	52 (47.4-58.0)	47.0 (39.0-55.0)	0.524
Females, n (%)	4 (2.2)	2 (1.7)	1 (2.9)	1 (4.0)	0.751
MSM, n (%)	173 (97.2)	115 (97.5)	34 (97.1)	24 (96.0)	0.868
Observation period (years), median (IQR)	13.0 (9.3-15.0)	13.0 (9.0-15.0)	13.0 (11.0-16.0)	13.0 (11.0-14.0)	0.788
Number of serologic test repeats, median (IQR)	9.0 (7.0-13.0)	9.0 (7.0-13.0)	9.0 (6.0-11.5)	10.0 (9.0-15.0)	0.352
Interval between serologic tests (years), median (IQR)	1.3 (0.8-1.7)	1.2 (0.9-1.7)	1.4 (1.0-1.8)	1.1 (0.8-1.4)	0.205
HCV-Ab positive, n (%)	19 (10.7)	12 (10.2)	3 (8.6)	4 (16.0)	0.626
CD4/μL at screening, median (IQR)	588 (449-785)	592 (447-794)	663 (440-895)	569 (519-942)	0.360
CD4 < 200/μL at screening, n (%)	6 (3.4)	5 (4.2)	1 (2.9)	0 (0.0)	0.556
CD4/8 < 1 at screening, n (%)	101 (56.7)	68 (16.0)	16 (45.7)	17 (68.0)	0.216
HIV RNA < 50 cp/mL at screening, n (%)	168 (94.4)	110 (93.2)	34 (97.1)	24 (96.0)	0.629
On HIV treatment, n (%)	178 (100)	118 (100)	35 (100)	25 (100)	NS
History of anti-HBV agents, n (%)					
TDF	136 (76.4)	88 (74.6)	28 (80.0)	20 (80.0)	0.723
TAF	149 (83.7)	99 (83.9)	27 (77.1)	23 (92.0)	0.306
TDF or TAF	162 (91.0)	108 (91.5)	31 (88.6)	23 (92.0)	0.851
3TC	85 (47.8)	58 (49.2)	16 (45.7)	11 (44.0)	0.864
FTC	162 (91.0)	108 (91.5)	31 (88.6)	23 (92.0)	0.851
3TC or FTC	178 (100.0)	118 (100.0)	35 (100.0)	25 (100.0)	NS
ETV	2 (1.1)	1 (0.8)	0 (0.0)	1 (4.0)	0.310
Duration of anti-HBV agents (years), median (IQR)					
TDF	4.5 (1.7-6.4)	3.9 (0.0-7.8)	5.4 (1.1-6.3)	4.5 (1.7-6.4)	0.984
TAF	5.2 (3.4-6.3)	5.7 (2.3-6.4)	6.2 (1.8-6.3)	5.1 (3.4-6.3)	0.943
3TC	0.0 (0.0-6.3)	0.0 (0.0-8.3)	0.0 (0.0-8.3)	0.0 (0.0-6.3)	0.896
FTC	10.2 (4.8-12.0)	10.0 (4.0-13.7)	10.7 (4.6-12.3)	10.2 (4.8-12.0)	0.971
ETV	0.0 (0.0-0.0)	0.0 (0.0-0.0)	0.0 (0.0-0.0)	0.0 (0.0-0.0)	0.317

Chi-square test was used for nominal variables. Statistical tests by one-way analysis of variance (ANOVA) for continuous variables with normal distribution and Kruskal-Wallis test for those with skewed distribution. IQR: interquartile range, MSM: men sex with men, TDF tenofovir disoproxil fumarate, TAF; tenofovir alafemamide, 3TC; lamivudine, FTC: emtricitabine, ETV: entecavir, NA: not available, NS: not significant.

### Clinical interpretation of indeterminate serostatus

Weak HBV immunity was observed in 47/118 (39.8%) of IAHBc and in 11/25 (44.0%) of lost anti-HBs/anti-HBc, whereas it was observed only in 3/35 (8.6%) of anti-HBs alone. Specifically, chronic infection, viral rebound, non-seroconversion and weak/unstable seroconversion were observed in IAHBc and lost anti-HBs/anti-HBc, but not among anti-HBs alone (Table 5). In contrast, all PLWH with sustained seroconversion were positive for anti-HBs alone, but not IAHBc or lost anti-HBs/anti-HBc. Surprisingly, despite the current consensus interpretation, no PLWH showed a resolved infection pattern in any of the serostatus groups (Table 5).

**Table 5:** Clinical interpretations of indeterminate serostatus based on long-term serological changes.

Clinical Interpretation	All indeterminate status (n = 178)	Isolated anti-HBc (n = 118)	Anti-HBs alone (n = 35)	Lost anti-HBs/anti-HBc (n = 25)
**Weak HBV immunity, Total, n (%)**	**61 (34.3)**	**47 (39.8)**	**3 (8.6)**	**11 (44.0)**
Chronic infection, n (%)	2 (1.1)	0 (0.0)	0 (0.0)	2 (8.0)
Viral rebound	5 (2.8)	4 (3.4)	0 (0.0)	1 (4.0)
Non-seroconversion, n (%)	12 (6.7)	11 (9.3)	0 (0.0)	1 (4.0)
Weak/unstable seroconversion, n (%)	15 (8.4)	12 (10.2)	0 (0.0)	3 (12.5)
Early loss of anti-HBs, n (%)	9 (5.1)	7 (5.0)	0 (0.0)	2 (8.0)
Late seroconversion, n (%)	18 (10.1)	13 (11.0)	3 (8.6)	2 (8.0)
**Robust HBV immunity, Total, n (%)**	**32 (18.0)**	**1 (0.8)**	**28 (80.0)**	**3 (12.0)**
Sustained seroconversion, n (%)	23 (12.9)	0 (0.0)	23 (65.7)	0 (0.0)
Post-exposure anti-HBs elevation, n (%)	9 (5.1)	1 (0.8)	5 (14.3)	3 (12.0)
**Resolved infection, n (%)**	**0 (0.0)**	**0 (0.0)**	**0 (0.0)**	**0 (0.0)**
**Unknown, n (%)**	**85 (47.8)**	**70 (59.3)**	**4 (11.4)**	**11 (44.0)**
Possible non/weak seroconversion	55 (30.9)	48 (40.7)	1 (2.9)	6 (24.0)
Possible late seroconversion	3 (1.7)	3 (2.5)	0 (0.0)	0 (0.0)
Possible post-exposure anti-HBs elevation	5 (2.8)	5 (4.2)	0 (0.0)	0 (0.0)
Possible resolved infection	22 (12.4)	14 (11.9)	3 (8.6)	5 (20.0)
**Logistic multivariate analysis for weak HBV immunity**				
Crude		2.175 (1.077-4.392)	0.137 (0.040-0.470)	1.619 (0.686-3.821)
Adjusted model 1		2.166 (1.072-4.379)	0.138 (0.040-0.474)	1.613 (0.498-5.270)
Adjusted model 2		2.170 (1.058-4.452)	0.143 (0.042-0.494)	1.587 (0.650-3.877)

Model 1 was adjusted by the history of tenofovir-based treatment. Model 2 was adjusted by the history of tenofovir-based treatment, age, CD4 cell counts, and HIV suppression (<50 cp/mL) at the screening point.

Logistic multivariate analysis confirmed the association between sub-serostatus and HBV immunity. Even in adjusted models by the history of TFV-based treatment, age, CD4 cell counts, and HIV suppression at screening point, IAHBc was a significant predictor of weak HBV immunity [odds ratio (OR) 2.170, 95% confidence interval (95%CI) (1.058–4.452)], whereas anti-HBs alone inferred robust HBV immunity [OR: 0.143, 95%CI: 0.042–0.494] (Table 5).

### Chronological changes in serostatus

Tentative seroconversion was observed in 114/178 (64%) of the entire indeterminate group, 73/118 (61.9%) of IAHBc, 33/35 (94.3%) of anti-HBs alone, and 8/25 (32.0%) of lost anti-HBs/anti-HBc. Only 39/178 (26%) showed stable serostatus (no change in serology) throughout the observation period, while the count of serostatus changes was > 4 in 14 (8%) PLWH (Table 6).

**Table 6:** Count of serological changes by indeterminate sub-serostatus and clinical interpretations.

	No change	Once	2-3 times	≥4 times
**SUB-SEROSTATUS**				
All indeterminate (n = 178)	39 (22%)	63 (35%)	60 (34%)	14 (8%)
Isolated anti-HBc (n = 118)	33 (28%)	36 (31%)	40 (34%)	9 (2%)
Anti-HBs alone (n = 35)	5 (14%)	19 (54%)	9 (25%)	2 (6%)
Lost anti-HBs/anti-HBc (n = 25)	1 (4%)	13 (32%)	9 (44%)	2 (8%)
**CLINICAL INTERPRETATIONS**				
Weak HBV immunity (n = 61)	7 (11%)	21 (34%)	24 (39%)	9 (15%)
Chronic infection, (n = 2)	0 (0%)	0 (0%)	1 (50%)	1 (50%)
Viral rebound (n = 5)	0 (0%)	1 (20%)	3 (60%)	1 (20%)
Non-seroconversion (n = 12)	7 (58%)	5 (42%)	0 (0%)	0 (0%)
Weak/unstable seroconversion (n = 15)	0 (0%)	5 (33%)	7 (47%)	3 (20%)
Early loss of anti-HBs (n = 9)	0 (0%)	2 (22%)	4 (45%)	3 (33%)
Late seroconversion (n = 18)	0 (0%)	8 (44%)	9 (50%)	1 (6%)
Robust HBV immunity (n = 32)	3 (9%)	19 (59%)	7 (22%)	3 (9%)
Sustained seroconversion (n = 23)	3 (13%)	14 (61%)	5 (22%)	1 (4%)
Post-exposure anti-HBs elevation (n = 9)	0 (0%)	5 (56%)	2 (22%)	2 (22%)
Resolution (n = 0)	0	0	0	0
Unknown (n = 85)	29 (34%)	23 (27%)	29 (34%)	4 (5%)

The count of serostatus changes did not correlate with clinical interpretation. For example, in subjects who showed no seroconversion pattern, anti-HBs remained negative without any change during the observation period, indicating weak immunity. On the other hand, some cases of late seroconversion showed frequent changes in serology until stable seroconversion, indicating robust immunity (Table 6).

### Case presentations of indeterminate serostatus group

**Fig 2:**
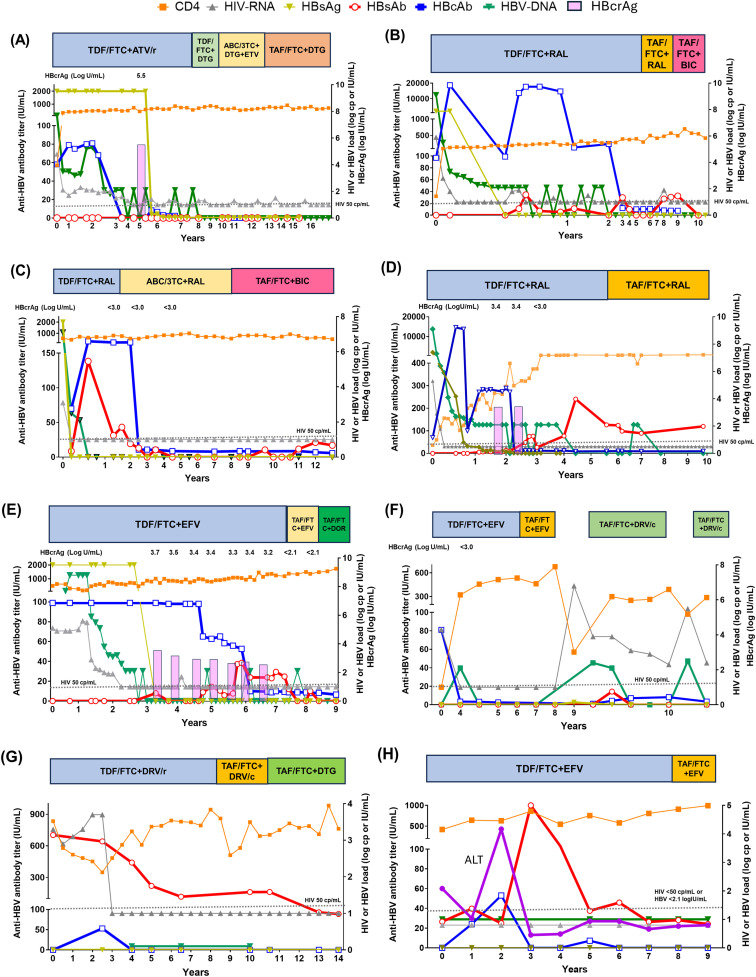
Serologic changes in 8 representative cases of sub-serostatus and clinical interpretation groups. **(A)** Lost anti-HBs/anti-HBc case interpreted as “non-seroconversion”. Anti-HBs was never detected since primary infection. Sub-serostatus was initially IAHBc during primary infection, and then changed to “Lost anti-HBs/anti-HBc” following disappearance of anti-HBc. **(B)** An IAHBc case interpreted as “unstable seroconversion”. Note the frequent disappearance of Anti-HBs titer after the primary infection. **(C)** An IAHBc case interpreted as “early loss of anti-HBs”. Despite the high titer of anti-HBs during primary infection, the level decreased rapidly within 3 years. Although anti-HBs was negative during tenofovir-sparing antiretroviral therapy, its level increased again after tenofovir-based regimen. **(D)** An IAHBc case interpreted as “late seroconversion”. Note the appearance and rise of anti-HBs to >100 IU/mL about 3 years after the primary infection. **(E)** An IAHBc case interpreted as a complex of “late seroconversion, early loss of anti-HBs, and viral rebound”. Note the appearance of Anti-HBs at 5 years after primary infection, and its disappearance after 2 years with occasional viral rebound throughout the observation period. **(F)** An IAHBc case interpreted as “viral rebound” by treatment interruption. Note that treatment interruption resulted in a rebound in both HIV and HBV, suggesting that IAHBc inferred poor immunity against HBV. **(G)** A case positive for anti-HBs alone interpreted as “possible resolution”. Due to lack of primary infection data, it was not clear how many years had passed since the primary infection. Note the persistently negative Anti-HBc after the first 4 years of appearance. The alternative diagnosis of late seroconversion could not be ruled out. **(H)** A case positive for anti-HBs alone interpreted as “post-exposure protective reaction”. Note the abrupt increase in Anti-HBs from 24.9 to 1000 IU/mL in the third year of observation, followed by its decrease to 37.5 IU/mL by the fifth year. Although the parallel rise in aminotransferase and anti-HBc suggested exposure to HBV, HBV-DNA was negative throughout the observation period. TDF: tenofovir disoproxil fumarate, FTC: emitricitabine, ATV: atazanavir, TDF: tenofovir disoproxil fumarate, DTG: dolutegravir, ABC: abacavir, 3TC: lamivudine, ETV: entecavir, TAF: tenofovir alafenamide, RAL: raltegravir.

Fig 2 shows serial changes in various serological tests (CD4, HIV-RNA, HBsAg, HBsAb, HBV-DNA, HBcrAg, and anti-HBV Ab titer), during the indicated long observation periods in representative cases of various serostatuses who received various therapeutic regimens and considered to have different clinical interpretations. The case shown in Fig 2A, who was labeled as Lost anti-HBs/anti-HBc serostatus, never showed positive anti-HBs after primary infection and exhibited waning anti-HBc. The case was clinically interpreted as “non-seroconversion”. On the other hand, the case depicted in Fig 2F who was labeled as IAHBc showed repeated viral rebound due to interruption of anti-HBV agents.

## Discussion

The key findings of the present study are: *1)* IAHBc indicates weak immunity, while “anti-HBs alone” strongly infers robust immunity, 2) none of the IAHBc group showed a “resolved pattern”, with fall and disappearance of anti-HBs years or decades after the initial seroconversion, and *3)* unstable serostatus in the indeterminate group, highlighting the importance of repeated serologic tests to establish the correct diagnosis. These findings emphasize the fragility of HBV immunity in PLWH.

Although numerous studies have attempted clinical interpretation of IAHBc, several major guidelines and studies interpret IAHBc as resolved infection. Evidence supporting the concept of resolution includes: *1)* a stable serostatus in long-term follow-up [2], *2)* comparable HBV-specific T cell and memory B cell responses between the IAHBc and seroconversion groups [26], and *3)* the relatively favorable vaccine response to HBV [17,27,28]. However, one study from Taiwan with extensive serological testing [3] reported the finding of unstable antibodies in IAHBc, which was also seen in our study. Interestingly, there are no studies that have examined T cell or memory B cell responses in PLWH, who are known to have significantly impaired T cell function [26]. In the present study, some PLWH with late seroconversion showed stable seroconversion, suggesting that IAHBc is a mixture of robust and weak/unstable immunity. Based on such heterogeneous HBV immunity in IAHBc, some groups offered “diagnostic HBV-vaccine booster” to identify PLWH with robust immunity in IAHBc [5]. However, it is noteworthy that: *1)* none of the IAHBc cases showed a resolved pattern with gradual fall in anti-HBs after seroconversion; and *2)* post-exposure protection was observed in only 1/118 case (1%) of IAHBc, whereas it was observed in 5/35 cases (14%) of anti-HBs alone. These findings suggest that IAHBc in immunodeficient individuals is rather more indicative of latent or chronic infection than resolved infection. Indeed, such concern is reflected in the practice guidelines for prevention of HBV reactivation in candidates for immunosuppressive therapy [29]. Furthermore, despite the lack of significant association with weak HBV immunity in logistic analysis, the clinical interpretation of lost anti-HBs/anti-HBc was strictly identical to that of IAHBc, suggesting that lost anti-HBs/anti-HBc also indicates weak HBV immunity.

The prevalence and significance of HBV serostatus can be affected by HBV epidemiology and genotypes [5]. For example, in Japan, HBV is highly endemic in MSM and PLWH [14] most likely due to delayed vaccine policy. Extrapolation of our findings may be limited to the economically developed countries, where HBV prevalence is low due to early induction of universal vaccination programs. In contrast, HBV remains endemic in most African, several Asian, and Eastern Mediterranean countries, largely due to the delayed implementation of universal vaccination programs [30,31]. Furthermore, although genotype C is traditionally predominant among elderly Japanese individuals [32], genotype A is more common in younger Japanese populations and PLWH [33]. In our study, among 85 PLWH with documented HBV genotypes—most of whom had chronic infections—47 (55%) were genotype A, 13 (15%) were genotype C, and 20 (24%) were unclassified (data not shown). Genotype A is distributed globally, particularly in Europe and South-East Africa [34,35]. Accordingly, the applicability of our findings may extend to select Asian, Eastern Mediterranean, and South-East African countries.

Various serological anomalies in HBV infection result from a complex interplay between viral persistence mechanisms [36] and host immune dysregulation, such as exhaustion and functional impairment of HBV-specific T cells and B cells [37,38]. In addition, one proposed explanation for IAHBc involves the consumption of anti-HBs through immune complex formation with their cognate antigens [39]. Since HBcAg is the most recognized immunogenic viral component, anti-HBc may persist long after acute infection [4,40]; however, its concentration also reflects latent HBV-DNA levels and is associated with hepatic disease severity [9,12]. Recent *ex vivo* findings by Wang et al. [29] offer insight to HBV-specific immune dynamics. Their data demonstrated suppression of intrahepatic covalently closed circular DNA (cccDNA) and HBV-DNA even in IAHBc cases, alongside preserved HBV-specific T-cell responses in individuals who had undergone seroconversion.

In non-PLWH, HBsAg can seldom be suppressed by nucleos(t)ide analogues (NAs) [41] because of host immune dysregulation. Meanwhile, in HIV/HBV coinfection, HBsAg can disappear by NAs [42] possibly due to the immune reconstitution by HIV treatments. In IAHBc, insufficient immune reconstitution hinders the production of sufficient anti-HBs to exceed elevated HBsAg, indicative of a substantial cccDNA reservoir. Conversely, in cases with anti-HBs alone, relatively preserved T/B-cell function may suppress HBsAg, but sometimes fail to mount a detectable response because of minimal HBcAg stimulation, which are likely suppressed by anti-HBV therapy (S1 Fig).

Recently, there has been a shift from conventional triple-agent antiretroviral therapy to dual therapy. However, switching to an anti-HBV agent-sparing therapy raises the concern of new HBV infection or, more importantly, reactivation [25,43]. Our study also found viral rebound in an IAHBc case due to interruption of anti-HBV agents (Fig 2F). Hence, in IAHBc and cases with lost anti-HBs/anti-HBc, careful interpretation is needed in switching ART to anti-HBV agents-sparing therapy. On the other hand, the risk of viral rebound after switching to tenofovir-sparing regimen in IAHBc and lost anti-HBs/anti-HBc is not clear. Although HBV infection can be prevented with 3TC monotherapy, resistant viruses could emerge in infected cases, whilst no new HBV infections or resistant HBV viruses have been reported in patients receiving TDF [44]. Tenofovir-containing regimens have better clinical outcomes than 3TC-containing regimens in liver-related mortality rate as well as virologic suppression [45,46]. Moreover, the combination of TAF and FTC, which increases intracellular concentration, resulted in a higher rate of HBsAg disappearance than TDF/FTC, but low seroconversion rates were noted in patients of both treatment groups (9% vs. 7%) [42]. These findings indicate that HBsAg suppression in PLWH is strongly dependent on anti-HBV therapy. For individuals with undetectable or low anti-HBs titers (*e.g.,* < 100 IU/mL), we recommend the following approach: *1)* Repeated serological testing: Concurrent assessment of HBsAg, anti-HBs, anti-HBc, and HBV-DNA should be performed at least two to three times to ensure diagnostic accuracy and capture potential fluctuations in serological markers. *2)* Continuation of HBV-active ART: Maintenance of ART regimens containing agents with anti-HBV activity is advised, both to suppress HBV replication and to mitigate the risk of HBV reactivation.

Apart from the above strengths of our study, the study design has several limitations that need to be discussed. First, although IAHBc in immunocompetent individuals is expected to have different entities from those in immunodeficient people [17,26,47,48], the present study did not compare these groups of subjects. Second, we did not conduct long-term serologic reviews in cases with HBsAg(-)/anti-HBs(+)/anti-HBc(+) or HBsAg(+)/anti-HBs(-)/anti-HBc(+). Based on the highly changeable and different anomalies in HBV serostatus, it is possible that a percentage of such cases might have been missed despite transient indeterminate patterns. Third, one can argue that the 13-year observation period was not long enough to identify the resolved pattern. Our findings suggest that some of possible non-seroconversion can include resolved pattern, with waning of anti-HBs for longer than 20 years. Finally, although assay platforms for HBV markers changed over the 13-year duration of this retrospective study, we consider the impact on serostatus trends to be minimal. First, the lower limits of detection remained largely consistent across assay generations, and second, results obtained using Lumipulse (2003–2012) aligned closely with those from Architect, which was employed in a recent study covering 2013–2023 [45].

In conclusion, PLWH often show indeterminate HBV serostatus after HBV infection, with high instability chronologically, raising risks of misdiagnosis. IAHBc and lost anti-HBs/anti-HBc in PLWH indicate weak and unstable immunity rather than resolved infection, whereas anti-HBs alone infers robust immunity.

Key points: In people living with HIV, indeterminate serostatus of hepatitis B virus (HBV) can change widely, raising risk of misdiagnosis. Isolated anti-HBc and lost anti-HBs/anti-HBc do not indicate resolved but weak/unstable immunity against HBV, whereas anti-HBs alone infers robust immunity.

## Supporting information

S1 Fig Schematic overview of proposed immunological mechanisms underlying HBV serostatus, based on the interplay between HBV-specific T/B-cell function and intrahepatic cccDNA volume. Each row represents a distinct serological profile, with symbolic circles indicating relative levels of immune function (blue) and cccDNA reservoir size (red). The presence (+) or absence (–) of anti-HBs and anti-HBc antibodies is also shown.
(TIF)

S1 Table The list of assay reagents, quantification ranges and threshold values for positivity decision (or limit of detection) of HBsAg, anti-HBs, total anti-HBc, and HBV-DNA. (DOCX)

Supporting information 1 Anonymized dataset of characteristics of all of the study participants. (PDF)

Supporting information 2 Anonymized dataset of chronological serology change in PLWH with indeterminate serostatus. (PDF)
