# Supplementary figures and images for: Serology change-based clinical interpretation of indeterminate serostatus post-hepatitis B virus infection in people living with HIV
Source: PLoS One. 2025 Nov 20;20(11):e0336924. doi: 10.1371/journal.pone.0336924 (PMC12633944; doi:10.1371/journal.pone.0336924)

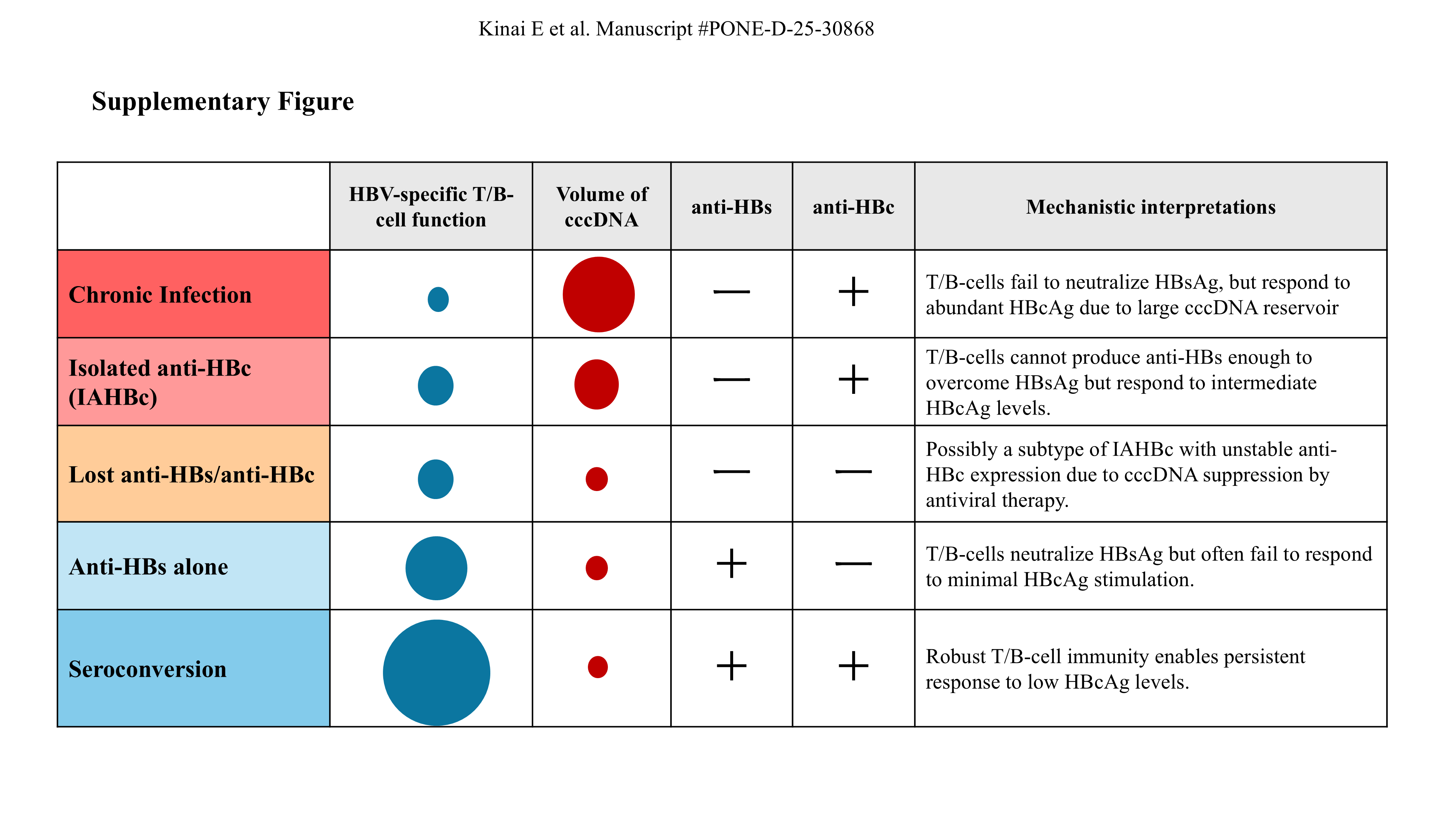

Supplement: S1 Fig — The presence (+) or absence (–) of anti-HBs and anti-HBc antibodies is also shown. (TIF) [file pone.0336924.s001.TIF]
