# Supplementary material for: Serology change-based clinical interpretation of indeterminate serostatus post-hepatitis B virus infection in people living with HIV
Source: PLoS One. 2025 Nov 20;20(11):e0336924. doi: 10.1371/journal.pone.0336924 (PMC12633944; doi:10.1371/journal.pone.0336924)
Supplement: S1 Table — (DOCX) [file pone.0336924.s002.docx]

Supplementary Table 1: Assay reagents, quantification ranges and threshold values for positivity decision (or limit of detection) of HBV markers.

| HBV markers | from 2003 to 2013 | from 2013 to 2022 | from 2022 to 2023 |
| --- | --- | --- | --- |
| HBsAg | Lumipulse HBsAg (CLEIA)  Cut-off index 0.1-2000.0 IU/mL  Cut-off index 1.0 IU/mL | HBsAg QT Abbott (CLIA)  0.05-250.00 IU/mL  0.05 IU/mL | HBsAg QT Abbott (CLIA)  0.02-250.00 IU/mL  0.05 IU/mL |
| anti-HBs | Lumipulse HBsAb (CLEIA)  0.1-1000.0 mIU/mL  5.0 mIU/mL | Ausab Abbott (CLIA)  2.5-1000.0 mIU/mL  10 mIU/mL | Ausab Abbott (CLIA)  2.0-1000.0 mIU/mL  10 mIU/mL |
| anti-HBc | Lumipulse HBcAb (CLEIA)  Cut-off index 0.1-300.0  Cut-off index 1.0 | HBcAb Abbott (CLIA)  0-9999 S/CO  1.0 S/CO | HBcAb Abbott(CLIA)  0-9999 S/CO  1.0 S/CO |
|  | from 2012-2019 | from Jul. 2019 to Dec.2019 | from 2019 to 2023 |
| HBV-DNA | COBAS TaqMan HBV ver 2.0  2.1-9.0 log cp/mL  Limit of detection 2.1 log cp/mL | COBAS 6800/8800 system HBV  1.8-9.8 log cp/mL  Limit of detection 1.8 log cp/mL | COBAS 6800/8800 system HBV  1.0-9.0 log IU/mL  Limit of detection 1.0 log IU/mL |
