# Supplementary material for: Serology change-based clinical interpretation of indeterminate serostatus post-hepatitis B virus infection in people living with HIV
Source: PLoS One. 2025 Nov 20;20(11):e0336924. doi: 10.1371/journal.pone.0336924 (PMC12633944; doi:10.1371/journal.pone.0336924)
Supplement: Supporting information 1 — (PDF) [file pone.0336924.s003.pdf]

Sex	Age	HCV-Ab	HCVRNA	status	既感染あ	vaccine	HBsAbscr	HBcAbscr	seroconve	chronic in	Indetermir	observation	TAF(M)	TDF(M)	3TC(M)	TAF(1/0)	TDF(1/0)	3TC(1/0)	FTC	CD4%	CD8%	CD4/8	CD4_count	CD8_count	HIV_RNA
1	74	(−)		P	1	0	1	1	1	0						0	0	1	0	28.6	44.2	0.65	838	1296	0
1	52	(−)		P	1	0	1	1	1	0	0					0	0	1	0	34.7	20.1	1.73	505	292	0
1	61	(−)		P	1	0	1	1	1	0	0					1	1	0	1	24.6	35.9	0.69	547	796	20
1	55	(−)		P	1	0	1	1	1	0	0					1	1	1	1	30.7	21.7	1.42	839	593	26
1	42	(−)		P	1	0	1	1	1	0	0					1	0	1	1	29.3	29.8	0.98	598	609	0
1	53	(−)		P	1	0	1	1	1	0	0					0	1	0	1	46.0	31.9	1.44	286	198	0
1	43	(−)		P	1	0	1	1	1	0						0	0	0	0	23.5	44.0	0.53	590	1107	12000
1	39	(−)		P	1	0	1	1	1	0						0	0	0	0	29.0	15.6	1.86	559	301	230
1	37	(−)		C	1	0	0	1	0	1						0	0	0	0	9.3	66.0	0.14	155	1100	62000
1	34	(−)		P	1	0	1	1	1	0						0	0	0	0	20.3	57.9	0.35	577	1650	11000
1	55	(−)	ｹﾝｼｭﾂｾｽ	P	1	0	1	1	1	0	0					0	0	1	0	36.7	29.9	1.23	764	622	0
1	39	(−)		P	1	0	1	1	1	0	0					0	0	1	0	32.3	30.8	1.05	559	533	20
1	40	(−)		P	1	0	1	1	1	0	0					1	0	1	1	34.5	43.2	0.80	564	705	0
1	38	(−)		P	1	0	1	1	1	0	0					1	1	1	1	29.0	27.8	1.05	498	476	0
1	62	(−)		P	1	0	1	1	1	0	0					1	1	1	1	52.8	14.8	3.57	1166	327	0
1	54	(−)		P	1	0	1	1	1	0	0					1	1	0	1	26.4	36.7	0.72	589	819	0
1	58	(−)		P	1	0	1	1	1	0	0					1	0	1	1	31.8	24.1	1.32	755	574	0
1	50	(−)		P	1	0	1	1	1	0	0					1	0	1	1	17.8	38.4	0.46	260	559	20
1	52	(−)		C	1	0	0	1	0	1	0					1	1	1	1	30.9	42.6	0.72	806	1113	23
1	60	(−)		P	1	0	1	1	1	0	0					1	1	0	1	31.5	31.7	1.00	470	472	0
1	53	(−)		P	1	0	1	1	1	0	0					0	0	1	0	35.7	31.8	1.12	416	371	0
1	40	(−)	ｹﾝｼｭﾂｾｽ	P	1	0	1	1	1	0	0					1	1	0	1	41.2	28.8	1.43	693	484	0
1	51	(−)		P	1	0	1	1	1	0	0					0	1	1	1	27.9	52.8	0.53	533	1012	20
1	81	(−)		P	1	0	1	1	1	0	0					1	0	1	1	17.1	23.7	0.72	290	400	0
1	56	(−)		P	1	0	1	1	1	0	0					1	1	1	1	33.9	33.1	1.02	514	502	0
1	55	(−)		P	1	0	1	1	1	0	0					1	1	1	1	26.2	23.7	1.11	562	508	20
1	51	4.8	ｹﾝｼｭﾂｾｽ	P	1	0	1	1	1	0	0					1	0	1	1	28.3	21.1	1.34	517	386	28
1	52	(−)		P	1	0	1	1	1	0	0					1	1	1	1	29.3	27.3	1.07	829	773	20
1	51	(−)	ｹﾝｼｭﾂｾｽ	P	1	0	1	1	1	0	0					1	0	1	1	19.4	52.9	0.37	592	1613	0
1	56	(−)		P	1	0	1	1	1	0	0					1	1	0	1	26.3	29.7	0.88	499	565	20
1	59	(−)		P	1	0	1	1	1	0	0					1	1	0	1	27.1	50.3	0.54	549	1021	0
1	59	(−)		C	1	0	0	1	0	1	0					1	1	0	1	32.5	40.8	0.80	509	638	20
1	55	(−)		P	1	0	1	1	1	0	0					1	1	0	1	36.4	30.2	1.21	836	694	0
1	51	(−)		P	1	0	1	1	1	0	0					1	1	1	1	25.4	27.6	0.92	443	482	0
1	57	(−)		P	1	0	1	1	1	0	0					1	1	1	1	29.2	25.3	1.16	208	180	0
1	53	(−)		P	1	0	1	1	1	0	0					1	1	0	1	37.0	37.6	0.98	612	622	0
1	52	(−)		P	1	0	1	1	1	0	0					1	1	0	1	23.7	28.4	0.83	430	516	0
1	58	(−)		P	1	0	1	1	1	0	0					0	0	1	0	41.2	33.0	1.25	1083	868	0
1	54	(−)		P	1	0	1	1	1	0	0					1	1	1	1	34.7	25.6	1.35	429	317	0
1	67	58.1	ｹﾝｼｭﾂｾｽ	P	1	0	1	1	1	0	0					1	1	1	1	27.1	18.5	1.47	431	294	0
1	48	(−)		P	1	0	1	1	1	0	0					1	1	0	1	35.6	30.0	1.19	441	372	0
1	54	(−)		P	1	0	1	1	1	0	0					1	1	0	1	39.2	22.4	1.75	547	313	20
1	78	(−)		C	1	0	0	1	0	1	0					1	1	1	1	37.3	26.6	1.40	539	384	20
1	57	22.8	ｹﾝｼｭﾂｾｽ	P	1	0	1	1	1	0	0					1	1	0	1	28.9	38.7	0.74	586	786	0
1	71	(−)		P	1	0	1	1	1	0	0					1	1	1	1	15.7	26.8	0.59	320	547	0
1	50	(−)		P	1	0	1	1	1	0	0					1	0	1	1	27.5	34.8	0.79	359	454	0
1	50	(−)		P	1	0	1	1	1	0	0					0	0	1	0	33.5	39.3	0.85	1058	1241	0
1	65	(−)		P	1	0	1	1	1	0	0					1	1	1	1	29.9	30.5	0.98	482	493	0
1	51	(−)		P	1	0	1	1	1	0	0					1	1	0	1	49.0	21.0	2.33	851	365	0
1	57	(−)		P	1	0	1	1	1	0	0					1	0	1	1	23.2	29.8	0.78	577	740	0
1	56	(−)		P	1	0	1	1	1	0	0					1	1	0	1	19.0	24.7	0.77	541	705	0
2	49	(−)		P	1	0	1	1	1	0	0					1	1	1	1	42.7	26.2	1.63	794	487	0
1	67	(−)		P	1	0	1	1	1	0	0					1	1	1	1	24.5	38.8	0.63	245	387	0
1	57	(−)		P	1	0	1	1	1	0	0					1	0	1	1	38.0	20.1	1.89	983	520	0
1	45	(−)		P	1	0	1	1	1	0	0					1	1	1	1	27.8	40.0	0.70	711	1020	37
1	48	(−)		P	1	0	1	1	1	0	0					1	1	0	1	31.9	31.5	1.01	764	755	0
1	69	(−)		P	1	0	1	1	1	0	0					0	1	1	1	13.5	30.6	0.44	272	617	0
1	73	(−)		P	1	0	1	1	1	0	0					1	1	0	1	27.8	33.8	0.82	322	391	0
1	53	(−)		P	1	0	1	1	1	0	0					0	0	1	0	50.5	14.0	3.61	1513	419	45
1	49	(−)		P	1	0	1	1	1	0	0					1	1	0	1	37.5	38.0	0.99	542	550	0
1	55	(−)		P	1	0	1	1	1	0	0					0	0	1	0	32.9	35.5	0.92	613	663	0

Sex	Age	HCV-Ab	HCVRNA	status	既感染あ	vaccine	HBsAbscr	HBcAbscr	seroconve	chronic in	Indetermir	observation	TAF(M)	TDF(M)	3TC(M)	TAF(1/0)	TDF(1/0)	3TC(1/0)	FTC	CD4%	CD8%	CD4/8	CD4_count	CD8_count	HIV_RNA
1	48			P	1	0	1	1	1	0	0					1	1	0	1	29.5	21.6	1.37	459	336	70
1	65	(−)		P	1	0	1	1	1	0	0					1	1	1	1	31.2	24.9	1.26	694	553	0
1	53	(−)		P	1	0	1	1	1	0	0					1	1	1	1	30.0	38.4	0.78	1265	1618	20
1	54	(−)		P	1	0	1	1	1	0	0					0	1	1	1	35.8	33.7	1.06	665	627	0
1	54	(−)		P	1	0	1	1	1	0	0					1	1	1	1	38.8	19.4	2.00	642	322	20
1	59	(−)	ｹﾝｼｭﾂｾｽﾞ	P	1	0	1	1	1	0	0					0	0	1	0	22.8	33.5	0.68	299	440	0
1	49	(−)		P	1	0	1	1	1	0	0					0	1	1	1	39.9	39.0	1.02	555	542	0
1	48	(−)		P	1	0	1	1	1	0	0					1	1	1	1	36.2	35.5	1.02	580	568	0
1	45	(−)		P	1	0	1	1	1	0	0					1	1	1	1	42.8	34.3	1.25	1040	832	0
1	53	(−)		P	1	0	1	1	1	0	0					1	0	1	1	43.4	30.9	1.40	459	327	0
1	56	(−)		P	1	0	1	1	1	0	0					1	1	1	1	18.1	59.0	0.31	415	1354	20
1	58	(−)		P	1	0	1	1	1	0	0					1	1	1	1	44.7	23.8	1.88	733	390	0
1	59	(−)		P	1	0	1	1	1	0	0					1	1	1	1	30.3	34.0	0.89	814	913	0
1	48	(−)		P	1	0	1	1	1	0	0					1	1	0	1	25.6	44.7	0.57	388	678	20
1	49	(−)		P	1	0	1	1	1	0	0					0	0	1	0	34.0	43.8	0.78	724	934	0
1	48	(−)		P	1	0	1	1	1	0	0					0	0	1	0	33.6	18.5	1.81	175	96	25
1	51	(−)		P	1	0	1	1	1	0	0					1	0	0	1	24.6	21.5	1.14	404	354	24
1	48	(−)		P	1	0	1	1	1	0	0					1	1	1	1	24.3	45.8	0.53	490	922	0
1	51	(−)		P	1	0	1	1	1	0	0					0	1	1	1	32.0	20.3	1.57	578	367	0
1	52	(−)	ｹﾝｼｭﾂｾｽﾞ	P	1	0	1	1	1	0	0					1	1	1	1	22.0	26.4	0.84	710	850	0
1	68	(−)		P	1	0	1	1	1	0	0					1	1	1	1	20.8	38.0	0.55	275	502	0
1	48	(−)		P	1	0	1	1	1	0	0					0	0	1	0	32.1	27.0	1.19	486	409	0
1	76	6.3	ｹﾝｼｭﾂｾｽﾞ	P	1	0	1	1	1	0	0					1	1	1	1	20.6	57.7	0.36	507	1417	0
1	60	(−)		P	1	0	1	1	1	0	0					1	1	0	1	30.1	26.5	1.13	648	571	0
1	52	(−)		P	1	0	1	1	1	0	0					0	1	1	1	37.3	23.9	1.56	546	350	0
1	77	(−)		P	1	0	1	1	1	0	0					0	1	1	1	22.6	46.0	0.49	376	765	0
1	60	(−)		P	1	0	1	1	1	0	0					0	0	1	0	36.3	30.6	1.19	560	472	0
1	88	(−)		P	1	0	1	1	1	0	0					1	1	1	1	14.3	32.9	0.43	236	544	0
1	60	34.4	ｹﾝｼｭﾂｾｽﾞ	P	1	0	1	1	1	0	0					0	1	1	1	23.8	40.3	0.59	413	701	0
1	55	(−)		P	1	0	1	1	1	0	0					1	1	1	1	30.2	37.5	0.80	458	569	20
1	47	1.3	ｹﾝｼｭﾂｾｽﾞ	P	1	0	1	1	1	0	0					1	1	0	1	40.2	19.3	2.08	694	333	20
1	59	8.8	ｹﾝｼｭﾂｾｽﾞ	P	1	0	1	1	1	0	0					1	1	1	1	32.8	32.1	1.02	825	807	20
1	50	(−)		P	1	0	1	1	1	0	0					1	1	0	1	29.9	38.0	0.79	479	609	20
1	50	(−)		P	1	0	1	1	1	0	0					1	1	1	1	4.3	46.2	0.09	41	443	8200
1	60	(−)	ｹﾝｼｭﾂｾｽﾞ	P	1	0	1	1	1	0	0					0	0	1	0	36.3	27.4	1.33	471	355	0
1	48	(−)	ｹﾝｼｭﾂｾｽﾞ	P	1	0	1	1	1	0	0					1	1	0	1	24.4	45.9	0.53	256	483	0
1	50	(−)	ｹﾝｼｭﾂｾｽﾞ	P	1	0	1	1	1	0	0					0	1	1	1	25.0	39.7	0.63	478	758	0
1	65	(−)		P	1	0	1	1	1	0	0					1	0	1	1	38.0	30.5	1.25	493	396	0
1	62	(−)		C	1	0	0	1	0	1	0					1	1	0	1	29.4	30.2	0.97	810	834	20
1	51	(−)		P	1	0	1	1	1	0	0					1	0	1	1	28.8	30.3	0.95	478	503	0
1	59	(−)		P	1	0	1	1	1	0	0					0	0	1	0	20.0	46.6	0.43	562	1306	0
1	37	(−)	ｹﾝｼｭﾂｾｽﾞ	P	1	0	1	1	1	0	0					1	0	0	1	28.3	39.9	0.71	780	1099	0
1	59	(−)		P	1	0	1	1	1	0	0					1	0	1	1	38.2	15.7	2.44	629	258	0
1	49	(−)		P	1	0	1	1	1	0	0					1	1	1	1	35.8	37.3	0.96	673	702	20
1	55	(−)		P	1	0	1	1	1	0	0					0	0	1	0	29.3	51.7	0.57	451	795	0
1	49	(−)	ｹﾝｼｭﾂｾｽﾞ	P	1	0	1	1	1	0	0					1	1	0	1	38.7	30.7	1.26	423	336	31
1	47	(−)	ｹﾝｼｭﾂｾｽﾞ	P	1	0	1	1	1	0	0					1	1	1	1	32.4	29.9	1.09	622	573	37
1	64	(−)		P	1	0	1	1	1	0	0					1	0	1	1	20.0	29.5	0.68	357	528	20
1	44	(−)		P	1	0	1	1	1	0	0					1	0	1	1	25.6	33.7	0.76	507	666	0
1	53	(−)		P	1	0	1	1	1	0	0					1	1	1	1	28.2	38.6	0.73	498	681	0
1	66	(−)		P	1	0	1	1	1	0	0					0	1	1	1	33.2	35.5	0.93	609	652	20
1	58	(−)		P	1	0	1	1	1	0	0					0	0	1	0	12.8	55.3	0.23	505	2181	0
1	79	(−)		P	1	0	1	1	1	0	0					0	0	1	0	38.9	29.8	1.31	510	390	0
1	49	(−)		P	1	0	1	1	1	0	0					1	1	0	1	41.6	20.1	2.07	1241	598	0
1	46	(−)		P	1	0	1	1	1	0	0					0	1	1	1	30.0	32.0	0.94	446	476	0
1	62	(−)		P	1	0	1	1	1	0	0					1	1	0	1	30.1	45.2	0.67	430	644	0
1	46	(−)	ｹﾝｼｭﾂｾｽﾞ	P	1	0	1	1	1	0	0					1	1	1	1	25.1	42.2	0.60	351	589	0
1	60	(−)		P	1	0	1	1	1	0	0					1	1	0	1	29.6	34.0	0.87	392	450	20
1	71	(−)		P	1	0	1	1	1	0	0					1	1	1	1	23.3	26.6	0.88	350	400	0
1	61	(−)		P	1	0	1	1	1	0	0					1	0	0	1	35.0	42.3	0.83	913	1104	39
1	72	3.5	ｹﾝｼｭﾂｾｽﾞ	C	1	0	0	1	0	1	0					1	1	1	1	24.6	12.3	2.00	231	116	20

Sex	Age	HCV-Ab	HCVRNA	status	既感染あ	vaccine	HBsAbscr	HBcAbscr	seroconve	chronic in	Indetermi	observation	TAF(M)	TDF(M)	3TC(M)	TAF(1/0)	TDF(1/0)	3TC(1/0)	FTC	CD4%	CD8%	CD4/8	CD4_count	CD8_count	HIV_RNA
1	42	(−)		P	1	0	1	1	1	0	0					1	0	0	1	35.6	26.4	1.35	694	515	0
1	48	(−)		C	1	0	0	1	0	1	0					1	1	0	1	36.1	24.1	1.50	661	440	0
1	56	(−)		P	1	0	1	1	1	0	0					0	1	1	1	21.1	48.3	0.44	506	1157	0
1	43	(−)		P	1	0	1	1	1	0	0					1	1	0	1	36.0	38.1	0.94	524	555	0
1	46	(−)	ケンシュツセズ	C	1	0	0	1	0	1	0					1	1	0	1	33.3	35.5	0.94	452	483	0
1	55	(−)		P	1	0	1	1	1	0	0					0	1	1	1	26.4	20.8	1.27	577	454	20
1	51	(−)		P	1	0	1	1	1	0	0					1	1	1	1	30.0	40.3	0.74	749	1007	22
1	54	(−)		P	1	0	1	1	1	0	0					1	1	1	1	30.4	40.9	0.74	380	511	20
1	56	(−)		P	1	0	1	1	1	0	0					1	0	1	1	17.4	27.2	0.64	348	543	20
1	50	(−)		P	1	0	1	1	1	0	0					1	1	1	1	30.2	25.6	1.18	635	537	0
1	49	(−)		C	1	0	0	1	0	1	0					1	0	0	1	32.5	39.8	0.82	1279	1565	77
1	55	(−)		P	1	0	1	1	1	0	0					1	0	0	1	36.2	28.5	1.27	487	383	0
1	58	(−)		P	1	0	1	1	1	0	0					1	1	0	1	34.8	27.0	1.29	747	579	20
1	49	(−)		P	1	0	1	1	1	0	0					0	1	1	1	34.1	23.7	1.44	601	419	20
1	49	(−)		C	1	0	0	1	0	1	0					1	1	1	1	27.5	41.1	0.67	634	947	54
1	60	(−)		P	1	0	1	1	1	0	0					0	1	1	1	30.7	26.8	1.15	813	709	0
1	44	(−)		P	1	0	1	1	1	0	0					1	0	0	1	34.3	35.5	0.96	551	571	0
1	49	(−)		P	1	0	1	1	1	0	0					1	0	1	1	35.9	42.9	0.84	705	843	0
1	53	(−)		P	1	0	1	1	1	0	0					0	1	1	1	24.5	12.4	1.97	808	410	96
1	63	(−)		P	1	0	1	1	1	0	0					1	1	0	1	21.0	32.7	0.64	410	638	140
1	64	(−)		P	1	0	1	1	1	0	0					1	0	1	1	33.5	28.3	1.19	518	437	0
1	52	(−)		P	1	0	1	1	1	0	0					1	1	0	1	23.4	19.6	1.19	552	464	76
1	50	(−)		P	1	0	1	1	1	0	0					1	1	0	1	34.9	25.1	1.39	662	475	20
1	61	(−)		P	1	0	1	1	1	0	0					0	0	1	0	22.9	42.8	0.54	507	947	20
1	53	(−)		C	1	0	0	1	0	1	0					1	1	0	1	39.7	24.2	1.64	591	360	0
1	71	(−)		C	1	0	0	1	0	1	0					1	0	0	1	24.4	37.5	0.65	322	496	20
1	47	(−)		P	1	0	1	1	1	0	0					1	1	0	1	34.7	21.7	1.60	510	319	0
1	57	(−)	0.5>	P	1	0	1	1	1	0	0					0	1	1	1	29.4	30.6	0.96	706	736	110
1	58	(−)		P	1	0	1	1	1	0	0					0	0	1	0	28.9	18.2	1.59	350	220	0
1	51	(−)		P	1	0	1	1	1	0	0					0	1	1	1	37.0	37.0	1.00	369	369	0
1	47	(−)		P	1	0	1	1	1	0	0					1	1	1	1	30.4	36.8	0.83	526	636	0
1	74	(−)		P	1	0	1	1	1	0	0					1	1	1	1	29.5	21.6	1.37	613	448	0
1	65	(−)		P	1	0	1	1	1	0	0					0	0	1	0	31.0	19.4	1.59	552	346	0
1	82	(−)	ケンシュツセズ	C	1	0	0	1	0	1	0					1	1	1	1	28.1	26.5	1.06	333	314	0
1	58	(−)	ケンシュツセズ	P	1	0	1	1	1	0	0					0	0	1	0	44.2	27.3	1.62	1043	645	0
1	47	(−)		P	1	0	1	1	1	0	0					0	1	1	1	26.5	23.3	1.14	410	360	0
1	44	4.5	ケンシュツセズ	P	1	0	1	1	1	0	0					0	0	1	0	22.1	34.9	0.63	483	760	20
1	42	(−)		P	1	0	1	1	1	0	0					1	1	0	1	27.1	38.6	0.70	479	682	0
1	51	(−)		P	1	0	1	1	1	0	0					1	0	1	1	32.0	23.4	1.37	647	472	0
1	44	(−)	ケンシュツセズ	P	1	0	1	1	1	0	0					1	1	1	1	43.7	30.0	1.46	645	442	0
1	40	(−)		C	1	0	0	1	0	1	0					1	1	0	1	39.7	46.9	0.85	869	1026	20
1	50	(−)		P	1	0	1	1	1	0	0					1	1	1	1	28.1	32.1	0.88	820	937	0
1	48	(−)		P	1	0	1	1	1	0	0					1	1	1	1	27.3	34.2	0.80	614	768	22
1	47	(−)		P	1	0	1	1	1	0	0					1	0	1	1	37.0	23.5	1.58	894	567	0
1	51	(−)		P	1	0	1	1	1	0	0					1	1	1	1	27.9	39.2	0.71	516	726	0
1	48	(−)	ケンシュツセズ	P	1	0	1	1	1	0	0					0	1	1	1	33.8	24.0	1.41	749	530	0
1	42	(−)		P	1	0	1	1	1	0	0					0	1	1	1	29.5	34.3	0.86	454	528	0
1	48	(−)		C	1	0	0	1	0	1	0					1	1	0	1	34.9	41.7	0.84	719	859	0
1	45	(−)		P	1	0	1	1	1	0	0					0	1	1	1	30.7	23.3	1.32	431	327	0
1	48	(−)		P	1	0	1	1	1	0	0					1	1	1	1	41.0	17.6	2.33	450	193	0
1	55	(−)	ケンシュツセズ	P	1	0	1	1	1	0	0					0	1	1	1	41.5	36.5	1.14	698	614	0
1	56	(−)		P	1	0	1	1	1	0	0					1	1	0	1	28.2	26.2	1.08	697	648	20
1	52	(−)		P	1	0	1	1	1	0	0					1	0	0	1	34.8	13.4	2.60	738	284	20
1	54	(−)		P	1	0	1	1	1	0	0					1	1	1	1	34.0	28.0	1.22	601	494	0
1	45	(−)		P	1	0	1	1	1	0	0					1	1	0	1	37.6	39.3	0.96	887	926	0
1	54	(−)		P	1	0	1	1	1	0	0					1	0	1	1	29.2	41.4	0.71	612	867	0
1	53	(−)	5>	P	1	0	1	1	1	0	0					1	1	0	1	22.2	28.5	0.78	381	490	0
1	39	(−)		P	1	0	1	1	1	0	0					1	1	0	1	34.2	45.2	0.76	780	1032	0
1	50	(−)		P	1	0	1	1	1	0	0					0	0	1	0	27.8	43.7	0.64	466	733	0
1	50	(−)		P	1	0	1	1	1	0	0					0	0	1	0	32.3	28.6	1.13	701	621	0
1	50	(−)		P	1	0	1	1	1	0	0					0	0	1	0	33.7	22.5	1.50	674	451	0

Sex	Age	HCV-Ab	HCVRNA	status	既感染あ	vaccine	HBsAbscr	HBcAbscr	seroconve	chronic in	Indetermi	observation	TAF(M)	TDF(M)	3TC(M)	TAF(1/0)	TDF(1/0)	3TC(1/0)	FTC	CD4%	CD8%	CD4/8	CD4_count	CD8_count	HIV_RNA
1	56	(−)	ｹﾝｼｭﾂｾｽﾞ	P	1	0	1	1	1	0	0					0	1	1	1	35.6	33.7	1.06	752	712	0
1	50	49.6	ｹﾝｼｭﾂｾｽﾞ	P	1	0	1	1	1	0	0					1	1	1	1	32.7	18.2	1.80	433	241	37
1	54	(−)		P	1	0	1	1	1	0	0					0	1	1	1	24.8	48.2	0.51	714	1390	0
1	53	(−)		P	1	0	1	1	1	0	0					1	1	1	1	15.1	17.0	0.89	499	561	0
1	62	(−)		P	1	0	1	1	1	0	0					1	1	0	1	20.7	27.0	0.77	527	689	0
1	49	(−)		P	1	0	1	1	1	0	0					1	1	1	1	36.4	36.8	0.99	511	517	0
1	43	(−)		P	1	0	1	1	1	0	0					1	0	1	1	32.7	37.3	0.88	962	1098	20
1	53	(−)		C	1	0	0	1	0	1	0					1	0	0	1	30.7	40.0	0.77	505	658	0
1	50	(−)	ｹﾝｼｭﾂｾｽﾞ	P	1	0	1	1	1	0	0					1	1	0	1	28.7	26.2	1.09	615	562	0
1	54	13.6	ｹﾝｼｭﾂｾｽﾞ	P	1	0	1	1	1	0	0					1	1	1	1	27.5	37.0	0.74	332	448	0
1	57	6.4	ｹﾝｼｭﾂｾｽﾞ	P	1	0	1	1	1	0	0					0	1	1	1	31.8	26.4	1.20	381	317	0
1	54	(−)		P	1	0	1	1	1	0	0					1	1	1	1	40.4	32.5	1.24	634	510	0
1	45	(−)	ｹﾝｼｭﾂｾｽﾞ	P	1	0	1	1	1	0	0					1	1	1	1	39.5	26.8	1.47	592	402	0
1	62	(−)		P	1	0	1	1	1	0	0					1	1	1	1	35.7	25.6	1.40	431	309	20
1	41	(−)		P	1	0	1	1	1	0	0					1	0	0	1	44.2	29.3	1.51	963	639	0
1	51	(−)		P	1	0	1	1	1	0	0					1	1	0	1	29.4	29.4	1.00	995	994	20
1	63	(−)		P	1	0	1	1	1	0	0					1	1	1	1	17.2	25.5	0.67	163	242	0
1	54	87.9	ｹﾝｼｭﾂｾｽﾞ	P	1	0	1	1	1	0	0					1	1	0	1	25.0	39.7	0.63	541	857	0
1	40	(−)		P	1	0	1	1	1	0	0					1	1	0	1	12.5	41.4	0.30	143	475	55
1	48	(−)		P	1	0	1	1	1	0	0					0	1	1	1	33.0	32.7	1.01	515	510	75
1	53	(−)		C	1	0	0	1	0	1	0					1	1	0	1	14.4	29.5	0.49	273	558	46
1	60	(−)		P	1	0	1	1	1	0	0					1	1	1	1	29.9	43.1	0.69	395	569	25
1	65	(−)		P	1	0	1	1	1	0	0					1	0	1	1	25.0	31.5	0.80	383	482	20
1	46	(−)		P	1	0	1	1	1	0	0					1	1	0	1	32.7	31.9	1.02	603	589	0
1	43	(−)		P	1	0	1	1	1	0	0					1	1	0	1	34.2	28.6	1.20	1130	945	32
1	51	(−)		P	1	0	1	1	1	0	0					1	1	1	1	22.6	52.4	0.43	289	671	20
1	58	(−)		P	1	0	1	1	1	0	0					1	1	0	1	26.5	36.9	0.72	538	750	0
1	47	(−)		P	1	0	1	1	1	0	0					1	1	0	1	33.0	36.5	0.90	771	852	0
1	55	(−)		P	1	0	1	1	1	0	0					1	0	1	1	33.6	33.3	1.01	428	423	20
1	52	(−)		P	1	0	1	1	1	0	0					1	1	0	1	38.4	16.3	2.36	688	292	44
1	54	(−)	ｹﾝｼｭﾂｾｽﾞ	P	1	0	1	1	1	0	0					1	1	1	1	31.6	39.7	0.80	457	574	20
1	70	(−)		C	1	0	0	1	0	1	0					1	1	1	1	30.7	24.3	1.26	197	156	20
1	51	(−)	ｹﾝｼｭﾂｾｽﾞ	P	1	0	1	1	1	0	0					1	1	1	1	36.0	35.1	1.02	916	894	20
1	52	(−)		P	1	0	1	1	1	0	0					1	1	0	1	37.8	25.3	1.49	907	608	0
1	50	(−)		P	1	0	1	1	1	0	0					1	1	1	1	40.2	24.6	1.64	796	487	20
1	53	(−)		P	1	0	1	1	1	0	0					1	0	1	1	22.8	41.5	0.55	588	1071	0
1	55	(−)		P	1	0	1	1	1	0	0					0	0	1	0	28.6	26.0	1.10	547	498	0
1	46	(−)		C	1	0	0	1	0	1	0					1	1	0	1	43.7	30.1	1.45	882	608	30
1	42	(−)		P	1	0	1	1	1	0	0					1	0	0	1	43.3	32.8	1.32	1180	893	0
1	49	(−)		P	1	0	1	1	1	0	0					1	1	0	1	29.2	30.3	0.96	627	651	0
1	50	(−)	ｹﾝｼｭﾂｾｽﾞ	P	1	0	1	1	1	0	0					1	1	1	1	27.0	51.3	0.53	599	1139	20
1	43	(−)		P	1	0	1	1	1	0	0					1	1	0	1	42.1	23.9	1.76	900	510	47
1	46	(−)	ｹﾝｼｭﾂｾｽﾞ	P	1	0	1	1	1	0	0					1	1	1	1	32.1	24.1	1.33	781	586	0
1	57	(−)		P	1	0	1	1	1	0	0					1	1	0	1	34.2	47.8	0.72	526	735	0
1	56	(−)		P	1	0	1	1	1	0	0					0	0	1	0	49.6	29.9	1.66	829	501	20
1	47	(−)		P	1	0	1	1	1	0	0					1	1	1	1	29.6	40.1	0.74	388	526	0
1	43	(−)		P	1	0	1	1	1	0	0					1	1	1	1	31.5	44.8	0.70	582	827	0
1	55	(−)		P	1	0	1	1	1	0	0					0	0	1	0	32.5	25.1	1.29	656	508	0
1	42	(−)		P	1	0	1	1	1	0	0					1	1	0	1	29.9	30.2	0.99	748	756	20
1	47	(−)		P	1	0	1	1	1	0	0					1	1	0	1	35.6	26.0	1.37	1116	814	0
1	48	(−)		P	1	0	1	1	1	0	0					1	1	0	1	27.7	38.4	0.72	469	651	0
1	49	(−)		P	1	0	1	1	1	0	0					1	1	0	1	26.8	22.8	1.18	393	334	0
1	38		ｹﾝｼｭﾂｾｽﾞ	P	1	0	1	1	1	0	0					1	1	0	1	28.9	54.2	0.53	482	903	25
1	36	(−)		P	1	0	1	1	1	0	0					1	1	1	1	23.8	39.8	0.60	909	1515	37
1	46	(−)		P	1	0	1	1	1	0	0					1	0	1	1	29.1	44.5	0.65	531	812	0
1	42	83.7	ｹﾝｼｭﾂｾｽﾞ	P	1	0	1	1	1	0	0					1	1	0	1	37.3	30.1	1.24	672	541	0
1	44	(−)		C	1	0	0	1	0	1	0					1	1	0	1	33.8	33.5	1.01	751	747	20
1	74	(−)		P	1	0	1	1	1	0	0					0	0	1	0	26.4	16.6	1.59	622	392	20
1	51	(−)		P	1	0	1	1	1	0	0					1	1	1	1	28.3	30.5	0.93	717	773	0
1	51	(−)		C	1	0	0	1	0	1	0					1	1	0	1	30.9	36.7	0.84	878	1042	0
1	59	(−)		P	1	0	1	1	1	0	0					1	1	0	1	12.2	44.9	0.27	197	728	0

Sex	Age	HCV-Ab	HCVRNA	status	既感染あ	vaccine	HBsAbscr	HBcAbscr	seroconve	chronic in	Indetermin	observation	TAF(M)	TDF(M)	3TC(M)	TAF(1/0)	TDF(1/0)	3TC(1/0)	FTC	CD4%	CD8%	CD4/8	CD4_count	CD8_count	HIV_RNA
1	48	(−)	ケンシュツセス	P	1	0	1	1	1	0	0					0	0	1	0	34.8	22.2	1.57	236	150	23
1	51	(−)		P	1	0	1	1	1	0	0					0	0	1	0	32.6	24.9	1.31	511	390	0
1	58	(−)		P	1	0	1	1	1	0	0					1	1	0	1	32.9	47.5	0.69	847	1224	0
1	55	(−)		P	1	0	1	1	1	0	0					1	1	0	1	35.1	36.0	0.98	512	524	0
1	58	(−)		P	1	0	1	1	1	0	0					1	1	0	1	43.4	29.1	1.49	1279	859	20
1	59		7.4	P	1	0	1	1	1	0	0					1	0	1	1	30.5	23.7	1.29	435	338	35
1	65			P	1	0	1	1	1	0	0					0	1	1	1	14.6	48.0	0.30	333	1092	20
1	51	(−)		P	1	0	1	1	1	0	0					0	0	1	0	32.9	35.9	0.92	511	557	0
1	49	(−)		P	1	0	1	1	1	0	0					1	0	0	1	35.5	27.5	1.29	564	437	0
1	44	(−)	ケンシュツセス	P	1	0	1	1	1	0	0					0	0	1	0	36.9	25.2	1.46	675	461	20
1	39	(−)		P	1	0	1	1	1	0	0					1	1	0	1	31.1	41.2	0.76	728	963	0
1	59	(−)		P	1	0	1	1	1	0	0					1	1	0	1	29.0	31.9	0.91	663	730	20
1	41	(−)		P	1	0	1	1	1	0	0					1	1	0	1	48.2	29.3	1.65	873	531	20
1	54			P	1	0	1	1	1	0	0					0	1	1	1	21.0	39.6	0.53	368	694	0
1	55	(−)		P	1	0	1	1	1	0	0					1	0	1	1	22.9	38.3	0.60	440	735	0
1	51	(−)		P	1	0	1	1	1	0	0					0	0	1	0	30.0	37.6	0.80	304	380	0
1	49	(−)		P	1	0	1	1	1	0	0					1	1	0	1	28.8	33.4	0.86	797	926	0
2	42	(−)		P	1	0	1	1	1	0	0					1	1	0	1	50.6	28.6	1.77	669	378	0
1	52	(−)	ケンシュツセス	P	1	0	1	1	1	0	0					0	0	1	0	41.3	36.9	1.12	888	793	0
1	48	(−)		P	1	0	1	1	1	0	0					1	0	1	1	37.6	31.1	1.21	753	624	0
1	62	(−)		P	1	0	1	1	1	0	0					1	0	1	1	29.8	25.5	1.17	637	544	0
1	49	(−)		P	1	0	1	1	1	0	0					1	0	1	1	35.3	40.3	0.88	585	668	24
1	37	(−)	ケンシュツセス	P	1	0	1	1	1	0	0					1	1	0	1	38.8	31.8	1.22	888	728	0
1	41	4.2	ケンシュツセス	P	1	0	1	1	1	0	0					1	0	1	1	35.6	33.2	1.07	934	871	0
1	63	(−)		P	1	0	1	1	1	0	0					1	1	0	1	16.4	24.6	0.67	362	543	0
1	52	(−)	ケンシュツセス	P	1	0	1	1	1	0	0					0	0	1	0	27.8	18.6	1.49	673	452	0
1	65	(−)		P	1	0	1	1	1	0	0					1	1	0	1	19.9	44.5	0.45	388	868	0
1	57	(−)		P	1	0	1	1	1	0	0					0	0	1	0	28.9	28.6	1.01	480	475	60
1	50	(−)		P	1	0	1	1	1	0	0					1	1	0	1	35.2	34.1	1.03	412	399	43
1	41	(−)		P	1	0	1	1	1	0	0					1	1	0	1	23.5	59.9	0.39	347	884	0
1	46	(−)		P	1	0	1	1	1	0	0					0	0	1	0	27.5	35.5	0.78	440	568	0
1	45	(−)		P	1	0	1	1	1	0	0					1	1	1	1	34.6	22.6	1.53	922	601	20
1	47	(−)		P	1	0	1	1	1	0	0					1	1	0	1	32.5	25.1	1.29	864	668	20
1	44	(−)		P	1	0	1	1	1	0	0					0	0	1	0	29.3	18.5	1.58	397	251	0
1	48	5.4	ケンシュツセス	P	1	0	1	1	1	0	0					1	1	0	1	28.5	34.1	0.83	514	616	20
1	63	(−)		P	1	0	1	1	1	0	0					1	1	1	1	21.2	51.5	0.41	335	812	0
1	55	(−)		P	1	0	1	1	1	0	0					1	0	1	1	15.5	38.3	0.40	385	950	0
1	59	(−)		P	1	0	1	1	1	0	0					0	1	1	1	29.8	24.8	1.20	411	341	0
1	80	(−)		P	1	0	1	1	1	0	0					1	1	0	1	35.2	25.3	1.39	906	652	0
1	50			P	1	0	1	1	1	0	0					1	0	0	1	30.7	37.7	0.82	685	840	20
1	49	(−)		P	1	0	1	1	1	0	0					0	0	1	0	30.8	25.4	1.21	558	460	20
1	49	(−)		P	1	0	1	1	1	0	0					0	0	1	0	46.6	37.1	1.26	817	651	0
1	42	(−)		P	1	0	1	1	1	0	0					1	0	0	1	32.8	53.3	0.61	1323	2152	20
1	56	(−)	ケンシュツセス	P	1	0	1	1	1	0	0					1	1	0	1	38.1	27.1	1.40	1037	739	20
1	54	(−)		P	1	0	1	1	1	0	0					0	0	1	0	38.7	24.9	1.56	671	431	34
1	60	(−)		P	1	0	1	1	1	0	0					0	0	1	0	28.6	33.6	0.85	537	630	20
1	49	(−)		P	1	0	1	1	1	0	0					0	1	1	1	27.7	34.3	0.81	670	829	0
1	40	(−)		P	1	0	1	1	1	0	0					1	0	1	1	39.8	28.3	1.40	368	262	0
1	58	(−)	ケンシュツセス	P	1	0	1	1	1	0	0					1	1	1	1	38.0	34.8	1.09	1055	965	20
1	50	(−)		P	1	0	1	1	1	0	0					1	1	1	1	39.4	40.5	0.97	661	679	20
1	68	(−)		C	1	0	0	1	0	1	0					1	1	1	1	37.2	30.7	1.21	842	696	20
1	42	(−)		C	1	0	0	1	0	1	0					1	1	0	1	32.9	30.5	1.08	369	343	0
1	53	(−)		P	1	0	1	1	1	0	0					0	1	1	1	28.3	24.5	1.16	412	356	0
1	56	(−)		P	1	0	1	1	1	0	0					1	0	1	1	37.3	10.7	3.49	1113	318	20
1	46	(−)	ケンシュツセス	P	1	0	1	1	1	0	0					0	1	1	1	35.2	27.8	1.27	787	621	0
1	38		ケンシュツセス	P	1	0	1	1	1	0	0					1	0	1	1	35.5	35.1	1.01	790	780	0
1	66	(−)		P	1	0	1	1	1	0	0					0	0	1	0	21.4	24.1	0.89	414	467	36
1	56	(−)		P	1	0	1	1	1	0	0					0	0	1	0	29.4	34.8	0.85	627	742	0
1	50	(−)		P	1	0	1	1	1	0	0					1	1	0	1	39.3	27.8	1.42	467	330	0
1	48	(−)		P	1	0	1	1	1	0	0					1	1	1	1	39.3	37.5	1.05	654	624	20
1	54	17.3	ケンシュツセス	P	1	0	1	1	1	0	0					0	0	1	0	28.7	28.6	1.00	553	551	20

Sex	Age	HCV-Ab	HCVRNA	status	既感染あり	vaccine	HBsAbscr	HBcAbscr	seroconve	chronic in	Indetermi	observation	TAF(M)	TDF(M)	3TC(M)	TAF(1/0)	TDF(1/0)	3TC(1/0)	FTC	CD4%	CD8%	CD4/8	CD4_count	CD8_count	HIV_RNA
1	43	(−)		P	1	0	1	1	1	0	0					0	0	1	0	41.1	29.3	1.40	558	398	0
1	48			P	1	0	1	1	1	0	0					1	1	0	1	30.6	23.9	1.28	612	478	0
1	53	(−)		P	1	0	1	1	1	0	0					0	0	1	0	34.5	37.4	0.92	930	1009	0
1	45	(−)		C	1	0	0	1	0	1	0					1	1	0	1	28.4	32.5	0.87	695	796	20
1	44	(−)		P	1	0	1	1	1	0	0					0	0	1	0	30.7	44.2	0.69	548	790	0
1	50	(−)		P	1	0	1	1	1	0	0					1	1	0	1	33.1	23.2	1.43	496	347	20
1	50	(−)		P	1	0	1	1	1	0	0					0	0	1	0	32.3	39.0	0.83	673	813	0
1	53	(−)		P	1	0	1	1	1	0	0					1	1	1	1	51.4	25.8	2.00	1353	678	0
1	43	(−)		P	1	0	1	1	1	0	0					1	1	0	1	28.3	18.5	1.53	576	378	0
1	43	(−)		P	1	0	1	1	1	0	0					1	0	1	1	36.9	21.5	1.72	950	553	20
1	52			P	1	0	1	1	1	0	0					1	1	0	1	41.6	29.5	1.41	569	405	24
1	47			P	1	0	1	1	1	0	0					1	1	0	1	31.1	41.7	0.75	335	448	89
1	52		ケンシュツセズ	P	1	0	1	1	1	0	0					1	1	1	1	21.0	34.2	0.61	462	752	0
1	50		ケンシュツセズ	P	1	0	1	1	1	0	0					1	1	0	1	29.2	32.5	0.90	616	688	0
1	47	(−)		P	1	0	1	1	1	0	0					0	0	1	0	28.4	20.0	1.42	305	215	0
1	61	(−)		P	1	0	1	1	1	0	0					0	1	1	1	30.3	42.3	0.72	613	855	0
1	42	(−)		P	1	0	1	1	1	0	0					1	1	0	1	14.7	45.5	0.32	249	770	43
1	49	(−)		C	1	0	0	1	0	1	0					1	1	0	1	38.8	32.3	1.20	1159	962	75
1	43	(−)		P	1	0	1	1	1	0	0					1	0	1	1	25.0	29.7	0.84	579	690	0
1	58	(−)	ケンシュツセズ	C	1	0	0	1	0	1	0					1	1	0	1	32.3	37.8	0.86	821	960	0
1	37	(−)		P	1	0	1	1	1	0	0					1	1	0	1	36.1	28.7	1.26	644	512	0
1	47			P	1	0	1	1	1	0	0					1	1	0	1	27.8	40.3	0.69	462	670	0
1	41	(−)		P	1	0	1	1	1	0	0					1	1	0	1	30.7	34.5	0.89	412	463	0
1	43	(−)		P	1	0	1	1	1	0	0					1	0	1	1	37.8	28.7	1.32	837	636	24
1	42	(−)		P	1	0	1	1	1	0	0					0	0	1	0	27.5	35.6	0.77	844	1089	0
1	55	3.0	ケンシュツセズ	P	1	0	1	1	1	0	0					1	1	0	1	37.6	38.2	0.98	536	545	0
1	49	(−)		P	1	0	1	1	1	0	0					1	1	0	1	34.7	26.8	1.30	906	700	0
1	45	(−)		P	1	0	1	1	1	0	0					1	1	0	1	17.0	72.7	0.23	352	1501	0
1	53	(−)		P	1	0	1	1	1	0	0					1	1	1	1	25.9	40.6	0.64	517	811	20
1	52	(−)		P	1	0	1	1	1	0	0					1	1	0	1	26.9	35.0	0.77	599	779	20
1	45	(−)		P	1	0	1	1	1	0	0					0	1	1	1	27.5	45.2	0.61	889	1460	0
1	41	(−)		P	1	0	1	1	1	0	0					1	1	1	1	38.2	26.5	1.44	755	524	20
1	49	(−)	ケンシュツセズ	P	1	0	1	1	1	0	0					0	1	1	1	33.9	35.1	0.97	1068	1104	0
1	46	(−)	ケンシュツセズ	P	1	0	1	1	1	0	0					1	1	1	1	26.0	27.2	0.95	669	702	20
1	41	(−)		P	1	0	1	1	1	0	0					1	1	0	1	25.5	35.8	0.71	664	930	0
1	52	17.9	ケンシュツセズ	P	1	0	1	1	1	0	0					1	1	1	1	19.8	56.8	0.35	464	1331	960000
1	47	(−)		P	1	0	1	1	1	0	0					0	1	1	1	38.7	32.5	1.19	579	487	0
1	45	(−)		P	1	0	1	1	1	0	0					1	1	0	1	22.4	46.0	0.49	643	1320	0
1	37	1.4	ケンシュツセズ	C	1	0	0	1	0	1	0					1	1	0	1	15.0	50.3	0.30	487	1638	110
1	83	(−)		P	1	0	1	1	1	0	0					1	0	1	1	17.5	55.0	0.32	409	1285	73
1	53	(−)		P	1	0	1	1	1	0	0					1	1	1	1	28.3	41.5	0.68	520	761	20
1	45	(−)		P	1	0	1	1	1	0	0					1	1	0	1	27.0	36.9	0.73	766	1047	39
1	49	(−)		P	1	0	1	1	1	0	0					1	1	1	1	25.2	22.3	1.13	726	644	0
1	62	(−)		P	1	0	1	1	1	0	0					1	0	0	1	23.5	38.1	0.62	382	618	0
1	42	6.7	ケンシュツセズ	P	1	0	1	1	1	0	0					1	1	0	1	23.4	41.2	0.57	579	1021	22
1	53	(−)	ケンシュツセズ	P	1	0	1	1	1	0	0					1	0	1	1	20.5	38.9	0.53	280	533	20
1	36			P	1	0	1	1	1	0	0					1	1	0	1	27.2	39.1	0.70	625	899	0
1	68			P	1	0	1	1	1	0	0					0	0	1	0	12.8	16.2	0.79	177	224	22
1	53	(−)		P	1	0	1	1	1	0	0					1	0	1	1	7.7	44.8	0.17	286	1661	59
1	48	19.2	ケンシュツセズ	P	1	0	1	1	1	0	0					1	1	0	1	43.5	22.1	1.97	719	366	0
1	50	(−)		P	1	0	1	1	1	0	0					0	0	1	0	20.0	30.8	0.65	375	579	0
1	45	(−)		P	1	0	1	1	1	0	0					1	1	0	1	34.4	30.6	1.13	671	596	0
1	39	(−)		P	1	0	1	1	1	0	0					1	1	0	1	25.6	29.6	0.87	525	606	0
1	51	(−)		P	1	0	1	1	1	0	0					1	1	0	1	38.3	24.5	1.56	830	531	26
1	48	1.1	ケンシュツセズ	P	1	0	1	1	1	0	0					1	1	0	1	35.3	37.4	0.95	835	883	20
1	50	(−)		P	1	0	1	1	1	0	0					1	1	0	1	31.3	27.8	1.13	655	581	0
1	39	14.5	ケンシュツセズ	P	1	0	1	1	1	0	0					1	1	0	1	38.9	32.6	1.19	888	745	0
1	54	(−)		C	1	0	0	1	0	1	0					1	1	1	1	24.6	41.9	0.59	1356	2313	20
1	45			P	1	0	1	1	1	0	0					1	1	1	1	30.9	27.2	1.14	677	596	0
1	66	(−)		P	1	0	1	1	1	0	0					0	1	1	1	51.5	20.0	2.57	544	211	20
1	47	(−)		P	1	0	1	1	1	0	0					1	1	0	1	22.9	38.1	0.60	404	674	21

Sex	Age	HCV-Ab	HCVRNA	status	既感染あ	vaccine	HBsAbscr	HBcAbscr	seroconve	chronic in	Indetermi	observation	TAF(M)	TDF(M)	3TC(M)	TAF(1/0)	TDF(1/0)	3TC(1/0)	FTC	CD4%	CD8%	CD4/8	CD4_count	CD8_count	HIV_RNA
1	47	(−)		P	1	0	1	1	1	0	0					0	1	1	1	18.5	39.4	0.47	146	311	20
1	61	(−)		P	1	0	1	1	1	0	0					1	1	0	1	30.5	28.6	1.07	418	391	0
1	48	(−)		P	1	0	1	1	1	0	0					1	0	1	1	41.2	33.3	1.23	469	380	0
1	52	(−)		P	1	0	1	1	1	0	0					0	0	1	0	29.8	27.5	1.09	694	638	0
1	49	(−)		P	1	0	1	1	1	0	0					0	0	1	0	36.0	36.7	0.98	565	576	0
1	47	(−)		P	1	0	1	1	1	0	0					0	1	1	1	22.3	52.3	0.43	430	1011	0
1	56	(−)		P	1	0	1	1	1	0	0					1	0	1	1	46.0	28.9	1.59	777	488	0
1	49	(−)		P	1	0	1	1	1	0	0					1	1	0	1	23.0	36.2	0.63	390	614	28
1	41	(−)		P	1	0	1	1	1	0	0					1	1	1	1	47.8	18.0	2.66	698	262	0
1	38	(−)		P	1	0	1	1	1	0	0					1	0	0	1	25.6	41.0	0.63	438	701	25
1	58	(−)		P	1	0	1	1	1	0	0					1	1	1	1	21.2	26.2	0.81	182	224	20
1	69	(−)		P	1	0	1	1	1	0	0					1	1	1	1	29.9	25.8	1.16	589	509	20
1	55	(−)		P	1	0	1	1	1	0	0					1	1	0	1	18.1	25.8	0.70	184	262	20
1	54	(−)		P	1	0	1	1	1	0	0					1	0	1	1	26.5	29.7	0.89	385	431	0
1	55	(−)		P	1	0	1	1	1	0	0					1	0	1	1	24.2	32.2	0.75	650	865	0
1	54	(−)		P	1	0	1	1	1	0	0					1	1	1	1	25.4	29.4	0.86	487	564	0
1	56	(−)		P	1	0	1	1	1	0	0					0	1	1	1	37.5	40.5	0.93	724	782	46
1	37	(−)		P	1	0	1	1	1	0	0					1	1	0	1	32.7	37.9	0.86	579	672	0
1	45	(−)		P	1	0	1	1	1	0	0					1	1	1	1	32.2	49.0	0.66	448	680	20
1	40	(−)		P	1	0	1	1	1	0	0					1	1	0	1	26.7	36.0	0.74	430	580	20
1	50	(−)		P	1	0	1	1	1	0	0					0	0	1	0	28.5	34.5	0.83	425	515	0
1	43	(−)		P	1	0	1	1	1	0	0					0	0	1	0	34.8	40.8	0.85	578	676	0
1	47	(−)		P	1	0	1	1	1	0	0					1	1	1	1	41.7	32.6	1.28	739	577	0
1	42	(−)		P	1	0	1	1	1	0	0					1	1	1	1	30.6	22.5	1.36	539	395	0
1	41			P	1	0	1	1	1	0	0					1	1	0	1	33.1	22.5	1.48	960	651	54
1	54	(−)		P	1	0	1	1	1	0	0					1	1	0	1	36.7	30.4	1.20	841	699	20
1	32	(−)		P	1	0	1	1	1	0	0					1	0	0	1	26.5	33.6	0.79	519	659	0
1	48	(−)		P	1	0	1	1	1	0	0					0	0	1	0	25.1	39.0	0.64	517	803	0
1	40	(−)		C	1	0	0	1	0	1	0					1	1	0	1	34.9	40.5	0.86	576	669	24
1	47	(−)		P	1	0	1	1	1	0	0					1	0	1	1	36.5	35.2	1.04	705	679	0
1	47			P	1	0	1	1	1	0	0					1	1	0	1	35.6	36.5	0.98	412	423	0
1	67	(−)		P	1	0	1	1	1	0	0					0	1	1	1	27.5	31.8	0.87	767	885	20
1	46	(−)		P	1	0	1	1	1	0	0					1	1	0	1	33.5	31.1	1.08	968	898	36
1	37	(−)		P	1	0	1	1	1	0	0					1	1	0	1	12.0	49.3	0.24	257	1054	0
1	44	(−)		C	1	0	0	1	0	1	0					1	1	0	1	33.3	22.8	1.46	1000	685	0
1	39	(−)		P	1	0	1	1	1	0	0					1	1	0	1	34.0	20.7	1.64	501	305	0
1	51	(−)		P	1	0	1	1	1	0	0					0	1	1	1	34.3	27.7	1.24	739	596	20
1	58	2.1	ｹﾝｼｭﾂｾｽﾞ	P	1	0	1	1	1	0	0					1	1	1	1	21.6	35.4	0.61	527	865	0
1	48	(−)		P	1	0	1	1	1	0	0					0	0	1	0	33.3	27.6	1.21	576	478	0
1	67	(−)		P	1	0	1	1	1	0	0					0	0	1	0	29.5	11.6	2.55	736	289	0
1	52	(−)		P	1	0	1	1	1	0	0					1	1	0	1	34.7	32.3	1.08	542	504	0
1	39	(−)		P	1	0	1	1	1	0	0					1	1	0	1	23.5	44.1	0.53	635	1192	0
1	49			P	1	0	1	1	1	0	0					0	0	1	0	29.6	34.3	0.86	1010	1180	22
1	42	(−)		P	1	0	1	1	1	0	0					1	1	0	1	25.7	43.6	0.59	504	855	0
1	47	(−)		P	1	0	1	1	1	0	0					1	1	1	1	32.6	43.9	0.74	505	681	0
1	50	(−)		P	1	0	1	1	1	0	0					1	1	1	1	32.5	23.0	1.41	405	288	0
1	51	(−)		P	1	0	1	1	1	0	0					0	0	1	0	20.6	53.8	0.38	322	842	0
1	41	(−)		P	1	0	1	1	1	0	0					1	1	0	1	38.1	31.1	1.22	778	636	0
1	42			P	1	0	1	1	1	0	0					1	1	0	1	22.3	38.2	0.58	307	527	20
1	52	(−)		P	1	0	1	1	1	0	0					1	1	1	1	27.3	34.9	0.78	456	583	0
1	75	(−)		P	1	0	1	1	1	0	0					1	1	0	1	28.8	32.5	0.89	466	526	0
1	33	(−)		P	1	0	1	1	1	0	0					1	0	0	1	34.5	39.7	0.87	784	902	0
1	34	(−)		P	1	0	1	1	1	0	0					1	1	0	1	52.3	21.1	2.48	1098	443	0
1	52			P	1	0	1	1	1	0	0					1	0	0	1	13.6	58.8	0.23	583	2528	0
1	36	(−)		P	1	0	1	1	1	0	0					0	0	1	0	25.1	19.2	1.30	706	541	0
1	55	(−)		P	1	0	1	1	1	0	0					0	0	1	0	24.0	17.7	1.36	754	557	0
1	66	(−)		P	1	0	1	1	1	0	0					1	1	0	1	18.8	34.2	0.55	321	581	0
1	35	(−)		P	1	0	1	1	1	0	0					1	0	1	1	15.7	54.4	0.29	177	613	20
1	38	(−)		P	1	0	1	1	1	0	0					0	0	1	0	37.8	28.8	1.31	577	441	20
1	50	(−)		P	1	0	1	1	1	0	0					1	1	0	1	36.1	23.2	1.56	812	522	20
1	39	(−)		P	1	0	1	1	1	0	0					1	0	0	1	27.1	54.7	0.50	900	1815	42

Sex	Age	HCV-Ab	HCVRNA	status	既感染あ	vaccine	HBsAbscr	HBcAbscr	seroconve	chronic in	Indetermir	observation	TAF(M)	TDF(M)	3TC(M)	TAF(1/0)	TDF(1/0)	3TC(1/0)	FTC	CD4%	CD8%	CD4/8	CD4_count	CD8_count	HIV_RNA
1	39	(−)		P	1	0	1	1	1	0	0					1	1	1	1	33.9	29.6	1.15	729	637	0
1	45	(−)		P	1	0	1	1	1	0	0					1	1	0	1	38.8	30.7	1.26	509	403	0
1	61	(−)		P	1	0	1	1	1	0	0					1	1	1	1	19.9	22.4	0.89	459	516	30
2	56	(−)		P	1	0	1	1	1	0	0					1	1	1	1	41.9	27.3	1.54	878	572	0
1	41	(−)		C	1	0	0	1	0	1	0					1	1	0	1	26.4	29.1	0.91	381	419	0
1	37	(−)		P	1	0	1	1	1	0	0					0	0	1	0	30.1	34.4	0.88	782	892	20
1	44	(−)		P	1	0	1	1	1	0	0					1	1	0	1	40.0	25.9	1.54	993	645	0
1	59	(−)		C	1	0	0	1	0	1	0					1	0	0	1	34.8	34.9	1.00	739	740	0
1	53	(−)		P	1	0	1	1	1	0	0					1	1	0	1	47.1	21.7	2.17	759	350	20
1	40	(−)		P	1	0	1	1	1	0	0					1	0	0	1	37.1	27.5	1.35	1278	948	63
1	41	(−)		P	1	0	1	1	1	0	0					1	1	0	1	26.7	28.1	0.95	918	968	22
1	30	(−)		P	1	0	1	1	1	0	0					1	0	0	1	29.0	16.7	1.74	486	279	0
1	59	(−)		P	1	0	1	1	1	0	0					1	1	0	1	30.4	36.7	0.83	448	542	83
1	54	(−)		P	1	0	1	1	1	0	0					1	1	0	1	26.6	43.3	0.61	427	695	20
1	54	(−)		P	1	0	1	1	1	0	0					1	1	1	1	30.2	23.1	1.31	703	537	0
1	34		ｹﾝｼｭﾂｾｽ	P	1	0	1	1	1	0	0					1	1	0	1	31.1	33.0	0.94	734	779	0
1	53			P	1	0	1	1	1	0	0					0	0	1	0	37.2	37.4	0.99	1071	1079	0
1	40			P	1	0	1	1	1	0	0					1	1	0	1	33.2	37.5	0.89	647	731	0
1	45		ｹﾝｼｭﾂｾｽ	P	1	0	1	1	1	0	0					0	0	1	0	20.3	57.8	0.35	596	1698	0
1	48	(−)		P	1	0	1	1	1	0	0					1	1	1	1	39.3	37.1	1.06	771	729	20
1	45	(−)		P	1	0	1	1	1	0	0					1	1	0	1	38.6	20.9	1.85	1181	638	0
1	48			P	1	0	1	1	1	0	0					1	1	1	1	34.5	39.5	0.87	436	499	0
1	45	(−)		C	1	0	0	1	0	1	0					1	0	0	1	36.5	33.4	1.09	703	644	20
1	51	(−)		P	1	0	1	1	1	0	0					1	1	0	1	26.1	33.8	0.77	391	507	20
1	58	(−)		P	1	0	1	1	1	0	0					1	0	1	1	33.7	24.3	1.39	529	381	0
1	44	(−)		P	1	0	1	1	1	0	0					0	0	1	0	31.0	39.5	0.78	867	1106	0
1	55	(−)		P	1	0	1	1	1	0	0					1	1	0	1	36.9	24.2	1.53	412	270	0
1	43	3.2	ｹﾝｼｭﾂｾｽ	P	1	0	1	1	1	0	0					1	1	0	1	31.1	38.5	0.81	507	627	0
1	41	(−)		C	1	0	0	1	0	1	0					1	0	0	1	40.5	25.2	1.60	1065	664	20
1	69	(−)		P	1	0	1	1	1	0	0					1	1	1	1	31.0	24.0	1.29	366	284	0
1	65	(−)		C	1	0	0	1	0	1	0					1	0	1	1	36.1	28.1	1.28	1136	885	20
1	42	(−)		P	1	0	1	1	1	0	0					0	0	1	0	26.5	32.0	0.83	557	671	20
1	41	(−)		P	1	0	1	1	1	0	0					1	1	0	1	34.5	24.6	1.40	713	508	0
1	42	(−)		P	1	0	1	1	1	0	0					1	0	1	1	33.0	38.8	0.85	588	691	20
1	47	(−)		P	1	0	1	1	1	0	0					1	1	0	1	30.1	26.7	1.13	447	396	0
1	44	(−)		P	1	0	1	1	1	0	0					0	0	1	0	34.1	24.1	1.42	561	395	0
1	53	5.4	ｹﾝｼｭﾂｾｽ	P	1	0	1	1	1	0	0					1	1	0	1	32.6	38.0	0.86	520	606	0
1	46	(−)		P	1	0	1	1	1	0	0					0	1	1	1	32.0	33.3	0.96	552	575	20
1	48	(−)		P	1	0	1	1	1	0	0					1	1	0	1	31.2	36.2	0.86	587	680	0
1	58	(−)		P	1	0	1	1	1	0	0					1	1	1	1	42.1	41.0	1.03	1109	1081	0
1	46		2.8	P	1	0	1	1	1	0	0					1	1	0	1	15.3	49.6	0.31	163	530	44000
1	42	(−)		C	1	0	0	1	0	1	0					1	1	0	1	27.3	42.9	0.64	580	913	0
1	57	(−)		P	1	0	1	1	1	0	0					0	0	1	0	42.9	23.2	1.85	1080	583	45
1	42	(−)		P	1	0	1	1	1	0	0					1	1	0	1	17.0	32.8	0.52	286	552	0
1	54	(−)		P	1	0	1	1	1	0	0					1	1	0	1	32.0	38.4	0.83	894	1071	0
1	42			P	1	0	1	1	1	0	0					1	0	0	1	30.6	33.4	0.92	490	535	20
1	54	(−)		P	1	0	1	1	1	0	0					1	1	1	1	23.3	22.4	1.04	412	396	0
1	49	(−)		P	1	0	1	1	1	0	0					1	1	0	1	23.5	38.5	0.61	488	802	20
1	52			P	1	0	1	1	1	0	0					0	0	1	0	41.9	25.8	1.62	563	347	0
1	38	(−)		P	1	0	1	1	1	0	0					1	1	1	1	28.1	22.7	1.24	636	513	22
1	37	(−)		P	1	0	1	1	1	0	0					1	1	0	1	32.7	38.7	0.84	707	838	26
1	50	(−)		P	1	0	1	1	1	0	0					1	1	0	1	26.0	26.9	0.97	481	497	20
1	43	(−)		P	1	0	1	1	1	0	0					1	1	0	1	29.5	31.4	0.94	411	438	0
1	53	(−)		P	1	0	1	1	1	0	0					1	1	0	1	28.1	22.0	1.28	547	429	20
1	49	(−)		P	1	0	1	1	1	0	0					1	1	0	1	29.8	30.1	0.99	469	474	0
1	60	(−)		P	1	0	1	1	1	0	0					1	1	0	1	21.9	28.1	0.78	335	431	20
1	54	(−)		P	1	0	1	1	1	0	0					1	1	0	1	22.1	49.8	0.44	473	1066	550
1	48	(−)		P	1	0	1	1	1	0	0					1	1	1	1	31.8	35.3	0.90	623	693	0
1	33	(−)		P	1	0	1	1	1	0	0					1	1	0	1	22.0	37.6	0.58	500	857	20
1	46	(−)		P	1	0	1	1	1	0	0					1	1	0	1	34.3	24.7	1.39	1033	744	22
1	59	(−)		P	1	0	1	1	1	0	0					0	1	1	1	49.4	20.9	2.36	1051	445	0

Sex	Age	HCV-Ab	HCVRNA	status	既感染あ	vaccine	HBsAbscr	HBcAbscr	seroconve	chronic in	Indetermi	observation	TAF(M)	TDF(M)	3TC(M)	TAF(1/0)	TDF(1/0)	3TC(1/0)	FTC	CD4%	CD8%	CD4/8	CD4_count	CD8_count	HIV_RNA
1	43			P	1	0	1	1	1	0	0					0	0	1	0	38.0	32.1	1.19	678	572	0
1	45	(−)		P	1	0	1	1	1	0	0					1	1	0	1	33.7	33.7	1.00	383	382	0
1	46			P	1	0	1	1	1	0	0					1	1	0	1	32.2	45.7	0.71	704	997	0
1	47	(−)		P	1	0	1	1	1	0	0					1	1	0	1	37.8	38.4	0.99	524	532	0
1	44	(−)		P	1	0	1	1	1	0	0					1	1	0	1	35.3	24.2	1.46	894	612	0
1	38	(−)		P	1	0	1	1	1	0	0					1	1	0	1	35.3	31.4	1.12	741	659	0
1	41	(−)		P	1	0	1	1	1	0	0					1	1	0	1	29.4	26.3	1.12	680	608	0
1	44	(−)		P	1	0	1	1	1	0	0					1	1	0	1	49.4	18.6	2.66	851	320	0
1	45	(−)		P	1	0	1	1	1	0	0					1	1	0	1	35.1	24.1	1.45	601	413	0
1	51			P	1	0	1	1	1	0	0					1	1	0	1	36.0	25.1	1.43	1012	707	20
1	41	(−)		P	1	0	1	1	1	0	0					1	1	0	1	23.2	18.6	1.25	514	412	0
1	39	(−)		P	1	0	1	1	1	0	0					1	1	0	1	31.6	39.6	0.80	572	718	20
1	80	(−)		P	1	0	1	1	1	0	0					1	1	1	1	21.6	19.4	1.12	367	329	33
1	31	(−)		P	1	0	1	1	1	0	0					1	1	0	1	37.6	30.3	1.24	512	412	0
1	33			P	1	0	1	1	1	0	0					1	1	0	1	23.7	44.3	0.53	667	1248	0
1	42	(−)		P	1	0	1	1	1	0	0					1	1	0	1	20.1	41.8	0.48	441	918	0
1	36	(−)		P	1	0	1	1	1	0	0					1	1	0	1	39.5	33.9	1.16	556	478	0
1	37			P	1	0	1	1	1	0	0					0	0	1	0	34.0	28.4	1.20	693	578	0
1	36	(−)		P	1	0	1	1	1	0	0					1	0	1	1	32.6	39.5	0.83	716	866	0
1	53	(−)		P	1	0	1	1	1	0	0					1	1	0	1	27.5	43.6	0.63	677	1075	20
1	48		ケンシュツセス	P	1	0	1	1	1	0	0					0	0	1	0	41.7	35.7	1.17	280	240	0
1	62	(−)		P	1	0	1	1	1	0	0					1	1	0	1	25.1	40.0	0.63	578	923	38
1	42	(−)		P	1	0	1	1	1	0	0					1	0	0	1	41.3	22.8	1.81	999	551	0
1	56	(−)		P	1	0	1	1	1	0	0					0	0	1	0	38.8	42.7	0.91	859	945	20
1	67	(−)		P	1	0	1	1	1	0	0					1	1	1	1	28.1	35.2	0.80	520	651	0
1	51	(−)		P	1	0	1	1	1	0	0					0	0	1	0	31.6	43.0	0.74	388	527	30
1	32	(−)		P	1	0	1	1	1	0	0					1	1	0	1	34.9	27.5	1.27	567	446	0
1	34	47.4	6.6	P	1	0	1	1	1	0	0					0	0	1	0	10.0	60.6	0.16	119	724	330
1	48	(−)	ケンシュツセス	C	1	0	0	1	0	1	0					1	1	1	1	36.1	32.1	1.13	799	709	0
1	38	(−)		P	1	0	1	1	1	0	0					1	1	0	1	20.1	46.7	0.43	395	920	0
1	50	(−)		P	1	0	1	1	1	0	0					1	1	0	1	23.5	46.2	0.51	312	612	0
1	49	(−)		P	1	0	1	1	1	0	0					1	1	0	1	29.6	24.3	1.22	434	357	0
1	43	(−)	ケンシュツセス	C	1	0	0	1	0	1	0					1	1	0	1	25.3	25.0	1.01	640	634	0
1	56	(−)		P	1	0	1	1	1	0	0					0	0	1	0	34.4	28.2	1.22	615	504	0
1	41	(−)		P	1	0	1	1	1	0	0					1	1	0	1	39.6	31.4	1.26	837	665	0
1	62	(−)		P	1	0	1	1	1	0	0					1	0	1	1	35.7	32.5	1.10	608	554	31
1	50	(−)		P	1	0	1	1	1	0	0					1	1	0	1	35.3	23.0	1.54	613	398	0
1	57			P	1	0	1	1	1	0	0					0	0	1	0	20.7	38.3	0.54	372	687	0
1	41	(−)		P	1	0	1	1	1	0	0					1	1	0	1	20.4	41.0	0.50	320	642	0
1	30	(−)		P	1	0	1	1	1	0	0					1	0	0	1	22.5	39.3	0.57	358	627	0
1	68	(−)		P	1	0	1	1	1	0	0					1	1	0	1	17.2	39.5	0.43	352	808	0
1	44	(−)		P	1	0	1	1	1	0	0					1	1	0	1	27.6	23.3	1.18	578	488	22
1	59	(−)		P	1	0	1	1	1	0	0					1	0	1	1	34.5	20.9	1.65	640	388	190
1	34	(−)		P	1	0	1	1	1	0	0					1	1	0	1	35.4	32.2	1.10	727	662	41
1	49	(−)		P	1	0	1	1	1	0	0					1	1	0	1	24.0	20.7	1.16	731	629	0
1	55			C	1	0	0	1	0	1	0					1	1	0	1	30.2	30.7	0.98	399	407	20
1	43	(−)		P	1	0	1	1	1	0	0					1	1	0	1	39.5	28.4	1.39	804	578	45
1	41	(−)		P	1	0	1	1	1	0	0					0	0	1	0	25.4	38.7	0.65	684	1046	0
1	31	(−)		P	1	0	1	1	1	0	0					1	0	1	1	31.9	42.6	0.75	795	1061	0
1	50	(−)		P	1	0	1	1	1	0	0					0	0	1	0	29.3	25.1	1.17	736	630	0
1	38	(−)		P	1	0	1	1	1	0	0					1	1	0	1	34.0	37.7	0.90	320	355	0
1	44	(−)		P	1	0	1	1	1	0	0					1	1	0	1	25.9	56.7	0.46	570	1248	0
1	54	(−)		P	1	0	1	1	1	0	0					0	0	1	0	25.8	42.4	0.61	361	593	0
1	44	(−)		P	1	0	1	1	1	0	0					1	1	0	1	11.5	52.0	0.22	396	1792	0
1	40	(−)		P	1	0	1	1	1	0	0					1	1	0	1	30.2	35.1	0.86	449	522	0
1	45	(−)		C	1	0	0	1	0	1	0					1	1	0	1	26.5	35.4	0.75	374	499	0
1	42	(−)		P	1	0	1	1	1	0	0					1	0	0	1	36.7	31.8	1.16	1063	921	0
1	56	(−)		P	1	0	1	1	1	0	0					0	0	1	0	24.3	54.7	0.44	373	839	0
1	52		ケンシュツセス	P	1	0	1	1	1	0	0					1	1	0	1	36.5	23.9	1.53	563	368	0
1	45	(−)		P	1	0	1	1	1	0	0					0	1	1	1	20.6	32.6	0.63	500	793	20
1	47			P	1	0	1	1	1	0	0					1	1	0	1	7.3	55.3	0.13	94	715	30000

Sex	Age	HCV-Ab	HCVRNA	status	既感染あ	vaccine	HBsAbscr	HBcAbscr	seroconve	chronic in	Indetermir	observation	TAF(M)	TDF(M)	3TC(M)	TAF(1/0)	TDF(1/0)	3TC(1/0)	FTC	CD4%	CD8%	CD4/8	CD4_count	CD8_count	HIV_RNA
1	35	(−)		C	1	0	0	1	0	1	0					1	1	0	1	26.1	29.2	0.89	636	711	0
1	48	(−)		P	1	0	1	1	1	0	0					1	1	1	1	27.8	33.3	0.83	604	725	0
1	26	(−)		P	1	0	1	1	1	0	0					1	0	0	1	29.6	29.4	1.01	679	675	20
1	40	(−)		C	1	0	0	1	0	1	0					0	0	1	0	40.4	31.0	1.30	950	729	0
1	44	(−)		P	1	0	1	1	1	0	0					1	1	0	1	20.8	24.8	0.84	588	702	26
1	47	2.7	ケンシュツセス	P	1	0	1	1	1	0	0					1	0	0	1	43.5	30.4	1.43	587	410	0
1	51	(−)		P	1	0	1	1	1	0	0					0	0	1	0	34.6	26.1	1.33	351	264	0
1	46	(−)		P	1	0	1	1	1	0	0					1	1	0	1	28.9	39.4	0.73	616	840	20
1	34	(−)		P	1	0	1	1	1	0	0					0	0	1	0	22.5	50.3	0.45	827	1849	0
1	31			P	1	0	1	1	1	0	0					0	0	1	0	34.3	37.5	0.91	586	641	0
1	34	(−)		C	1	0	0	1	0	1	0					1	1	0	1	27.5	41.6	0.66	477	722	29
1	38	(−)		P	1	0	1	1	1	0	0					1	1	0	1	34.5	31.8	1.09	999	919	0
1	55	(−)		P	1	0	1	1	1	0	0					1	0	1	1	41.3	24.6	1.68	1236	738	20
2	49	(−)		P	1	0	1	1	1	0	0					1	1	0	1	41.9	29.4	1.43	1272	892	0
1	33	(−)		P	1	0	1	1	1	0	0					1	1	0	1	30.3	32.3	0.94	481	513	0
1	50	(−)		P	1	0	1	1	1	0	0					1	1	1	1	17.5	40.0	0.44	216	494	22
1	43	(−)		P	1	0	1	1	1	0	0					1	0	0	1	15.4	40.2	0.38	310	807	0
1	37	(−)		P	1	0	1	1	1	0	0					1	0	1	1	23.0	31.8	0.72	328	454	41000
1	34	(−)	ケンシュツセス	P	1	0	1	1	1	0	0					1	1	0	1	31.9	25.6	1.25	731	587	0
1	34	(−)		C	1	0	0	1	0	1	0					1	1	0	1	25.2	29.0	0.87	413	475	0
1	32	(−)	ケンシュツセス	P	1	0	1	1	1	0	0					0	0	1	0	33.8	32.9	1.03	778	757	20
1	41	(−)		P	1	0	1	1	1	0	0					1	0	1	1	24.1	48.3	0.50	656	1314	55
1	49	(−)		P	1	0	1	1	1	0	0					1	1	0	1	38.4	26.3	1.46	706	484	0
1	53	(−)	ケンシュツセス	P	1	0	1	1	1	0	0					0	0	1	0	12.4	49.7	0.25	228	915	0
1	40	(−)		P	1	0	1	1	1	0	0					1	0	1	1	31.7	44.3	0.72	612	855	0
1	57	(−)		P	1	0	1	1	1	0	0					1	0	1	1	20.0	37.0	0.54	306	566	180
1	82			P	1	0	1	1	1	0	0					0	1	1	1	32.4	41.0	0.79	479	606	0
1	44		ケンシュツセス	P	1	0	1	1	1	0	0					1	0	0	1	27.1	31.5	0.86	669	778	20
1	35	(−)		P	1	0	1	1	1	0	0					1	0	0	1	46.7	30.8	1.52	827	546	21
1	60	(−)	ケンシュツセス	P	1	0	1	1	1	0	0					0	0	1	0	17.5	43.3	0.41	338	835	83
1	56	(−)		P	1	0	1	1	1	0	0					0	0	1	0	19.9	30.9	0.64	349	542	0
1	49	(−)		P	1	0	1	1	1	0	0					1	0	0	1	24.1	27.6	0.87	440	504	79
1	67	(−)		P	1	0	1	1	1	0	0					1	1	1	1	29.3	39.1	0.75	492	656	0
1	39	(−)		P	1	0	1	1	1	0	0					1	0	0	1	33.2	38.4	0.86	519	601	0
1	34	(−)		C	1	0	0	1	0	1	0					1	0	0	1	38.2	28.9	1.32	1142	864	70
1	49	(−)		P	1	0	1	1	1	0	0					1	0	1	1	19.5	22.9	0.85	451	530	42
1	46	(−)		P	1	0	1	1	1	0	0					1	0	0	1	13.9	45.5	0.30	192	631	0
1	42	(−)		P	1	0	1	1	1	0	0					1	0	0	1	16.6	39.4	0.42	423	1002	71
1	51	(−)		P	1	0	1	1	1	0	0					0	1	1	1	22.3	28.4	0.79	637	810	22
1	40	(−)		P	1	0	1	1	1	0	0					1	1	0	1	22.6	49.7	0.45	593	1306	0
1	59	(−)		P	1	0	1	1	1	0	0					1	1	0	1	27.0	20.0	1.35	844	624	0
1	41	(−)		P	1	0	1	1	1	0	0					1	0	0	1	28.8	39.3	0.73	383	521	20
1	32	(−)		P	1	0	1	1	1	0	0					1	0	1	1	32.8	30.4	1.08	492	455	0
1	43	(−)		P	1	0	1	1	1	0	0					1	0	1	1	24.8	25.4	0.98	477	488	0
1	32	(−)		P	1	0	1	1	1	0	0					1	1	0	1	33.2	27.3	1.22	757	621	0
1	51	(−)		P	1	0	1	1	1	0	0					1	0	0	1	19.0	30.6	0.62	515	827	0
1	43			C	1	0	0	1	0	1	0					1	0	0	1	31.0	42.9	0.72	841	1164	0
1	66	(−)		P	1	0	1	1	1	0	0					1	0	0	1	33.3	20.3	1.64	579	353	130
2	62	(−)		C	1	0	0	1	0	1	0					1	0	0	1	17.9	43.1	0.42	349	839	0
1	43	(−)		C	1	0	0	1	0	1	0					1	1	0	1	32.9	37.8	0.87	384	442	20
1	30	(−)		P	1	0	1	1	1	0	0					1	0	0	1	46.1	29.1	1.58	902	570	0
1	28	(−)		P	1	0	1	1	1	0	0					0	0	1	0	25.0	29.4	0.85	617	726	0
1	44			P	1	0	1	1	1	0	0					0	0	1	0	41.1	23.1	1.78	820	461	0
1	29	(−)		P	1	0	1	1	1	0	0					1	0	0	1	36.7	26.1	1.40	593	422	0
1	44			P	1	0	1	1	1	0	0					1	1	0	1	29.2	17.4	1.68	1075	641	0
1	49	(−)		P	1	0	1	1	1	0	0					1	1	0	1	24.6	26.6	0.92	465	503	0
1	53	(−)		P	1	0	1	1	1	0	0					1	1	1	1	34.9	28.5	1.23	718	585	26
1	41	(−)		P	1	0	1	1	1	0	0					0	0	1	0	34.3	31.1	1.10	1020	925	20
1	47	(−)		P	1	0	1	1	1	0	0					1	0	1	1	27.4	27.8	0.99	413	419	20
1	28	(−)		C	1	0	0	1	0	1	0					1	0	0	1	34.8	32.9	1.06	590	558	0
1	50	(−)		P	1	0	1	1	1	0	0					0	0	1	0	24.5	52.9	0.46	526	1137	0

Sex	Age	HCV-Ab	HCVRNA	status	既感染あ	vaccine	HBsAbscr	HBcAbscr	seroconve	chronic in	Indetermir	observation	TAF(M)	TDF(M)	3TC(M)	TAF(1/0)	TDF(1/0)	3TC(1/0)	FTC	CD4%	CD8%	CD4/8	CD4_count	CD8_count	HIV_RNA
1	31	(−)		P	1	0	1	1	1	0	0					1	0	0	1	19.3	64.0	0.30	298	988	13000
1	35	(−)		P	1	0	1	1	1	0	0					1	0	0	1	27.8	37.2	0.75	429	573	35
1	54	1.5	ケンシュツセス	P	1	0	1	1	1	0	0					0	0	1	0	11.6	53.8	0.22	127	588	39
1	51			P	1	0	1	1	1	0	0					1	0	0	1	15.4	52.8	0.29	367	1259	20
1	43	(−)		P	1	0	1	1	1	0	0					1	1	1	1	30.5	22.2	1.38	391	285	0
1	44			P	1	0	1	1	1	0	0					1	0	0	1	25.0	31.8	0.79	563	716	20
1	39	(−)	ケンシュツセス	P	1	0	1	1	1	0	0					1	0	0	1	29.8	16.3	1.82	586	321	0
1	43			P	1	0	1	1	1	0	0					1	1	0	1	29.2	31.3	0.93	486	521	20
1	44	(−)		P	1	0	1	1	1	0	0					1	0	0	1	19.8	56.8	0.35	513	1474	0
1	27		ケンシュツセス	P	1	0	1	1	1	0	0					1	0	0	1	33.9	32.0	1.06	737	697	0
1	33	(−)		P	1	0	1	1	1	0	0					1	0	0	1	19.5	56.9	0.34	464	1354	20
1	31			P	1	0	1	1	1	0	0					1	0	0	1	29.7	42.1	0.71	1060	1500	74
1	34	(−)		P	1	0	1	1	1	0	0					1	0	0	1	30.6	39.4	0.78	740	952	20
1	41	(−)		P	1	0	1	1	1	0	0					1	0	0	1	21.3	41.3	0.51	644	1253	0
1	49	(−)		P	1	0	1	1	1	0	0					1	0	0	1	15.4	43.8	0.35	642	1824	20
1	48	(−)		P	1	0	1	1	1	0	0					1	1	0	1	16.6	23.5	0.71	634	898	0
1	33	(−)		P	1	0	1	1	1	0	0					1	0	0	1	23.4	41.9	0.56	375	670	0
1	43		ケンシュツセス	P	1	0	1	1	1	0	0					1	1	0	1	22.5	27.2	0.83	520	630	0
1	39	(−)		C	1	0	0	1	0	1	0					1	0	0	1	28.9	42.1	0.69	638	929	26
1	31	(−)		P	1	0	1	1	1	0	0					1	1	0	1	7.0	51.5	0.14	85	623	150
1	45	(−)	ケンシュツセス	C	1	0	0	1	0	1	0					1	0	0	1	22.0	26.7	0.83	398	482	0
1	32	(−)		P	1	0	1	1	1	0	0					1	0	0	1	26.8	36.9	0.72	727	1003	0
1	58	(−)		P	1	0	1	1	1	0	0					1	0	0	1	17.1	56.3	0.30	107	352	0
1	43	(−)		P	1	0	1	1	1	0	0					1	0	0	1	24.8	41.3	0.60	715	1189	0
1	40	(−)		P	1	0	1	1	1	0	0					0	0	1	0	45.3	24.7	1.83	740	403	21
1	34	(−)		P	1	0	1	1	1	0	0					1	0	0	1	24.5	45.7	0.54	325	607	20
1	40	(−)		P	1	0	1	1	1	0	0					1	1	1	1	20.2	38.7	0.52	260	498	39
1	33	(−)		P	1	0	1	1	1	0	0					1	0	0	1	43.1	32.8	1.31	662	505	0
1	37	(−)	ケンシュツセス	P	1	0	1	1	1	0	0					1	0	0	1	43.3	29.5	1.47	491	334	20
1	40	(−)	ケンシュツセス	P	1	0	1	1	1	0	0					1	0	0	1	33.6	28.6	1.18	786	669	20
1	47	(−)		P	1	0	1	1	1	0						0	0	0	0	26.0	39.1	0.66	601	904	56
1	39	27.9	ケンシュツセス	P	1	0	1	1	1	0	0					1	0	0	1	37.7	37.5	1.01	832	828	0
1	40	(−)		C	1	0	0	1	0	1	0					1	0	0	1	35.4	48.5	0.73	1215	1663	0
1	31	(−)		P	1	0	1	1	1	0	0					1	0	0	1	29.2	39.7	0.74	617	838	20
1	36	(−)		P	1	0	1	1	1	0	0					1	0	0	1	21.3	49.8	0.43	373	872	0
1	36	(−)		P	1	0	1	1	1	0	0					1	0	1	1	37.7	38.6	0.98	515	528	0
1	50	(−)		P	1	0	1	1	1	0	0					1	0	0	1	26.6	21.2	1.26	583	465	0
1	33	62.4	ケンシュツセス	P	1	0	1	1	1	0	0					1	0	0	1	37.4	34.2	1.10	665	607	0
1	46	(−)		P	1	0	1	1	1	0	0					1	1	0	1	31.2	34.1	0.91	540	591	150
1	35	(−)		P	1	0	1	1	1	0	0					1	1	0	1	15.8	34.2	0.46	515	1116	0
1	27	(−)		P	1	0	1	1	1	0	0					1	0	1	1	27.3	33.4	0.82	581	710	20
1	40	(−)		C	1	0	0	1	0	1	0					1	0	0	1	32.2	33.0	0.98	409	419	0
1	34	(−)		P	1	0	1	1	1	0	0					1	0	0	1	28.9	27.7	1.04	490	471	0
1	50	(−)	ケンシュツセス	C	1	0	0	1	0	1	0					1	0	0	1	13.5	40.2	0.34	201	596	20
1	40	(−)		P	1	0	1	1	1	0	0					1	1	0	1	30.9	32.1	0.96	762	791	20
1	52	(−)		P	1	0	1	1	1	0	0					1	0	1	1	17.2	45.6	0.38	346	917	20
1	36	(−)		P	1	0	1	1	1	0	0					1	1	1	1	35.1	29.3	1.20	612	510	0
1	47	3.5	ケンシュツセス	P	1	0	1	1	1	0	0					0	0	1	0	41.8	35.6	1.17	1612	1374	20
1	64	(−)		P	1	0	1	1	1	0	0					1	0	0	1	22.0	34.5	0.64	530	832	20
1	28	(−)		P	1	0	1	1	1	0	0					1	0	0	1	27.9	33.4	0.83	544	653	0
1	47	19.2		P	1	0	1	1	1	0	0					1	0	0	1	14.2	58.4	0.24	592	2442	25
1	34	(−)		P	1	0	1	1	1	0	0					1	0	0	1	8.9	74.0	0.12	315	2616	660000
1	43	(−)		P	1	0	1	1	1	0	0					1	0	0	1	30.7	43.4	0.71	744	1053	0
1	44	(−)		P	1	0	1	1	1	0	0					1	0	0	1	26.4	26.6	0.99	450	454	20
1	43	(−)		P	1	0	1	1	1	0	0					1	0	0	1	24.2	29.5	0.82	361	440	0
1	57	(−)		P	1	0	1	1	1	0	0					1	0	0	1	8.3	44.3	0.19	58	309	0
1	28	(−)		P	1	0	1	1	1	0	0					1	0	0	1	33.0	24.4	1.35	493	365	47
1	41	(−)		P	1	0	1	1	1	0	0					1	0	0	1	41.8	23.5	1.78	892	501	20
1	36	(−)		P	1	0	1	1	1	0	0					1	0	0	1	23.1	38.2	0.60	481	795	0
1	46	15.6	ケンシュツセス	P	1	0	1	1	1	0	0					1	0	0	1	39.8	37.1	1.07	954	888	43
1	46	(−)		P	1	0	1	1	1	0	0					1	0	0	1	38.1	41.4	0.92	323	351	120

Sex	Age	HCV-Ab	HCVRNA	status	既感染あ	vaccine	HBsAbscr	HBcAbscr	seroconve	chronic in	Indetermir	observation	TAF(M)	TDF(M)	3TC(M)	TAF(1/0)	TDF(1/0)	3TC(1/0)	FTC	CD4%	CD8%	CD4/8	CD4_count	CD8_count	HIV_RNA
1	28	(−)		P	1	0	1	1	1	0	0					1	0	0	1	28.2	43.8	0.64	690	1072	0
1	36	(−)		P	1	0	1	1	1	0	0					1	0	0	1	22.3	41.9	0.53	476	893	20
1	42	(−)		P	1	0	1	1	1	0	0					1	1	0	1	26.7	30.3	0.88	574	653	20
1	31	(−)		P	1	0	1	1	1	0	0					1	0	0	1	31.1	36.2	0.86	487	566	0
1	31	(−)		P	1	0	1	1	1	0	0					1	0	0	1	37.0	38.0	0.97	725	746	0
1	28	(−)		P	1	0	1	1	1	0	0					0	0	1	0	33.6	33.0	1.02	961	943	22
1	39	(−)		C	1	0	0	1	0	1	0					1	0	0	1	30.1	40.6	0.74	654	882	20
2	39	(−)		P	1	0	1	1	1	0	0					1	0	0	1	27.5	27.8	0.99	298	301	0
1	34	(−)		P	1	0	1	1	1	0	0					1	0	0	1	22.2	44.7	0.50	319	642	0
2	44	(−)		P	1	0	1	1	1	0	0					1	1	1	1	29.9	43.8	0.68	422	619	98
1	44	(−)		P	1	0	1	1	1	0	0					0	0	1	0	32.4	34.8	0.93	794	853	0
1	41	(−)		P	1	0	1	1	1	0	0					1	1	0	1	32.6	38.6	0.84	495	586	0
1	29	(−)		P	1	0	1	1	1	0	0					1	0	0	1	1.5	64.0	0.02	12	523	270000
1	55	(−)		P	1	0	1	1	1	0	0					1	0	0	1	18.9	63.5	0.30	567	1904	170
1	27	(−)		C	1	0	0	1	0	1	0					1	0	0	1	33.6	35.6	0.94	518	549	0
1	41	(−)		P	1	0	1	1	1	0	0					1	0	0	1	13.0	43.9	0.30	454	1533	20
1	29	(−)		P	1	0	1	1	1	0	0					0	0	1	0	30.3	40.4	0.75	487	650	0
1	36	(−)		C	1	0	0	1	0	1	0					1	0	0	1	27.7	37.0	0.75	434	580	0
1	30	(−)		P	1	0	1	1	1	0	0					0	0	1	0	18.1	44.7	0.40	469	1158	130
1	38	(−)		P	1	0	1	1	1	0	0					1	0	0	1	33.8	24.5	1.38	751	543	0
1	28	(−)		C	1	0	0	1	0	1	0					1	0	0	1	14.3	41.9	0.34	290	848	23
1	56	(−)		P	1	0	1	1	1	0	0					1	0	0	1	10.6	74.3	0.14	117	819	39
1	37	(−)		P	1	0	1	1	1	0	0					1	1	0	1	31.2	36.8	0.85	787	927	0
1	28	(−)		P	1	0	1	1	1	0	0					1	0	0	1	19.1	44.7	0.43	222	519	0
1	55	(−)		P	1	0	1	1	1	0	0					0	0	1	0	31.8	33.6	0.95	836	884	20
1	36	(−)		P	1	0	1	1	1	0	0					1	1	0	1	32.8	36.5	0.90	830	927	20
1	26	(−)		C	1	0	0	1	0	1	0					1	0	0	1	44.4	36.7	1.21	753	622	20
1	27	(−)		P	1	0	1	1	1	0	0					1	0	0	1	26.2	37.5	0.70	433	619	250
1	25	(−)		C	1	0	0	1	0	1	0					1	0	0	1	39.8	30.3	1.31	599	456	0
1	36	(−)		P	1	0	0	1	0	0	1	6	0	0	74	0	0	1	0	15.3	24.7	0.62	480	778	0
1	67	(−)		P	1	0	0	1	0	0	1	14	76	94	0	1	1	0	1	35.1	27.3	1.29	631	491	0
1	60	(−)		P	1	0	0	1	0	0	1	0	0	156	142	0	1	1	1	24.5	42.1	0.58	988	1697	53
1	53	(−)		P	1	0	0	1	0	0	1	0	58	148	0	1	1	0	1	29.6	44.3	0.67	825	1232	33
1	57	(−)		P	1	0	1	0	0	0	1	0	48	0	107	1	0	1	1	33.6	41.7	0.81	548	681	20
1	59	(−)		P	1	0	0	1	0	0	1	0	27	0	0	1	0	0	1	21.4	59.0	0.36	327	904	0
1	74	(−)		P	1	0	0	1	0	0	1	0	0	0	333	0	0	1	0	37.2	21.4	1.74	499	287	0
1	82	(−)		P	1	0	1	0	0	0	1	0	0	53	202	0	1	1	1	25.5	22.5	1.13	448	395	0
1	71	(−)		P	1	0	0	1	0	0	1	0	0	0	204	0	0	1	0	30.0	37.0	0.81	715	881	0
2	56	(−)		P	1	0	1	0	0	0	1	0	68	91	145	1	1	1	1	35.0	19.3	1.81	1195	660	0
1	50	(−)		P	1	0	0	1	0	0	1	0	77	76	0	1	1	1	1	37.3	23.5	1.59	1448	912	37
1	60	(−)		P	1	0	0	1	0	0	1	0	57	0	167	1	0	1	1	23.0	25.3	0.91	412	453	0
1	59	15.1	ケンシュツセス	P	1	0	0	1	0	0	1	0	76	154	79	1	1	1	1	29.3	19.3	1.52	1054	695	0
1	72	(−)		P	1	0	0	0	0	0	1	0	19	35	198	1	1	1	1	25.9	26.3	0.98	641	651	26
1	73	(−)		P	1	0	0	1	0	0	1	0	76	146	128	1	1	1	1	25.5	45.0	0.57	466	824	20
2	56	55.0	ケンシュツセス	P	1	0	0	1	0	0	1	0	76	62	0	1	1	0	1	31.5	26.8	1.17	735	625	0
1	61	(−)		P	1	0	1	0	0	0	1	0	0	79	100	0	1	1	1	30.8	30.5	1.01	562	557	20
1	50	(−)		P	1	0	0	1	0	0	1	0	0	0	85	0	0	1	0	16.5	47.5	0.35	312	899	0
1	67	(−)		P	1	0	0	1	0	0	1	0	53	128	0	1	1	0	1	27.6	37.3	0.74	396	535	0
1	52	(−)		P	1	0	0	1	0	0	1	0	77	38	0	1	1	0	1	41.7	31.4	1.33	770	580	20
1	59	(−)		P	1	0	0	1	0	0	1	0	77	100	109	1	1	1	1	36.0	23.2	1.55	731	472	0
1	47	(−)		P	1	0	0	1	0	0	1	0	56	85	24	1	1	1	1	23.9	44.3	0.54	344	638	0
1	57	(−)	ケンシュツセス	P	1	0	0	1	0	0	1	0	76	214	180	1	1	1	1	31.5	31.0	1.01	454	448	0
1	54	(−)		P	1	0	0	1	0	0	1	0	75	153	172	1	1	1	1	12.6	54.7	0.23	141	612	20
1	64	(−)		P	1	0	0	1	0	0	1	0	0	0	285	0	0	1	0	44.1	24.8	1.78	515	289	0
1	50	(−)		P	1	0	1	0	0	0	1	0	74	94	0	1	1	0	1	29.0	54.3	0.53	673	1261	37
1	70	(−)		P	1	0	0	1	0	0	1	0	58	148	136	1	1	1	1	32.1	31.0	1.04	595	574	0
1	72	(−)		P	1	0	0	0	0	0	1	0	81	144	103	1	1	1	1	18.7	35.6	0.53	253	482	33
1	53	1.7	ケンシュツセス	P	1	0	1	0	0	0	1	0	77	108	81	1	1	1	1	33.5	37.0	0.91	688	760	0
1	60	(−)		P	1	0	0	1	0	0	1	0	76	49	130	1	1	1	1	19.7	38.2	0.52	504	978	0
1	47	(−)		P	1	0	0	1	0	0	1	0	0	6	107	0	1	1	1	3.9	73.9	0.05	78	1466	7500
1	45	(−)		P	1	0	1	0	0	0	1	0	37	0	0	1	0	0	1	16.9	52.7	0.32	414.5	1292.6	58

Sex	Age	HCV-Ab	HCVRNA	status	既感染あ	vaccine	HBsAbscr	HBcAbscr	seroconve	chronic in	Indetermi	observation	TAF(M)	TDF(M)	3TC(M)	TAF(1/0)	TDF(1/0)	3TC(1/0)	FTC	CD4%	CD8%	CD4/8	CD4_count	CD8_count	HIV_RNA
1	48	(−)		P	1	0	0	1	0	0	1	0	77	47	0	1	1	1	1	25.0	22.1	1.13	493	437	0
1	61	(−)		P	1	0	1	0	0	0	1	0	77	67	113	1	1	1	1	41.0	26.6	1.54	713	463	0
1	52	(−)		P	1	0	0	1	0	0	1	0	0	0	186	0	0	1	0	20.6	48.5	0.43	400	941	0
1	53	(−)		P	1	0	1	0	0	0	1	0	76	141	0	1	1	0	1	30.3	30.4	1.00	613	614	0
1	57	(−)		P	1	0	0	1	0	0	1	0	0	0	149	0	0	1	0	27.0	16.7	1.62	794	492	20
1	54	1.0	ケンシュツセズ	P	1	0	0	1	0	0	1	0	8	0	68	1	0	1	1	28.7	35.5	0.81	598	741	0
1	43	(−)		P	1	0	1	0	0	0	1	0	75	19	0	1	1	0	1	27.5	36.3	0.76	444	587	0
1	50	(−)		P	1	0	1	0	0	0	1	0	77	69	0	1	1	0	1	34.0	30.9	1.10	785	714	0
1	50	(−)		P	1	0	0	1	0	0	1	0	77	133	93	1	1	1	1	28.4	40.8	0.70	731	1052	0
1	40	(−)		P	1	0	0	1	0	0	1	0	79	106	0	1	1	0	1	21.5	35.0	0.61	483	788	20
1	51	(−)		P	1	0	0	1	0	0	1	0	75	73	0	1	1	0	1	14.5	33.3	0.43	614	1416	0
1	71	(−)		P	1	0	1	0	0	0	1	0	78	62	0	1	1	0	1	36.9	15.5	2.39	992	416	0
1	54	(−)		P	1	0	0	1	0	0	1	0	68	129	0	1	1	0	1	35.5	36.5	0.97	582	598	0
1	44	(−)		P	1	0	1	0	0	0	1	0	0	0	231	0	0	1	0	45.1	28.3	1.60	663	415	0
1	43	2.4	ケンシュツセズ	C	1	0	0	0	0	1	1	0	39	89	67	1	1	1	1	38.2	24.5	1.56	611	392	0
1	57	(−)		P	1	0	0	1	0	0	1	0	77	147	75	1	1	1	1	44.4	25.4	1.75	1427	816	20
1	55	(−)		C	1	0	0	0	0	1	1	0	82	149	120	1	1	1	1	23.8	18.7	1.27	466	367	23
1	57	14.3	ケンシュツセズ	P	1	0	0	1	0	0	1	0	51	171	211	1	1	1	1	46.7	17.4	2.69	1073	398	0
1	50	(−)		P	1	0	0	1	0	0	1	0	77	101	0	1	1	0	1	27.9	34.4	0.81	612	755	0
1	61	(−)		P	1	0	0	1	0	0	1	0	77	149	71	1	1	1	1	26.4	45.8	0.58	442	767	47
1	46	(−)	ケンシュツセズ	P	1	0	1	0	0	0	1	0	75	149	73	1	1	1	1	33.7	39.0	0.86	1096	1270	28
1	48	(−)		P	1	0	0	1	0	0	1	0	78	52	0	1	1	0	1	33.2	30.4	1.09	497	455	0
1	45	(−)	ケンシュツセズ	P	1	0	0	1	0	0	1	0	76	11	0	1	1	0	1	38.8	28.0	1.39	1019	735	0
1	47	(−)		P	1	0	1	0	0	0	1	0	78	5	0	1	1	0	1	34.4	34.9	0.99	720	730	20
1	52	(−)	ケンシュツセズ	P	1	0	0	1	0	0	1	0	56	144	0	1	1	0	1	24.8	32.9	0.75	560	742	56
1	63	(−)		P	1	0	0	1	0	0	1	0	68	150	40	1	1	1	1	28.6	33.6	0.85	785	921	20
1	53	(−)		P	1	0	0	1	0	0	1	0	84	35	0	1	1	0	1	26.9	43.5	0.62	713	1152	20
1	56	22.3	ケンシュツセズ	P	1	0	0	1	0	0	1	0	33	0	142	1	0	1	1	38.4	30.7	1.25	1249	998	0
1	48	(−)	ケンシュツセズ	P	1	0	0	1	0	0	1	0	74	94	0	1	1	0	1	14.2	36.2	0.39	343	874	0
1	45	(−)		P	1	0	0	0	0	0	1	0	0	0	172	0	0	1	0	37.8	32.7	1.16	795	688	0
1	57	(−)	ケンシュツセズ	P	1	0	1	0	0	0	1	0	72	75	0	1	1	0	1	29.7	41.0	0.72	555	767	0
1	75	(−)		P	1	0	0	0	0	0	1	0	0	0	217	0	0	1	0	31.4	35.0	0.90	508	566	0
1	61	(−)		P	1	0	0	1	0	0	1	0	59	26	18	1	1	1	1	35.9	18.9	1.90	468	247	0
1	47	(−)		P	1	0	0	0	0	0	1	0	81	54	0	1	1	0	1	21.9	53.1	0.41	318	772	0
1	49	(−)	ケンシュツセズ	P	1	0	0	1	0	0	1	0	78	62	0	1	1	0	1	17.8	62.8	0.28	265	937	0
1	54	(−)		P	1	0	0	1	0	0	1	0	12	19	183	1	1	1	1	22.5	27.6	0.81	673	826	0
1	44	(−)		P	1	0	0	1	0	0	1	0	78	132	0	1	1	0	1	29.7	26.3	1.13	559	495	0
1	47	(−)		P	1	0	0	1	0	0	1	0	83	1	126	1	1	1	1	45.5	23.2	1.96	1307	666	0
1	48	(−)	ケンシュツセズ	P	1	0	1	0	0	0	1	0	0	0	177	0	0	1	0	40.6	27.3	1.48	298	201	0
1	48	92.1	ケンシュツセズ	C	1	0	0	1	0	1	1	0	59	98	0	1	1	0	1	21.7	55.2	0.39	549	1390	20
1	55	(−)		P	1	0	0	1	0	0	1	0	76	86	0	1	1	0	1	37.1	29.2	1.27	732	576	20
1	54	(−)		P	1	0	1	0	0	0	1	0	75	78	0	1	1	0	1	43.7	17.3	2.53	1059	419	20
1	46	(−)		P	1	0	0	1	0	0	1	0	77	99	0	1	1	0	1	44.9	20.5	2.19	1045	476	20
1	50	(−)		P	1	0	0	1	0	0	1	0	0	76	90	0	1	1	1	26.7	29.6	0.90	752	831	0
1	50	(−)	ケンシュツセズ	P	1	0	0	1	0	0	1	0	24	73	64	1	1	1	1	53.6	15.1	3.56	2192	616	0
1	46	(−)		P	1	0	0	1	0	0	1	0	0	96	91	0	1	1	1	27.7	33.8	0.82	816	997	20
2	58	(−)		P	1	0	0	1	0	0	1	0	53	78	21	1	1	1	1	33.7	27.4	1.23	1027	836	29
1	49	(−)		P	1	0	0	1	0	0	1	0	77	5	0	1	1	0	1	22.1	42.0	0.53	470	891	0
1	47	(−)		P	1	0	0	1	0	0	1	0	78	99	0	1	1	0	1	35.0	21.8	1.60	1234	769	20
1	47	(−)		P	1	0	0	1	0	0	1	0	76	78	0	1	1	0	1	36.5	26.9	1.36	801	591	44
1	57			P	1	0	1	0	0	0	1	0	0	0	109	0	0	1	0	18.0	60.4	0.30	302	1014	26
1	42	(−)		P	1	0	1	0	0	0	1	0	76	66	0	1	1	0	1	31.3	28.3	1.11	678	611	20
1	48	21.8	5	P	1	0	0	0	0	0	1	0	76	20	0	1	1	0	1	36.0	41.3	0.87	870	1000	0
1	57	(−)		P	1	0	0	1	0	0	1	0	0	62	127	0	1	1	1	31.2	32.5	0.96	834	868	20
1	60	100<	ケンシュツセズ	P	1	0	0	1	0	0	1	0	77	88	0	1	1	0	1	35.6	28.0	1.27	1312	1030	0
1	56	(−)		P	1	0	0	1	0	0	1	0	78	108	0	1	1	0	1	35.9	25.6	1.40	792	564	20
1	50	(−)		P	1	0	0	1	0	0	1	0	78	110	0	1	1	0	1	26.3	22.4	1.17	303	259	0
1	59	(−)		P	1	0	0	1	0	0	1	0	82	103	0	1	1	0	1	26.6	25.1	1.06	554	521	0
1	76	(−)		P	1	0	0	1	0	0	1	0	22	0	147	1	0	1	1	26.7	39.1	0.68	588	860	0
1	80			P	1	0	0	0	0	0	1	0	41	0	0	1	0	0	1	19.7	37.0	0.53	362	682	0
1	69	(−)		P	1	0	0	1	0	0	1	0	43	22	123	1	1	1	1	33.8	26.7	1.27	835	659	20

Sex	Age	HCV-Ab	HCVRNA	status	既感染あ	vaccine	HBsAbscr	HBcAbscr	seroconve	chronic in	Indetermi	observation	TAF(M)	TDF(M)	3TC(M)	TAF(1/0)	TDF(1/0)	3TC(1/0)	FTC	CD4%	CD8%	CD4/8	CD4_count	CD8_count	HIV_RNA
1	58	(−)		P	1	0	0	1	0	0	1	0	0	0	136	0	0	1	0	31.8	22.6	1.41	788	560	0
1	41	23.8	7.3	P	1	0	0	1	0	0	1	0	76	98	0	1	1	0	1	35.6	42.1	0.85	529	626	26
1	44	(−)		P	1	0	0	1	0	0	1	0	48	0	124	1	0	1	1	46.5	23.0	2.03	974	481	150
1	60	(−)		P	1	0	0	1	0	0	1	0	77	74	0	1	1	0	1	26.5	43.9	0.60	706	1168	0
1	44	30.3	1.6	P	1	0	1	0	0	0	1	0	76	76	0	1	1	0	1	27.8	40.1	0.69	802	1156	0
1	48	(−)		P	1	0	1	0	0	0	1	0	77	68	0	1	1	0	1	29.9	26.7	1.12	649	580	20
1	52		ケンシュツセズ	P	1	0	0	1	0	0	1	0	72	79	14	1	1	1	1	32.9	39.9	0.82	448	543	20
1	55	(−)		P	1	0	1	0	0	0	1	0	75	86	0	1	1	0	1	34.2	30.4	1.12	731	651	0
1	34	(−)		P	1	0	0	1	0	0	1	0	81	47	0	1	1	0	1	28.4	37.8	0.75	542	721	0
1	46	(−)		P	1	0	0	1	0	0	1	0	76	91	98	1	1	1	1	36.8	23.4	1.57	1072	682	0
1	38	(−)		P	1	0	0	0	0	0	1	0	51	71	44	1	1	1	1	33.5	25.5	1.32	725	551	95
1	52	(−)		P	1	0	0	1	0	0	1	0	49	118	68	1	1	1	1	22.0	45.0	0.49	491	1006	20
1	48		ケンシュツセズ	P	1	0	1	0	0	0	1	0	75	70	0	1	1	0	1	36.4	20.2	1.80	625	346	20
1	63	(−)		P	1	0	0	0	0	0	1	0	43	14	95	1	1	1	1	19.8	41.8	0.47	609	1285	0
1	59	(−)		P	1	0	1	0	0	0	1	0	0	58	96	0	1	1	1	23.9	33.0	0.72	487	673	0
1	63	(−)		P	1	0	1	0	0	0	1	0	0	7	36	0	1	1	1	22.2	38.0	0.58	724	1239	0
1	76	(−)		P	1	0	0	1	0	0	1	0	0	3	159	0	1	1	1	17.0	21.3	0.80	427	533	0
1	47	(−)		P	1	0	0	1	0	0	1	0	17	9	133	1	1	1	1	33.9	37.4	0.91	878	967	0
1	59	11.1	ケンシュツセズ	P	1	0	0	1	0	0	1	0	77	83	0	1	1	0	1	26.4	45.8	0.58	598	1040	0
1	52	(−)		P	1	0	0	1	0	0	1	0	43	4	113	1	1	1	1	34.5	22.2	1.56	609	391	20
1	50	(−)	ケンシュツセズ	P	1	0	0	1	0	0	1	0	77	81	0	1	1	0	1	25.9	25.0	1.04	418	403	0
1	42	(−)	ケンシュツセズ	P	1	0	0	1	0	0	1	0	77	76	0	1	1	0	1	31.1	37.3	0.83	452	543	0
1	43	(−)		P	1	0	0	1	0	0	1	0	78	77	0	1	1	0	1	38.5	29.5	1.31	893	684	0
1	53	(−)		P	1	0	0	0	0	0	1	0	75	78	0	1	1	0	1	26.6	30.7	0.87	571	657	0
1	40	(−)		P	1	0	0	1	0	0	1	0	78	43	0	1	1	0	1	34.8	31.7	1.10	1023	931	0
1	55	(−)		P	1	0	0	0	0	0	1	0	78	77	0	1	1	0	1	40.4	19.9	2.03	710	350	0
1	50	2.1	ケンシュツセズ	P	1	0	1	0	0	0	1	0	50	66	0	1	1	0	1	11.2	37.8	0.30	307	1038	0
1	33	(−)		P	1	0	0	0	0	0	1	0	75	69	0	1	1	0	1	44.4	35.3	1.26	1383	1100	0
1	58	(−)		P	1	0	0	1	0	0	1	0	43	0	109	1	0	1	1	28.6	43.3	0.66	443	671	0
1	38	(−)		P	1	0	0	0	0	0	1	0	65	29	1	1	1	1	1	29.0	28.3	1.02	554	541	20
1	42	(−)		P	1	0	0	1	0	0	1	0	77	69	0	1	1	0	1	35.7	33.5	1.07	923	866	0
1	44	(−)		P	1	0	0	1	0	0	1	0	81	32	0	1	1	0	1	32.5	24.0	1.35	766	566	20
1	51	(−)		P	1	0	0	1	0	0	1	0	51	21	78	1	1	1	1	34.5	21.7	1.60	615	386	0
1	55	(−)	ケンシュツセズ	P	1	0	0	1	0	0	1	0	54	83	0	1	1	0	1	14.6	54.7	0.27	352	1312	61
1	42	(−)		P	1	0	0	1	0	0	1	0	78	69	0	1	1	0	1	21.7	50.6	0.43	699	1633	23
1	39	(−)		P	1	0	0	0	0	0	1	0	77	69	0	1	1	0	1	32.8	42.2	0.78	733	942	0
1	42	(−)		P	1	0	0	0	0	0	1	0	16	0	0	1	0	0	1	22.8	60.1	0.38	415	1091	0
1	60	(−)		P	1	0	1	0	0	0	1	0	47	42	55	1	1	1	1	38.4	27.4	1.40	448	320	0
1	45	15.2	ケンシュツセズ	P	1	0	0	1	0	0	1	0	76	138	44	1	1	1	1	29.0	24.8	1.17	524	448	0
1	58	(−)		P	1	0	0	0	0	0	1	0	5	53	75	1	1	1	1	38.6	18.9	2.04	884	434	20
1	43	(−)		P	1	0	1	0	0	0	1	0	55	62	23	1	1	1	1	34.2	33.1	1.03	919	890	20
1	37	(−)		P	1	0	0	0	0	0	1	0	77	48	0	1	1	0	1	35.7	37.5	0.95	1130	1187	0
1	50	(−)		P	1	0	1	0	0	0	1	0	75	65	0	1	1	0	1	23.4	42.8	0.55	356	650	0
1	42	(−)		P	1	0	0	1	0	0	1	0	76	59	0	1	1	0	1	29.0	35.2	0.82	400	487	0
1	46	(−)		P	1	0	0	1	0	0	1	0	54	0	0	1	0	0	1	22.4	34.2	0.65	446	683	0
1	61	(−)		P	1	0	1	0	0	0	1	0	74	54	0	1	1	0	1	44.6	26.4	1.69	724	428	0
1	40	(−)		P	1	0	0	1	0	0	1	0	77	52	0	1	1	0	1	32.1	40.0	0.80	626	779	0
2	44	(−)		P	1	0	0	0	0	0	1	0	73	54	0	1	1	0	1	35.3	37.6	0.94	488	519	20
1	36	(−)		P	1	0	0	0	0	0	1	0	58	62	0	1	1	0	1	25.3	39.8	0.64	569	894	0
1	52	(−)		P	1	0	0	1	0	0	1	0	0	0	126	0	1	1	1	26.6	31.4	0.85	294	347	20
1	47	(−)	ケンシュツセズ	P	1	0	0	0	0	0	1	0	62	82	0	1	1	0	1	23.1	34.4	0.67	497	741	0
1	76	1.1		P	1	0	0	1	0	0	1	0	77	46	0	1	1	0	1	37.7	32.4	1.16	444	382	0
1	44		ケンシュツセズ	P	1	0	0	1	0	0	1	0	77	38	0	1	1	0	1	39.3	35.5	1.11	701	633	20
1	50	(−)		P	1	0	0	1	0	0	1	0	45	44	32	1	1	1	1	28.1	28.7	0.98	551	564	0
1	51	(−)		P	1	0	0	0	0	0	1	0	76	43	0	1	1	0	1	25.3	31.8	0.80	320	402	20
1	38	(−)		P	1	0	0	1	0	0	1	0	76	44	0	1	1	0	1	27.3	36.8	0.74	462	623	0
1	34	74.0	ケンシュツセズ	P	1	0	1	0	0	0	1	0	47	0	70	1	0	1	1	28.3	42.8	0.66	518	782	0
1	38	(−)		P	1	0	0	1	0	0	1	0	82	20	0	1	1	0	1	47.1	25.7	1.83	955	522	0
1	56	(−)		P	1	0	0	1	0	0	1	0	0	0	76	0	0	1	0	34.6	19.5	1.78	807	454	0
1	52	(−)		P	1	0	1	0	0	0	1	0	55	39	21	1	1	1	1	49.4	22.0	2.25	683	304	0
1	48	(−)		P	1	0	0	1	0	0	1	0	0	4	100	0	1	1	1	18.4	47.2	0.39	499	1277	0

Sex	Age	HCV-Ab	HCVRNA	status	既感染あ	vaccine	HBsAbscr	HBcAbscr	seroconve	chronic in	Indetermi	observation	TAF(M)	TDF(M)	3TC(M)	TAF(1/0)	TDF(1/0)	3TC(1/0)	FTC	CD4%	CD8%	CD4/8	CD4_count	CD8_count	HIV_RNA
1	35			P	1	0	0	1	0	0	1	0	0	0	81	0	0	1	0	39.8	34.8	1.14	886	775	0
1	34	(−)		P	1	0	0	1	0	0	1	0	78	77	0	1	1	0	1	31.1	32.0	0.97	605	624	0
1	34	(−)		P	1	0	1	0	0	0	1	0	0	0	90	0	0	1	0	29.1	44.5	0.65	588	899	0
1	57	(−)		P	1	0	0	1	0	0	1	0	78	8	0	1	1	0	1	31.1	28.0	1.11	511	460	0
1	45	(−)		P	1	0	0	1	0	0	1	0	78	12	0	1	1	0	1	19.2	27.6	0.70	303	436	0
1	36	2.6	ケンシュツセス	P	1	0	0	1	0	0	1	0	78	12	0	1	1	0	1	14.2	36.1	0.39	163	414	20
1	40	(−)		P	1	0	0	1	0	0	1	0	78	67	33	1	1	1	1	33.7	31.2	1.08	873	809	22
1	43	(−)		P	1	0	0	1	0	0	1	0	58	0	0	1	0	0	1	37.1	36.3	1.02	924	904	0
1	49	(−)		P	1	0	0	1	0	0	1	0	51	0	28	1	0	1	1	27.3	39.7	0.69	416	606	0
1	51	(−)		P	1	0	1	0	0	0	1	0	76	73	0	1	1	0	1	47.0	21.5	2.18	984	451	0
1	54	(−)		P	1	0	0	1	0	0	1	0	59	0	0	1	0	0	1	24.1	44.6	0.54	463	857	62
1	51	(−)		P	1	0	0	1	0	0	1	0	49	0	18	1	0	1	1	21.9	42.5	0.52	374	727	0
1	49	(−)		P	1	0	0	1	0	0	1	0	0	17	108	0	1	1	1	21.3	52.1	0.41	487	1191	20
1	73	(−)		P	1	0	0	1	0	0	1	0	59	0	0	1	0	0	1	23.5	45.0	0.52	745	1430	0
1	53	(−)		P	1	0	0	1	0	0	1	0	48	0	0	1	0	0	1	9.8	64.6	0.15	135	888	800
1	32	(−)		P	1	0	0	1	0	0	1	0	48	0	22	1	0	1	1	36.1	39.8	0.91	708	781	20
1	41	(−)		P	1	0	0	1	0	0	1	0	28	0	0	1	0	0	1	16.5	38.1	0.43	404	931	32
1	28	(−)	ケンシュツセス	P	1	0	0	1	0	0	1	0	41	0	0	1	0	0	1	28.9	31.6	0.91	555	608	26
1	38	71.9	ケンシュツセス	P	1	0	0	1	0	0	1	0	24	0	0	1	0	0	1	16.6	40.6	0.41	398	970	22
1	40	(−)		P	1	0	0	1	0	0	1	0	17	2	0	1	1	0	1	20.4	39.3	0.52	402	774	40
1	34	(−)		P	1	0	0	1	0	0	1	0	5	0	0	1	0	0	1	11.1	34.0	0.33	128	391	150
1	31	(−)		P	1	0	0	1	0	0	1	0	26	25	0	1	1	0	1	15.5	47.5	0.33	354	1087	0
1	43	(−)		P	1	0	0	1	0	0	1	0	7	0	0	1	0	0	1	21.9	56.8	0.39	744	1929	20
1	60	(−)		P	1	0	1	0	0	0	1	0	6	0	0	1	0	0	1	5.3	58.6	0.09	111	1222	20
1	73	(−)		N	0	0	0	0	0	0	0					0	0	1	0						
2	47			N	0	0	0	0	0	0						0	0	0	0						
1	33	(−)		N	0	0	0	0	0	0						0	0	0	0						
1	21	(−)		N	0	0	0	0	0	0						0	0	0	0						
1	47	(−)		N	0	0	0	0	0	0						0	0	0	0						
1	56	(−)		N	0	0	0	0	0	0	0					1	1	1	1						
1	78	(−)	ケンシュツセス	N	0	0	0	0	0	0	0					1	1	0	1						
1	36	(−)		N	0	0	0	0	0	0	0					1	0	0	1						
1	61	(−)		N	0	0	0	0	0	0	0					1	1	0	1						
2	90	(−)		N	0	0	0	0	0	0	0					0	1	1	1						
2	81	(−)		N	0	0	0	0	0	0	0					1	1	1	1						
1	61	(−)		N	0	0	0	0	0	0	0					1	0	0	1						
1	37	(−)		N	0	0	0	0	0	0	0					1	1	0	1						
1	51	(−)		N	0	0	0	0	0	0	0					1	0	0	1						
1	51	(−)		N	0	0	0	0	0	0	0					1	0	1	1						
1	60	(−)		N	0	0	0	0	0	0	0					0	1	1	1						
1	64	(−)		N	0	0	0	0	0	0	0					1	1	1	1						
1	42	(−)		N	0	0	0	0	0	0	0					1	1	0	1						
1	56	(−)	ケンシュツセス	N	0	0	0	0	0	0	0					0	0	1	0						
1	48	(−)		N	0	0	0	0	0	0	0					0	0	1	0						
1	43	(−)		N	0	0	0	0	0	0	0					1	0	0	1						
1	57	(−)	0.5>	N	0	0	0	0	0	0	0					1	1	0	1						
1	52	(−)		N	0	0	0	0	0	0	0					1	1	1	1						
1	66	(−)		N	0	0	0	0	0	0	0					1	1	1	1						
1	57	(−)		N	0	0	0	0	0	0	0					1	1	1	1						
1	57	(−)		N	0	0	0	0	0	0	0					1	1	1	1						
1	48	(−)		N	0	0	0	0	0	0	0					1	0	1	1						
1	52	(−)	ケンシュツセス	N	0	0	0	0	0	0	0					1	1	0	1						
2	73	(−)		N	0	0	0	0	0	0	0					1	1	1	1						
2	60	(−)		N	0	0	0	0	0	0	0					1	0	1	1						
1	55	(−)		N	0	0	0	0	0	0	0					0	0	1	0						
2	64	(−)	ケンシュツセス	N	0	0	0	0	0	0	0					0	1	0	1						
2	50	(−)		N	0	0	0	0	0	0	0					0	1	1	1						
1	54	(−)		N	0	0	0	0	0	0	0					0	1	1	1						
1	56	(−)		N	0	0	0	0	0	0	0					1	0	1	1						
1	45	(−)		N	0	0	0	0	0	0	0					1	0	0	1						
1	50	(−)		N	0	0	0	0	0	0	0					1	1	1	1						

Sex	Age	HCV-Ab	HCVRNA	status	既感染あ	vaccine	HBsAbscr	HBcAbscr	seroconve	chronic in	Indetermi	observation	TAF(M)	TDF(M)	3TC(M)	TAF(1/0)	TDF(1/0)	3TC(1/0)	FTC	CD4%	CD8%	CD4/8	CD4_coun	CD8_coun	HIV_RNA
2	52	25.5	ケンシュツセス	N	0	0	0	0	0	0	0					1	1	1	1						
1	64	(−)		N	0	0	0	0	0	0	0					0	1	1	1						
1	47	(−)		N	0	0	0	0	0	0	0					1	1	1	1						
2	50	(−)		N	0	0	0	0	0	0	0					1	1	1	1						
1	56	(−)		N	0	0	0	0	0	0	0					1	1	0	1						
1	55	(−)		N	0	0	0	0	0	0	0					1	1	1	1						
1	49	(−)		N	0	0	0	0	0	0	0					0	0	1	0						
1	53	(−)		N	0	0	0	0	0	0	0					0	1	1	1						
1	72	(−)		N	0	0	0	0	0	0	0					1	1	0	1						
2	62	(−)		N	0	0	0	0	0	0	0					0	1	1	1						
1	45	(−)		N	0	0	0	0	0	0	0					1	1	0	1						
1	50	(−)		N	0	0	0	0	0	0	0					1	1	0	1						
1	45	(−)		N	0	0	0	0	0	0	0					0	0	1	0						
1	58	(−)		N	0	0	0	0	0	0	0					1	1	1	1						
1	48	(−)		N	0	0	0	0	0	0	0					1	1	1	1						
1	63	(−)		N	0	0	0	0	0	0	0					1	1	0	1						
1	41	(−)		N	0	0	0	0	0	0	0					1	0	0	1						
1	57	(−)		N	0	0	0	0	0	0	0					1	1	0	1						
1	53	(−)		N	0	0	0	0	0	0	0					1	1	0	1						
1	43	(−)		N	0	0	0	0	0	0	0					1	1	0	1						
2	62			N	0	0	0	0	0	0	0					0	0	1	0						
1	48	(−)		N	0	0	0	0	0	0	0					0	0	1	0						
1	38	27.7	ケンシュツセス	N	0	0	0	0	0	0	0					1	1	0	1						
1	64	(−)		N	0	0	0	0	0	0	0					0	0	1	0						
1	66			N	0	0	0	0	0	0	0					0	1	1	1						
1	62	(−)		N	0	0	0	0	0	0	0					1	0	1	1						
1	45	(−)		N	0	0	0	0	0	0	0					1	1	0	1						
1	43	(−)		N	0	0	0	0	0	0	0					1	1	0	1						
1	39	(−)		N	0	0	0	0	0	0	0					1	1	1	1						
1	58	(−)		N	0	0	0	0	0	0	0					1	1	0	1						
1	55	(−)		N	0	0	0	0	0	0	0					1	1	0	1						
1	48			N	0	0	0	0	0	0	0					1	1	0	1						
1	53			N	0	0	0	0	0	0	0					0	0	1	0						
1	72	(−)		N	0	0	0	0	0	0	0					0	0	1	0						
1	54	(−)		N	0	0	0	0	0	0	0					1	1	0	1						
2	45	(−)		N	0	0	0	0	0	0	0					1	0	1	1						
1	43	(−)		N	0	0	0	0	0	0	0					1	1	0	1						
1	44	(−)		N	0	0	0	0	0	0	0					0	0	1	0						
1	41	(−)		N	0	0	0	0	0	0	0					1	1	0	1						
1	39	(−)		N	0	0	0	0	0	0	0					1	1	0	1						
1	48	(−)		N	0	0	0	0	0	0	0					1	1	0	1						
1	68	(−)		N	0	0	0	0	0	0	0					1	1	0	1						
1	33	(−)		N	0	0	0	0	0	0	0					1	1	0	1						
1	33			N	0	0	0	0	0	0	0					1	1	0	1						
1	44	(−)		N	0	0	0	0	0	0	0					1	1	0	1						
1	48	(−)		N	0	0	0	0	0	0	0					1	0	1	1						
1	38	(−)		N	0	0	0	0	0	0	0					1	1	0	1						
1	38	(−)		N	0	0	0	0	0	0	0					1	0	1	1						
1	39	(−)		N	0	0	0	0	0	0	0					1	1	0	1						
1	48	(−)		N	0	0	0	0	0	0	0					1	1	1	1						
1	34	(−)		N	0	0	0	0	0	0	0					1	1	0	1						
1	40	(−)		N	0	0	0	0	0	0	0					1	1	0	1						
1	46	(−)		N	0	0	0	0	0	0	0					1	1	0	1						
1	50	(−)		N	0	0	0	0	0	0	0					1	0	1	1						
1	69	(−)		N	0	0	0	0	0	0	0					1	1	1	1						
1	54			N	0	0	0	0	0	0	0					1	1	0	1						
1	43	(−)		N	0	0	0	0	0	0	0					1	1	0	1						
1	53	(−)		N	0	0	0	0	0	0	0					1	1	0	1						
1	42	(−)		N	0	0	0	0	0	0	0					1	1	0	1						
1	34	84.0	ケンシュツセス	N	0	0	0	0	0	0	0					1	1	0	1						
1	49	(−)		N	0	0	0	0	0	0	0					1	1	0	1						

Sex	Age	HCV-Ab	HCVRNA	status	既感染あ	vaccine	HBsAbscr	HBcAbscr	seroconve	chronic in	Indetermi	observation	TAF(M)	TDF(M)	3TC(M)	TAF(1/0)	TDF(1/0)	3TC(1/0)	FTC	CD4%	CD8%	CD4/8	CD4_count	CD8_count	HIV_RNA
1	36	(−)		N	0	0	0	0	0	0	0					1	0	0	1						
1	36	(−)		N	0	0	0	0	0	0	0					1	0	0	1						
1	32	(−)		N	0	0	0	0	0	0	0					1	1	0	1						
1	48	(−)		N	0	0	0	0	0	0	0					0	1	1	1						
1	34	(−)		N	0	0	0	0	0	0	0					1	0	0	1						
1	43	(−)		N	0	0	0	0	0	0	0					0	1	1	1						
1	43	(−)		N	0	0	0	0	0	0	0					1	1	1	1						
1	38			N	0	0	0	0	0	0	0					1	0	0	1						
1	52	(−)		N	0	0	0	0	0	0	0					1	1	0	1						
1	53	(−)		N	0	0	0	0	0	0	0					1	1	0	1						
1	31	(−)		N	0	0	0	0	0	0	0					1	1	0	1						
1	34	(−)		N	0	0	0	0	0	0	0					1	0	0	1						
2	70	(−)		N	0	0	0	0	0	0	0					1	0	1	1						
1	41	(−)		N	0	0	0	0	0	0	0					1	1	0	1						
1	38	(−)		N	0	0	0	0	0	0	0					1	0	1	1						
1	50	(−)		N	0	0	0	0	0	0	0					0	0	1	0						
2	57	(−)		N	0	0	0	0	0	0	0					1	0	1	1						
1	46	(−)		N	0	0	0	0	0	0	0					0	0	1	0						
1	52	(−)		N	0	0	0	0	0	0	0					1	0	1	1						
2	52	(−)		N	0	0	0	0	0	0	0					0	0	1	0						
1	43			N	0	0	0	0	0	0	0					0	0	1	0						
1	48	53.5	ｹﾝｼｭﾂｾｽﾞ	N	0	0	0	0	0	0	0					1	0	0	1						
1	48		ｹﾝｼｭﾂｾｽﾞ	N	0	0	0	0	0	0	0					0	0	1	0						
1	28			N	0	0	0	0	0	0	0					1	0	0	1						
1	35	(−)		N	0	0	0	0	0	0	0					1	1	0	1						
1	33	(−)		N	0	0	0	0	0	0	0					0	0	1	0						
1	37	(−)		N	0	0	0	0	0	0	0					0	0	1	0						
1	54			N	0	0	0	0	0	0	0					1	1	0	1						
1	32	18.0	7.1	N	0	0	0	0	0	0	0					0	0	1	0						
1	53	(−)		N	0	0	0	0	0	0	0					0	0	1	0						
1	51	(−)		N	0	0	0	0	0	0	0					0	1	1	1						
1	46	(−)		N	0	0	0	0	0	0	0					1	0	0	1						
1	35	(−)		N	0	0	0	0	0	0	0					0	0	1	0						
1	25	(−)		N	0	0	0	0	0	0	0					1	0	0	1						
1	34	(−)		N	0	0	0	0	0	0	0					0	0	1	0						
1	34	(−)		N	0	0	0	0	0	0	0					1	1	0	1						
1	31	(−)		N	0	0	0	0	0	0	0					1	1	1	1						
2	39	(−)		N	0	0	0	0	0	0	0					0	0	1	0						
1	30	(−)		N	0	0	0	0	0	0	0					1	0	1	1						
1	38	(−)		N	0	0	0	0	0	0	0					1	0	0	1						
1	29	(−)		N	0	0	0	0	0	0	0					1	0	1	1						
1	39	(−)		N	0	0	0	0	0	0	0					1	0	0	1						
2	50			N	0	0	0	0	0	0	0					0	0	1	0						
1	29	(−)		N	0	0	0	0	0	0	0					1	0	0	1						
1	32			N	0	0	0	0	0	0	0					1	0	1	1						
1	37	(−)		N	0	0	0	0	0	0	0					1	0	0	1						
1	32	72.0	ｹﾝｼｭﾂｾｽﾞ	N	0	0	0	0	0	0	0					1	0	0	1						
1	39	(−)		N	0	0	0	0	0	0	0					1	0	0	1						
1	66	(−)		N	0	0	0	0	0	0	0					0	1	1	1						
1	42	(−)		N	0	0	0	0	0	0	0					0	0	1	0						
1	37	(−)		N	0	0	0	0	0	0	0					1	0	0	1						
1	49	(−)		N	0	0	0	0	0	0	0					0	0	1	0						
1	50	(−)		N	0	0	0	0	0	0	0					0	0	1	0						
1	32	(−)		N	0	0	0	0	0	0	0					1	0	1	1						
1	62	(−)		N	0	0	0	0	0	0	0					1	1	1	1						
1	53	(−)		N	0	0	0	0	0	0	0					0	0	1	0						
1	49	3.4	ｹﾝｼｭﾂｾｽﾞ	N	0	0	0	0	0	0	0					1	1	0	1						
1	34	(−)		N	0	0	0	0	0	0	0					1	0	0	1						
1	32	(−)		N	0	0	0	0	0	0	0					1	0	0	1						
1	42	(−)		N	0	0	0	0	0	0	0					0	0	1	0						
2	34			N	0	0	0	0	0	0	0					1	1	0	1						

Sex	Age	HCV-Ab	HCVRNA	status	既感染あ	vaccine	HBsAbscr	HBcAbscr	seroconve	chronic in	Indetermir	observation	TAF(M)	TDF(M)	3TC(M)	TAF(1/0)	TDF(1/0)	3TC(1/0)	FTC	CD4%	CD8%	CD4/8	CD4_count	CD8_count	HIV_RNA
1	44	(−)		N	0	0	0	0	0	0	0					1	0	0	1						
1	32	(−)		N	0	0	0	0	0	0	0					1	0	0	1						
1	66	(−)		N	0	0	0	0	0	0	0					0	0	1	0						
1	53	(−)		N	0	0	0	0	0	0	0					1	0	0	1						
1	35	(−)		N	0	0	0	0	0	0	0					0	0	1	0						
1	29	(−)		N	0	0	0	0	0	0	0					1	0	0	1						
1	46	(−)		N	0	0	0	0	0	0	0					1	0	0	1						
1	33	(−)		N	0	0	0	0	0	0	0					1	0	0	1						
1	51	39.4	ｹﾝｼｭﾂｾｽﾞ	N	0	0	0	0	0	0	0					0	0	1	0						
1	58	(−)		N	0	0	0	0	0	0	0					1	0	0	1						
1	44	(−)		N	0	0	0	0	0	0	0					0	0	1	0						
1	25	(−)		N	0	0	0	0	0	0	0					1	0	0	1						
1	25	(−)		N	0	0	0	0	0	0	0					1	0	0	1						
1	30	(−)		N	0	0	0	0	0	0	0					1	1	0	1						
1	37	69.6	6.1	N	0	0	0	0	0	0	0					1	1	0	1						
1	49	(−)		N	0	0	0	0	0	0	0					1	1	1	1						
1	71	(−)		N	0	0	0	0	0	0	0					1	0	0	1						
1	51	(−)		N	0	0	0	0	0	0	0					1	0	0	1						
1	44	(−)		N	0	0	0	0	0	0	0					1	0	0	1						
1	32	(−)		N	0	0	0	0	0	0	0					1	1	0	1						
1	59			N	0	0	0	0	0	0	0					1	0	0	1						
1	32	(−)		N	0	0	0	0	0	0	0					1	0	0	1						
1	40	(−)		N	0	0	0	0	0	0	0					1	0	0	1						
1	40	(−)		N	0	0	0	0	0	0	0					1	0	0	1						
1	25	(−)		N	0	0	0	0	0	0	0					1	0	0	1						
1	33	(−)		N	0	0	0	0	0	0	0					1	0	0	1						
1	29	(−)		N	0	0	0	0	0	0	0					1	0	0	1						
1	40	(−)		N	0	0	0	0	0	0	0					1	0	0	1						
1	42	(−)		N	0	0	0	0	0	0	0					1	0	0	1						
1	24	(−)		N	0	0	0	0	0	0	0					1	0	0	1						
1	28	(−)		N	0	0	0	0	0	0	0					1	0	0	1						
1	28	(−)		N	0	0	0	0	0	0	0					1	0	0	1						
1	27	(−)		N	0	0	0	0	0	0	0					1	0	0	1						
1	30	(−)		N	0	0	0	0	0	0	0					1	0	0	1						
1	28	(−)		N	0	0	0	0	0	0	0					1	1	1	1						
1	31	(−)		N	0	0	0	0	0	0	0					1	0	0	1						
1	30	(−)		N	0	0	0	0	0	0	0					1	0	0	1						
1	41	(−)		N	0	0	0	0	0	0	0					0	0	1	0						
1	49	(−)		N	0	0	0	0	0	0	0					1	1	0	1						
1	36	(−)		N	0	0	0	0	0	0	0					1	0	0	1						
1	26	(−)		N	0	0	0	0	0	0	0					1	0	0	1						
1	27	(−)		N	0	0	0	0	0	0	0					1	0	0	1						
1	65	(−)		N	0	0	0	0	0	0	0					1	1	0	1						
1	31	(−)		N	0	0	0	0	0	0	0					1	0	0	1						
1	44	(−)		N	0	0	0	0	0	0	0					1	0	0	1						
1	37	(−)		N	0	0	0	0	0	0	0					1	0	0	1						
1	33	(−)		N	0	0	0	0	0	0	0					1	0	0	1						
1	28	(−)		N	0	0	0	0	0	0	0					1	0	0	1						
1	34	(−)		N	0	0	0	0	0	0	0					1	0	0	1						
1	43	(−)		N	0	0	0	0	0	0	0					0	0	1	0						
1	36	(−)		N	0	0	0	0	0	0	0					1	0	0	1						
1	33	(−)		N	0	0	0	0	0	0	0					1	0	0	1						
1	32	17.7	6.5	N	0	0	0	0	0	0	0					1	0	0	1						
1	29	(−)		N	0	0	0	0	0	0	0					1	0	0	1						
1	36	(−)		N	0	0	0	0	0	0	0					1	0	0	1						
1	42	(−)		N	0	0	0	0	0	0	0					1	0	0	1						
1	34	(−)		N	0	0	0	0	0	0	0					1	0	0	1						
1	48	(−)		N	0	0	0	0	0	0	0					1	0	0	1						
1	59	(−)		N	0	0	0	0	0	0	0					1	0	0	1						
1	29	(−)		N	0	0	0	0	0	0	0					1	0	0	1						
1	31	(−)		N	0	0	0	0	0	0	0					1	0	0	1						

Sex	Age	HCV-Ab	HCVRNA	status	既感染あ	vaccine	HBsAbscr	HBcAbscr	seroconve	chronic in	Indetermi	observation	TAF(M)	TDF(M)	3TC(M)	TAF(1/0)	TDF(1/0)	3TC(1/0)	FTC	CD4%	CD8%	CD4/8	CD4_count	CD8_count	HIV_RNA
1	30	(−)		N	0	0	0	0	0	0	0					1	0	0	1						
1	29	(−)		N	0	0	0	0	0	0	0					1	0	0	1						
1	29	(−)		N	0	0	0	0	0	0	0					1	0	0	1						
1	34	(−)		N	0	0	0	0	0	0	0					1	0	0	1						
1	28	(−)		N	0	0	0	0	0	0	0					1	0	0	1						
1	44	(−)		N	0	0	0	0	0	0	0					1	0	0	1						
1	79	(−)		N	0	0	0	0	0	0	0					1	0	0	1						
1	32	(−)		N	0	0	0	0	0	0	0					1	0	0	1						
1	23	(−)		N	0	0	0	0	0	0	0					1	0	0	1						
1	28	(−)		N	0	0	0	0	0	0	0					1	0	0	1						
1	47	(−)		N	0	0	0	0	0	0	0					0	1	1	1						
1	31	(−)		N	0	0	0	0	0	0	0					1	0	0	1						
1	51	(−)		N	0	0	0	0	0	0	0					1	1	0	1						
1	25	(−)		N	0	0	0	0	0	0	0					1	0	0	1						
1	38	(−)		N	0	0	0	0	0	0	0					1	0	0	1						
1	31	(−)		N	0	0	0	0	0	0	0					1	0	0	1						
1	51	(−)		N	0	0	0	0	0	0	0					1	1	0	1						
1	22	(−)		N	0	0	0	0	0	0	0					1	0	0	1						
1	33	(−)		N	0	0	0	0	0	0	0					1	0	0	1						
1	38	(−)		N	0	0	0	0	0	0	0					1	0	0	1						
1	48	(−)		N	0	0	0	0	0	0	0					1	1	0	1						
1	31	(−)		N	0	0	0	0	0	0	0					1	0	0	1						
1	25	(−)		N	0	0	0	0	0	0	0					1	0	0	1						
1	46	(−)		N	0	0	0	0	0	0	0					1	0	0	1						
1	28	(−)		N	0	0	0	0	0	0	0					1	0	0	1						
1	27	(−)		N	0	0	0	0	0	0	0					1	0	0	1						
1	38	(−)		N	0	0	0	0	0	0	0					1	1	1	1						
1	56	(−)		N	0	0	0	0	0	0	0					0	1	1	1						
1	27	(−)		N	0	0	0	0	0	0	0					1	0	0	1						
1	27	(−)		N	0	0	0	0	0	0	0					1	0	0	1						
1	54	(−)	ケンシュツセス	W	0	1	#N/A	0	#N/A	0	0					1	0	1	1						
1	40	(−)		W	0	1	#N/A	0	#N/A	0	0					1	1	1	1						
1	44	(−)	ケンシュツセス	W	0	1	#N/A	0	#N/A	0	0					1	1	1	1						
1	60	(−)		W	0	1	#N/A	0	#N/A	0	0					1	1	1	1						
1	50	(−)		W	0	1	#N/A	0	#N/A	0	0					1	1	0	1						
1	65	(−)		W	0	1	#N/A	0	#N/A	0	0					0	1	1	1						
1	44	(−)		W	0	1	#N/A	0	#N/A	0	0					1	1	0	1						
1	47	(−)		W	0	1	#N/A	0	#N/A	0	0					0	0	1	0						
1	53	(−)	ケンシュツセス	W	0	1	#N/A	0	#N/A	0	0					1	1	1	1						
2	48	(−)		W	0	1	#N/A	0	#N/A	0	0					0	0	1	0						
1	67	(−)	ケンシュツセス	W	0	1	#N/A	0	#N/A	0	0					1	1	1	1						
1	57	(−)		W	0	1	#N/A	0	#N/A	0	0					1	1	1	1						
1	65	(−)		W	0	1	#N/A	0	#N/A	0	0					0	1	1	1						
1	50	(−)		W	0	1	#N/A	0	#N/A	0	0					1	1	0	1						
1	53	(−)		W	0	1	#N/A	0	#N/A	0	0					0	0	1	0						
1	51	(−)	ケンシュツセス	W	0	1	#N/A	0	#N/A	0	0					1	1	0	1						
1	64	(−)		W	0	1	#N/A	0	#N/A	0	0					0	1	1	1						
1	53	(−)		W	0	1	#N/A	0	#N/A	0	0					1	1	1	1						
1	44	(−)		W	0	1	#N/A	0	#N/A	0	0					1	0	0	1						
2	51	(−)		W	0	1	#N/A	0	#N/A	0	0					1	1	1	1						
1	57	(−)		W	0	1	#N/A	0	#N/A	0	0					1	1	1	1						
1	49	(−)		W	0	1	#N/A	0	#N/A	0	0					1	0	1	1						
2	52	(−)	ケンシュツセス	W	0	1	#N/A	0	#N/A	0	0					0	0	1	0						
1	65	(−)		W	0	1	#N/A	0	#N/A	0	0					0	0	1	0						
1	51	(−)		W	0	1	#N/A	0	#N/A	0	0					0	0	1	0						
1	45	(−)		W	0	1	#N/A	0	#N/A	0	0					1	1	0	1						
1	59			W	0	1	#N/A	0	#N/A	0	0					0	0	1	0						
1	62	(−)		W	0	1	#N/A	0	#N/A	0	0					0	0	1	0						
1	52	(−)		W	0	1	#N/A	0	#N/A	0	0					1	1	1	1						
1	49	(−)		W	0	1	#N/A	0	#N/A	0	0					0	0	1	0						
1	53	(−)		W	0	1	#N/A	0	#N/A	0	0					1	1	1	1						

Sex	Age	HCV-Ab	HCVRNA	status	既感染あ	vaccine	HBsAbscr	HBcAbscr	seroconve	chronic in	Indetermi	observation	TAF(M)	TDF(M)	3TC(M)	TAF(1/0)	TDF(1/0)	3TC(1/0)	FTC	CD4%	CD8%	CD4/8	CD4_count	CD8_count	HIV_RNA
1	50	(−)		W	0	1	#N/A	0	#N/A	0	0					0	1	1	1						
1	54	(−)		W	0	1	#N/A	0	#N/A	0	0					0	1	1	1						
1	49	(−)		W	0	1	#N/A	0	#N/A	0	0					0	1	1	1						
1	47	(−)		W	0	1	#N/A	0	#N/A	0	0					1	1	1	1						
1	56	(−)		W	0	1	#N/A	0	#N/A	0	0					1	1	1	1						
2	47	(−)		W	0	1	#N/A	0	#N/A	0	0					1	0	1	1						
1	56	(−)	ケンシュツセス	W	0	1	#N/A	0	#N/A	0	0					1	1	1	1						
1	57	15.5	2.2	W	0	1	#N/A	0	#N/A	0	0					1	1	1	1						
1	39	(−)		W	0	1	#N/A	0	#N/A	0	0					0	0	1	0						
1	54	(−)		W	0	1	#N/A	0	#N/A	0	0					0	0	1	0						
1	48	(−)		W	0	1	#N/A	0	#N/A	0	0					1	0	0	1						
1	63	(−)		W	0	1	#N/A	0	#N/A	0	0					1	1	1	1						
1	51	(−)		W	0	1	#N/A	0	#N/A	0	0					1	1	1	1						
1	63	2.6	ケンシュツセス	W	0	1	#N/A	0	#N/A	0	0					1	1	1	1						
1	56	(−)		W	0	1	#N/A	0	#N/A	0	0					0	0	1	0						
1	49	(−)		W	0	1	#N/A	0	#N/A	0	0					0	1	1	1						
1	55	(−)		W	0	1	#N/A	0	#N/A	0	0					1	0	1	1						
1	66	(−)		W	0	1	#N/A	0	#N/A	0	0					1	1	1	1						
1	47	(−)	ケンシュツセス	W	0	1	#N/A	0	#N/A	0	0					1	1	1	1						
1	41	(−)		W	0	1	#N/A	0	#N/A	0	0					1	1	0	1						
1	51	(−)		W	0	1	#N/A	0	#N/A	0	0					1	1	1	1						
1	53	(−)	ケンシュツセス	W	0	1	#N/A	0	#N/A	0	0					1	1	1	1						
1	59	(−)		W	0	1	#N/A	0	#N/A	0	0					0	1	1	1						
1	50	(−)	ケンシュツセス	W	0	1	#N/A	0	#N/A	0	0					0	1	1	1						
1	72	(−)		W	0	1	#N/A	0	#N/A	0	0					0	0	1	0						
1	56	(−)		W	0	1	#N/A	0	#N/A	0	0					0	1	1	1						
1	49	(−)	ケンシュツセス	W	0	1	#N/A	0	#N/A	0	0					1	1	0	1						
1	48	(−)		W	0	1	#N/A	0	#N/A	0	0					0	0	1	0						
1	51	(−)		W	0	1	#N/A	0	#N/A	0	0					1	1	0	1						
2	47	(−)		W	0	1	#N/A	0	#N/A	0	0					1	1	1	1						
1	41	(−)		W	0	1	#N/A	0	#N/A	0	0					1	1	0	1						
1	47	(−)		W	0	1	#N/A	0	#N/A	0	0					1	1	0	1						
1	47	(−)	ケンシュツセス	W	0	1	#N/A	0	#N/A	0	0					0	0	1	0						
1	66	(−)		W	0	1	#N/A	0	#N/A	0	0					1	1	1	1						
1	49	(−)		W	0	1	#N/A	0	#N/A	0	0					0	0	1	0						
1	51	(−)		W	0	1	#N/A	0	#N/A	0	0					0	0	1	0						
1	39	(−)		W	0	1	#N/A	0	#N/A	0	0					0	0	1	0						
1	43	(−)		W	0	1	#N/A	0	#N/A	0	0					1	1	0	1						
1	48	(−)		W	0	1	#N/A	0	#N/A	0	0					1	0	0	1						
1	40	(−)		W	0	1	#N/A	0	#N/A	0	0					1	1	0	1						
1	53	(−)		W	0	1	#N/A	0	#N/A	0	0					1	1	1	1						
1	51	(−)		W	0	1	#N/A	0	#N/A	0	0					1	1	1	1						
1	52	(−)	ケンシュツセス	W	0	1	#N/A	0	#N/A	0	0					1	1	0	1						
1	50	(−)		W	0	1	#N/A	0	#N/A	0	0					1	1	0	1						
1	45	(−)		W	0	1	#N/A	0	#N/A	0	0					1	1	0	1						
1	64	(−)	ケンシュツセス	W	0	1	#N/A	0	#N/A	0	0					1	1	1	1						
1	64	(−)		W	0	1	#N/A	0	#N/A	0	0					0	0	1	0						
1	76	(−)		W	0	1	#N/A	0	#N/A	0	0					0	0	1	0						
1	57	(−)		W	0	1	#N/A	0	#N/A	0	0					0	1	1	1						
1	47	(−)		W	0	1	#N/A	0	#N/A	0	0					1	1	0	1						
1	48	(−)		W	0	1	#N/A	0	#N/A	0	0					1	0	1	1						
1	49	(−)		W	0	1	#N/A	0	#N/A	0	0					0	1	1	1						
1	56	(−)		W	0	1	#N/A	0	#N/A	0	0					1	1	1	1						
1	41	(−)		W	0	1	#N/A	0	#N/A	0	0					1	1	0	1						
1	48	2.2	ケンシュツセス	W	0	1	#N/A	0	#N/A	0	0					1	0	1	1						
1	34	39.0	ケンシュツセス	W	0	1	#N/A	0	#N/A	0	0					1	1	0	1						
1	68	(−)		W	0	1	#N/A	0	#N/A	0	0					1	1	0	1						
1	58	(−)		W	0	1	#N/A	0	#N/A	0	0					1	1	1	1						
1	46	(−)		W	0	1	#N/A	0	#N/A	0	0					0	1	1	1						
1	71	(−)		W	0	1	#N/A	0	#N/A	0	0					0	1	1	1						
1	50	(−)		W	0	1	#N/A	0	#N/A	0	0					0	0	1	0						

Sex	Age	HCV-Ab	HCVRNA	status	既感染あ	vaccine	HBsAbscr	HBcAbscr	seroconve	chronic in	Indetermi	observation	TAF(M)	TDF(M)	3TC(M)	TAF(1/0)	TDF(1/0)	3TC(1/0)	FTC	CD4%	CD8%	CD4/8	CD4_coun	CD8_coun	HIV_RNA
1	51	(−)		W	0	1	#N/A	0	#N/A	0	0					0	0	1	0						
2	43	(−)		W	0	1	#N/A	0	#N/A	0	0					1	1	1	1						
1	37	(−)		W	0	1	#N/A	0	#N/A	0	0					1	1	0	1						
1	45	(−)		W	0	1	#N/A	0	#N/A	0	0					0	0	1	0						
1	47	(−)		W	0	1	#N/A	0	#N/A	0	0					1	1	1	1						
1	70	(−)		W	0	1	#N/A	0	#N/A	0	0					1	0	1	1						
1	40		ケンシュツセズ	W	0	1	#N/A	0	#N/A	0	0					1	0	0	1						
1	43	4.0	ケンシュツセズ	W	0	1	#N/A	0	#N/A	0	0					0	1	1	1						
1	63	(−)		W	0	1	#N/A	0	#N/A	0	0					1	1	1	1						
1	48	(−)		W	0	1	#N/A	0	#N/A	0	0					1	1	1	1						
1	65	(−)		W	0	1	#N/A	0	#N/A	0	0					1	1	1	1						
1	42	(−)		W	0	1	#N/A	0	#N/A	0	0					1	1	0	1						
1	48	(−)		W	0	1	#N/A	0	#N/A	0	0					1	1	0	1						
1	51	(−)		W	0	1	#N/A	0	#N/A	0	0					1	1	0	1						
1	51	(−)		W	0	1	#N/A	0	#N/A	0	0					0	0	1	0						
2	47	(−)		W	0	1	#N/A	0	#N/A	0	0					0	1	1	1						
1	46	(−)		W	0	1	#N/A	0	#N/A	0	0					0	1	1	1						
1	48			W	0	1	#N/A	0	#N/A	0	0					1	1	0	1						
1	47	(−)		W	0	1	#N/A	0	#N/A	0	0					1	1	1	1						
1	51	(−)	ケンシュツセズ	W	0	1	#N/A	0	#N/A	0	0					1	1	0	1						
1	50	(−)		W	0	1	#N/A	0	#N/A	0	0					1	1	0	1						
1	41	(−)		W	0	1	#N/A	0	#N/A	0	0					1	1	0	1						
1	45	(−)		W	0	1	#N/A	0	#N/A	0	0					1	0	1	1						
1	39	(−)		W	0	1	#N/A	0	#N/A	0	0					1	0	1	1						
1	66	(−)		W	0	1	#N/A	0	#N/A	0	0					1	1	0	1						
1	61	(−)		W	0	1	#N/A	0	#N/A	0	0					1	1	1	1						
1	37	(−)		W	0	1	#N/A	0	#N/A	0	0					1	0	1	1						
1	37	(−)		W	0	1	#N/A	0	#N/A	0	0					1	0	1	1						
1	55	2.5	ケンシュツセズ	W	0	1	#N/A	0	#N/A	0	0					0	0	1	0						
2	58	(−)		W	0	1	#N/A	0	#N/A	0	0					1	1	0	1						
1	41	(−)		W	0	1	#N/A	0	#N/A	0	0					0	1	1	1						
1	53	(−)		W	0	1	#N/A	0	#N/A	0	0					0	0	1	0						
1	40	(−)		W	0	1	#N/A	0	#N/A	0	0					1	0	1	1						
1	62	(−)		W	0	1	#N/A	0	#N/A	0	0					0	0	1	0						
1	45	(−)		W	0	1	#N/A	0	#N/A	0	0					1	1	1	1						
1	43	(−)		W	0	1	#N/A	0	#N/A	0	0					1	1	0	1						
1	50	(−)		W	0	1	#N/A	0	#N/A	0	0					0	1	1	1						
1	49		ケンシュツセズ	W	0	1	#N/A	0	#N/A	0	0					1	1	0	1						
1	47	(−)		W	0	1	#N/A	0	#N/A	0	0					1	1	0	1						
1	47	(−)		W	0	1	#N/A	0	#N/A	0	0					1	1	0	1						
1	50	(−)		W	0	1	#N/A	0	#N/A	0	0					1	1	0	1						
2	56			W	0	1	#N/A	0	#N/A	0	0					0	1	1	1						
1	43	(−)		W	0	1	#N/A	0	#N/A	0	0					1	1	0	1						
1	38	(−)		W	0	1	#N/A	0	#N/A	0	0					1	1	1	1						
1	52	(−)		W	0	1	#N/A	0	#N/A	0	0					1	1	1	1						
1	50	(−)	ケンシュツセズ	W	0	1	#N/A	0	#N/A	0	0					1	1	0	1						
1	50	(−)		W	0	1	#N/A	0	#N/A	0	0					1	1	1	1						
1	51	(−)		W	0	1	#N/A	0	#N/A	0	0					0	1	1	1						
1	45	(−)	ケンシュツセズ	W	0	1	#N/A	0	#N/A	0	0					1	1	0	1						
1	47	(−)		W	0	1	#N/A	0	#N/A	0	0					1	0	1	1						
1	46	(−)		W	0	1	#N/A	0	#N/A	0	0					1	1	0	1						
1	35	(−)	ケンシュツセズ	W	0	1	#N/A	0	#N/A	0	0					1	1	1	1						
1	33	(−)		W	0	1	#N/A	0	#N/A	0	0					1	0	1	1						
1	49	(−)		W	0	1	#N/A	0	#N/A	0	0					1	1	0	1						
1	42	(−)		W	0	1	#N/A	0	#N/A	0	0					1	1	1	1						
1	47	(−)		W	0	1	#N/A	0	#N/A	0	0					1	0	0	1						
1	72	(−)		W	0	1	#N/A	0	#N/A	0	0					0	0	1	0						
1	40	(−)		W	0	1	#N/A	0	#N/A	0	0					1	1	0	1						
1	47			W	0	1	#N/A	0	#N/A	0	0					0	1	1	1						
1	44	(−)		W	0	1	#N/A	0	#N/A	0	0					1	1	0	1						
1	43	(−)		W	0	1	#N/A	0	#N/A	0	0					1	1	0	1						

Sex	Age	HCV-Ab	HCVRNA	status	既感染あ	vaccine	HBsAbscr	HBcAbscr	seroconve	chronic in	Indetermi	observation	TAF(M)	TDF(M)	3TC(M)	TAF(1/0)	TDF(1/0)	3TC(1/0)	FTC	CD4%	CD8%	CD4/8	CD4_coun	CD8_coun	HIV_RNA
1	44	(−)		W	0	1	#N/A	0	#N/A	0	0					1	1	0	1						
1	49	(−)		W	0	1	#N/A	0	#N/A	0	0					1	1	0	1						
1	41	(−)		W	0	1	#N/A	0	#N/A	0	0					1	1	0	1						
1	46	(−)		W	0	1	#N/A	0	#N/A	0	0					1	1	0	1						
1	40	(−)		W	0	1	#N/A	0	#N/A	0	0					0	0	1	0						
1	40	(−)		W	0	1	#N/A	0	#N/A	0	0					1	1	0	1						
1	47	(−)		W	0	1	#N/A	0	#N/A	0	0					0	0	1	0						
1	48	(−)		W	0	1	#N/A	0	#N/A	0	0					1	1	0	1						
1	56	(−)		W	0	1	#N/A	0	#N/A	0	0					1	1	1	1						
2	47	(−)		W	0	1	#N/A	0	#N/A	0	0					1	1	0	1						
2	68	(−)		W	0	1	#N/A	0	#N/A	0	0					1	1	0	1						
1	39	(−)	ｹﾝｼｭﾂｾｽ	W	0	1	#N/A	0	#N/A	0	0					1	1	0	1						
1	34	(−)		W	0	1	#N/A	0	#N/A	0	0					1	1	0	1						
1	43	(−)		W	0	1	#N/A	0	#N/A	0	0					1	1	0	1						
1	42	(−)		W	0	1	#N/A	0	#N/A	0	0					1	1	0	1						
1	45	(−)		W	0	1	#N/A	0	#N/A	0	0					0	0	1	0						
1	39	(−)	ｹﾝｼｭﾂｾｽ	W	0	1	#N/A	0	#N/A	0	0					1	1	0	1						
1	40	(−)		W	0	1	#N/A	0	#N/A	0	0					1	1	0	1						
1	63	(−)		W	0	1	#N/A	0	#N/A	0	0					1	0	1	1						
1	49	(−)		W	0	1	#N/A	0	#N/A	0	0					1	1	0	1						
1	34	(−)		W	0	1	#N/A	0	#N/A	0	0					0	0	1	0						
1	51	(−)		W	0	1	#N/A	0	#N/A	0	0					0	1	1	1						
1	35	(−)		W	0	1	#N/A	0	#N/A	0	0					1	1	1	1						
2	40	(−)		W	0	1	#N/A	0	#N/A	0	0					1	1	0	1						
1	43	(−)		W	0	1	#N/A	0	#N/A	0	0					1	1	0	1						
1	56	(−)		W	0	1	#N/A	0	#N/A	0	0					1	1	0	1						
1	39	(−)		W	0	1	#N/A	0	#N/A	0	0					1	0	0	1						
1	44	(−)		W	0	1	#N/A	0	#N/A	0	0					1	1	0	1						
1	38	(−)		W	0	1	#N/A	0	#N/A	0	0					0	0	1	0						
1	46	(−)		W	0	1	#N/A	0	#N/A	0	0					1	1	0	1						
1	60	(−)		W	0	1	#N/A	0	#N/A	0	0					1	1	0	1						
1	42	7.0	ｹﾝｼｭﾂｾｽ	W	0	1	#N/A	0	#N/A	0	0					1	1	0	1						
1	45	(−)		W	0	1	#N/A	0	#N/A	0	0					0	1	1	1						
1	42	(−)		W	0	1	#N/A	0	#N/A	0	0					1	1	0	1						
1	55	(−)	ｹﾝｼｭﾂｾｽ	W	0	1	#N/A	0	#N/A	0	0					0	0	1	0						
1	91	(−)		W	0	1	#N/A	0	#N/A	0	0					1	1	1	1						
1	46	(−)		W	0	1	#N/A	0	#N/A	0	0					1	0	1	1						
1	36	(−)		W	0	1	#N/A	0	#N/A	0	0					1	1	0	1						
1	49	17.1	ｹﾝｼｭﾂｾｽ	W	0	1	#N/A	0	#N/A	0	0					1	1	1	1						
1	41	(−)	ｹﾝｼｭﾂｾｽ	W	0	1	#N/A	0	#N/A	0	0					1	1	0	1						
1	46	(−)		W	0	1	#N/A	0	#N/A	0	0					1	1	0	1						
1	35	(−)		W	0	1	#N/A	0	#N/A	0	0					1	1	0	1						
1	54	(−)		W	0	1	#N/A	0	#N/A	0	0					1	1	0	1						
1	55	(−)	ｹﾝｼｭﾂｾｽ	W	0	1	#N/A	0	#N/A	0	0					1	1	1	1						
1	34	(−)		W	0	1	#N/A	0	#N/A	0	0					1	1	0	1						
1	50	(−)		W	0	1	#N/A	0	#N/A	0	0					1	1	0	1						
1	33	(−)		W	0	1	#N/A	0	#N/A	0	0					1	1	0	1						
1	43			W	0	1	#N/A	0	#N/A	0	0					1	1	0	1						
1	49	(−)		W	0	1	#N/A	0	#N/A	0	0					1	1	0	1						
1	57	(−)		W	0	1	#N/A	0	#N/A	0	0					1	1	0	1						
1	46	54.7	ｹﾝｼｭﾂｾｽ	W	0	1	#N/A	0	#N/A	0	0					0	0	1	0						
1	37	(−)	ｹﾝｼｭﾂｾｽ	W	0	1	#N/A	0	#N/A	0	0					1	1	0	1						
1	37	(−)		W	0	1	#N/A	0	#N/A	0	0					0	0	1	0						
1	48	(−)		W	0	1	#N/A	0	#N/A	0	0					1	1	0	1						
1	41	10.0	ｹﾝｼｭﾂｾｽ	W	0	1	#N/A	0	#N/A	0	0					1	0	0	1						
1	35			W	0	1	#N/A	0	#N/A	0	0					1	0	1	1						
1	51	(−)		W	0	1	#N/A	0	#N/A	0	0					0	0	1	0						
1	42	(−)		W	0	1	#N/A	0	#N/A	0	0					1	1	0	1						
1	44	(−)		W	0	1	#N/A	0	#N/A	0	0					1	1	0	1						
1	38	(−)		W	0	1	#N/A	0	#N/A	0	0					1	1	1	1						
1	36	(−)		W	0	1	#N/A	0	#N/A	0	0					1	1	0	1						

Sex	Age	HCV-Ab	HCVRNA	status	既感染あ	vaccine	HBsAbscr	HBcAbscr	seroconve	chronic in	Indetermir	observation	TAF(M)	TDF(M)	3TC(M)	TAF(1/0)	TDF(1/0)	3TC(1/0)	FTC	CD4%	CD8%	CD4/8	CD4_count	CD8_count	HIV_RNA
1	32	(−)		W	0	1	#N/A	0	#N/A	0	0					1	1	1	1						
1	44	(−)		W	0	1	#N/A	0	#N/A	0	0					1	0	0	1						
1	35	(−)		W	0	1	#N/A	0	#N/A	0	0					1	1	0	1						
1	42	(−)		W	0	1	#N/A	0	#N/A	0	0					1	1	0	1						
1	71	(−)		W	0	1	#N/A	0	#N/A	0	0					1	1	0	1						
1	45	(−)		W	0	1	#N/A	0	#N/A	0	0					1	1	0	1						
1	44	(−)		W	0	1	#N/A	0	#N/A	0	0					1	1	0	1						
1	40	(−)		W	0	1	#N/A	0	#N/A	0	0					1	1	0	1						
1	33	(−)		W	0	1	#N/A	0	#N/A	0	0					1	1	0	1						
1	46	(−)		W	0	1	#N/A	0	#N/A	0	0					1	1	0	1						
1	32	(−)		W	0	1	#N/A	0	#N/A	0	0					0	0	1	0						
1	37	(−)		W	0	1	#N/A	0	#N/A	0	0					0	0	1	0						
1	38	(−)		W	0	1	#N/A	0	#N/A	0	0					0	0	1	0						
1	33	(−)		W	0	1	#N/A	0	#N/A	0	0					1	1	0	1						
1	38	(−)		W	0	1	#N/A	0	#N/A	0	0					1	1	0	1						
1	49	(−)		W	0	1	#N/A	0	#N/A	0	0					1	1	0	1						
1	48	(−)		W	0	1	#N/A	0	#N/A	0	0					0	0	1	0						
1	40	(−)		W	0	1	#N/A	0	#N/A	0	0					1	1	0	1						
1	37	(−)		W	0	1	#N/A	0	#N/A	0	0					1	1	0	1						
1	57	(−)		W	0	1	#N/A	0	#N/A	0	0					1	1	0	1						
1	45	(−)		W	0	1	#N/A	0	#N/A	0	0					1	1	0	1						
1	46	(−)		W	0	1	#N/A	0	#N/A	0	0					0	0	1	0						
1	41	(−)		W	0	1	#N/A	0	#N/A	0	0					1	0	1	1						
1	41	(−)		W	0	1	#N/A	0	#N/A	0	0					0	0	1	0						
1	54	(−)		W	0	1	#N/A	0	#N/A	0	0					1	0	0	1						
1	39	(−)		W	0	1	#N/A	0	#N/A	0	0					0	0	1	0						
1	51	(−)		W	0	1	#N/A	0	#N/A	0	0					1	0	0	1						
1	51	(−)		W	0	1	#N/A	0	#N/A	0	0					1	1	0	1						
1	34	(−)		W	0	1	#N/A	0	#N/A	0	0					1	1	0	1						
1	42	(−)		W	0	1	#N/A	0	#N/A	0	0					1	1	1	1						
1	40	(−)		W	0	1	#N/A	0	#N/A	0	0					1	0	1	1						
1	54	(−)		W	0	1	#N/A	0	#N/A	0	0					1	1	0	1						
1	34	(−)		W	0	1	#N/A	0	#N/A	0	0					1	1	0	1						
1	46	(−)		W	0	1	#N/A	0	#N/A	0	0					0	1	1	1						
1	32	(−)		W	0	1	#N/A	0	#N/A	0	0					1	0	1	1						
1	30	(−)		W	0	1	#N/A	0	#N/A	0	0					1	1	0	1						
1	55	(−)		W	0	1	#N/A	0	#N/A	0	0					1	0	0	1						
1	30	(−)		W	0	1	#N/A	0	#N/A	0	0					0	0	1	0						
2	41	(−)		W	0	1	#N/A	0	#N/A	0	0					1	1	0	1						
1	34	(−)		W	0	1	#N/A	0	#N/A	0	0					1	1	0	1						
1	35	(−)		W	0	1	#N/A	0	#N/A	0	0					0	0	1	0						
1	35	(−)		W	0	1	#N/A	0	#N/A	0	0					1	1	0	1						
1	37	(−)		W	0	1	#N/A	0	#N/A	0	0					0	0	1	0						
1	42	(−)		W	0	1	#N/A	0	#N/A	0	0					1	0	1	1						
1	40	(−)		W	0	1	#N/A	0	#N/A	0	0					0	0	1	0						
1	49	(−)		W	0	1	#N/A	0	#N/A	0	0					1	0	0	1						
1	35	(−)		W	0	1	#N/A	0	#N/A	0	0					0	0	1	0						
1	31	(−)		W	0	1	#N/A	0	#N/A	0	0					0	0	1	0						
1	50	(−)	ｹﾝｼｭﾂｾｽ	W	0	1	#N/A	0	#N/A	0	0					1	1	0	1						
1	29	(−)		W	0	1	#N/A	0	#N/A	0	0					0	0	1	0						
1	35	(−)		W	0	1	#N/A	0	#N/A	0	0					0	0	1	0						
1	34	(−)		W	0	1	#N/A	0	#N/A	0	0					1	1	0	1						
1	49	(−)		W	0	1	#N/A	0	#N/A	0	0					1	1	1	1						
1	35	(−)		W	0	1	#N/A	0	#N/A	0	0					1	1	0	1						
1	37	(−)		W	0	1	#N/A	0	#N/A	0	0					1	1	0	1						
1	35	(−)		W	0	1	#N/A	0	#N/A	0	0					1	1	0	1						
1	49	(−)		W	0	1	#N/A	0	#N/A	0	0					1	1	0	1						
1	33	(−)		W	0	1	#N/A	0	#N/A	0	0					0	0	1	0						
1	43	(−)		W	0	1	#N/A	0	#N/A	0	0					1	1	0	1						
1	41	(−)		W	0	1	#N/A	0	#N/A	0	0					0	0	1	0						
1	33	(−)		W	0	1	#N/A	0	#N/A	0	0					1	1	0	1						

Sex	Age	HCV-Ab	HCVRNA	status	既感染あ	vaccine	HBsAbscr	HBcAbscr	seroconve	chronic in	Indetermir	observation	TAF(M)	TDF(M)	3TC(M)	TAF(1/0)	TDF(1/0)	3TC(1/0)	FTC	CD4%	CD8%	CD4/8	CD4_count	CD8_count	HIV_RNA
1	31	(-)		W	0	1	#N/A	0	#N/A	0	0					1	1	1	1						
1	30	(-)		W	0	1	#N/A	0	#N/A	0	0					0	0	1	0						
1	35			W	0	1	#N/A	0	#N/A	0	0					0	0	1	0						
1	48	(-)		W	0	1	#N/A	0	#N/A	0	0					0	0	1	0						
1	42	(-)		W	0	1	#N/A	0	#N/A	0	0					0	0	1	0						
1	43	(-)		W	0	1	#N/A	0	#N/A	0	0					0	0	1	0						
1	40	(-)		W	0	1	#N/A	0	#N/A	0	0					1	0	0	1						
1	38	(-)		W	0	1	#N/A	0	#N/A	0	0					1	0	0	1						
1	35	(-)		W	0	1	#N/A	0	#N/A	0	0					1	0	0	1						
1	39	40.3	ｹﾝｼｭﾂｾｽ	W	0	1	#N/A	0	#N/A	0	0					1	0	1	1						
2	36	(-)		W	0	1	#N/A	0	#N/A	0	0					1	1	1	1						
1	30	(-)		W	0	1	#N/A	0	#N/A	0	0					0	0	1	0						
1	35	(-)		W	0	1	#N/A	0	#N/A	0	0					1	1	0	1						
1	28	(-)		W	0	1	#N/A	0	#N/A	0	0					0	1	1	1						
1	27	(-)		W	0	1	#N/A	0	#N/A	0	0					1	0	0	1						
1	47	(-)		W	0	1	#N/A	0	#N/A	0	0					0	0	1	0						
1	50	(-)	ｹﾝｼｭﾂｾｽ	W	0	1	#N/A	0	#N/A	0	0					1	0	1	1						
1	52	(-)		W	0	1	#N/A	0	#N/A	0	0					1	1	0	1						
1	28	(-)		W	0	1	#N/A	0	#N/A	0	0					1	0	1	1						
1	37	(-)		W	0	1	#N/A	0	#N/A	0	0					1	0	0	1						
1	34	(-)		W	0	1	#N/A	0	#N/A	0	0					0	0	1	0						
1	47	(-)		W	0	1	#N/A	0	#N/A	0	0					1	0	0	1						
2	38	(-)		W	0	1	#N/A	0	#N/A	0	0					1	1	0	1						
1	41	(-)		W	0	1	#N/A	0	#N/A	0	0					1	1	1	1						
1	38	(-)		W	0	1	#N/A	0	#N/A	0	0					0	0	1	0						
1	35	(-)		W	0	1	#N/A	0	#N/A	0	0					0	0	1	0						
1	41	(-)		W	0	1	#N/A	0	#N/A	0	0					0	0	1	0						
1	43	(-)		W	0	1	#N/A	0	#N/A	0	0					1	0	0	1						
1	38	(-)		W	0	1	#N/A	0	#N/A	0	0					0	0	1	0						
1	40	(-)		W	0	1	#N/A	0	#N/A	0	0					1	1	0	1						
1	50	(-)		W	0	1	#N/A	0	#N/A	0	0					1	1	0	1						
1	34	(-)		W	0	1	#N/A	0	#N/A	0	0					1	0	1	1						
1	44	(-)		W	0	1	#N/A	0	#N/A	0	0					1	0	0	1						
1	32	(-)		W	0	1	#N/A	0	#N/A	0	0					1	0	1	1						
1	34	(-)		W	0	1	#N/A	0	#N/A	0	0					1	0	0	1						
1	30	(-)		W	0	1	#N/A	0	#N/A	0	0					1	0	0	1						
1	59	(-)		W	0	1	#N/A	0	#N/A	0	0					1	0	1	1						
1	50	(-)		W	0	1	#N/A	0	#N/A	0	0					1	0	1	1						
1	26	(-)		W	0	1	#N/A	0	#N/A	0	0					1	0	0	1						
1	30	(-)		W	0	1	#N/A	0	#N/A	0	0					1	0	1	1						
1	31	(-)		W	0	1	#N/A	0	#N/A	0	0					1	0	1	1						
1	28	(-)		W	0	1	#N/A	0	#N/A	0	0					0	0	1	0						
1	38	(-)		W	0	1	#N/A	0	#N/A	0	0					1	0	0	1						
1	39	(-)		W	0	1	#N/A	0	#N/A	0	0					1	0	0	1						
1	54	(-)		W	0	1	#N/A	0	#N/A	0	0					1	0	0	1						
1	29	(-)		W	0	1	#N/A	0	#N/A	0	0					1	0	0	1						
1	34	(-)		W	0	1	#N/A	0	#N/A	0	0					1	0	0	1						
1	40	(-)		W	0	1	#N/A	0	#N/A	0	0					1	0	0	1						
1	36	(-)		W	0	1	#N/A	0	#N/A	0	0					1	0	0	1						
1	42	(-)		W	0	1	#N/A	0	#N/A	0	0					1	0	0	1						
1	41	(-)		W	0	1	#N/A	0	#N/A	0	0					1	0	0	1						
1	58	(-)		W	0	1	#N/A	0	#N/A	0	0					0	0	1	0						
1	31	(-)		W	0	1	#N/A	0	#N/A	0	0					1	0	0	1						
1	34	(-)	ｹﾝｼｭﾂｾｽ	W	0	1	#N/A	0	#N/A	0	0					1	0	0	1						
1	29	(-)	ｹﾝｼｭﾂｾｽ	W	0	1	#N/A	0	#N/A	0	0					1	0	0	1						
