# Supplementary material for: Serology change-based clinical interpretation of indeterminate serostatus post-hepatitis B virus infection in people living with HIV
Source: PLoS One. 2025 Nov 20;20(11):e0336924. doi: 10.1371/journal.pone.0336924 (PMC12633944; doi:10.1371/journal.pone.0336924)
Supplement: Supporting information 2 — (PDF) [file pone.0336924.s004.pdf]

HCV抗体	HBs抗原	HBsAg_limit	HBs抗体	HBsAb_limit	HBe抗原	HBeAg_limit	HBe抗体	HBeAb_limit	HBc抗体	HBcAb_limit	HBVDNA	HBVDNA_limit	Notes
(-)		(-)		(-)					11.0				
(-)		(-)											
(-)		(-)		(-)					9.0				
(-)				(-)									
(-)				(-)									
(-)		(-)		(-)		(-)	78.0		99.0				
(-)		(-)		(-)					99.0				
(-)		(-)		(-)					98.0				
(-)			97.1										
(-)			16.7										
(-)		(-)	9.6						48.1				
(-)		(-)	21.3						10.8				
(-)			14.8										
(-)		(-)		(-)					9.5				
(-)		(-)	11.7						8.7				
(-)		(-)	12.8						6.8				
(-)		(-)											
(-)			15.7										
(-)		(-)		(-)					98.0				
(-)		(-)		(-)					33.8				
(-)		(-)		(-)					9.9				
(-)		(-)		(-)					9.0				
(-)		(-)		(-)									
(-)		(-)		(-)									
(-)				(-)									
(-)				(-)									
(-)		(-)		(-)					96.0				
(-)		(-)		(-)					96.0				
(-)		(-)		(-)					8.4				
(-)		(-)		(-)					8.8				
(-)		(-)											
(-)		(-)		(-)									
(-)				(-)									
(-)				(-)									
(-)				(-)									
(-)		(-)							88.0				
(-)		(-)	406.6						82.0				
(-)		(-)	1000.0	<						(-)			
(-)		(-)	1000.0	<					1.8				
(-)		(-)	1000.0	<					1.3				
(-)		(-)											
(-)		(-)											
(-)			1000.0	<									
(-)			1000.0	<					1.2				
(-)		(-)											
(-)		(-)											
(-)				(-)					8.7				
(-)				(-)									
(-)				(-)									
(-)		(-)	27.6						80.0				
(-)		(-)	36.8						3.3				
(-)		(-)	33.1						3.2				
(-)		(-)	28.2						3.2				
(-)		(-)											
(-)		(-)	14.5						2.8				
(-)		(-)		(-)					2.4				
(-)			11.4										
(-)		(-)	644.2							(-)			
(-)		(-)	714.2							(-)			
(-)		(-)	422.5						1.0				
(-)		(-)											
(-)			816.0										
(-)											0.0	4	
(-)		(-)	126.6			(-)	100.0				3.7	3	HBV(TMA)、IgM-HBc抗体　0
(-)		(-)											
(-)		(-)		(-)									
(-)		(-)		(-)					17.6				
(-)		(-)		(-)									
(-)		(-)		(-)					8.0				
(-)		(-)		(-)					8.0		0.0	4	
(-)				(-)					6.1				
(-)											0.0	4	
(-)		(-)											
(-)		(-)	383.5							(-)			
(-)		(-)	70.2						1.1				
(-)		(-)	139.7										
(-)			87.2										
(-)			81.1										

(-)	(-)	6.6				92.0				
(-)	(-)		(-)			11.9				
(-)	(-)		(-)			11.1				
(-)	(-)		(-)			8.9				
(-)	(-)		(-)			8.8				
(-)	(-)		(-)			8.3				
(-)	(-)	11.5				7.5				
(-)	(-)	18.9				6.4				
(-)		122.7				5.3				
(-)		137.3				5.3				
(-)			(-)							
(-)	(-)		(-)							
(-)	(-)		(-)			97.0				
(-)	(-)		(-)	(-)	(-)	96.0				
(-)	(-)		(-)			95.0		0.0	4	HBVDNA定量
(-)	(-)		(-)			23.1		0.0	4	
(-)	(-)		(-)			7.6				
(-)	(-)		(-)			6.0				
(-)			(-)			5.8		0.0	4	
(-)								0.0	4	
(-)	(-)									
(-)	(-)									
(-)	(-)									
15.1	(-)									
15.1	(-)	27.0				85.0				
15.1	(-)	10.2				7.2				
15.1	(-)	14.1				5.8				
15.1	(-)	14.3			(-)	6.0				
15.1	(-)									
15.1								0.0	4	
15.1	(-)		(-)			5.5				
15.1	(-)		(-)			6.3				
15.1				(-)	(-)			0.0	4	
15.1								0.0	4	
15.1								0.0	4	
15.1	(-)	94.5		(-)	(-)			0.0	4	
15.1	(-)	60.5		(-)	(-)			0.0	4	
15.1	(-)									
15.1	(-)									
15.1			(-)			4.4				
(-)	(-)		(+/-)	(-)	(-)	65.0				
(-)	(-)	8.7		(-)	(-)		(-)	0.0	4	
(-)	(-)		(-)				(-)			
(-)	(-)		(-)				(-)			
(-)	(-)									
(-)	(-)		(-)			92.0				
(-)	(-)		(-)							
(-)			(-)							
(-)			(-)							
(-)			(-)							
55.0	(-)									
55.0	(-)									
55.0	(-)									
55.0	(-)									
55.0	(-)									
55.0	(-)		(-)			110.7				
55.0								0.0	4	
55.0								0.0	4	
55.0	(-)		(-)							
55.0	(-)		(-)	(-)	(-)	9.4		0.0	4	
55.0	(-)									
55.0	(-)									
55.0	(-)									
55.0	(-)									
(-)	(-)									
(-)	(-)	185.3					(-)			
(-)	(-)	266.8				1.5				
(-)	(-)	111.7				1.5				
(-)	(-)	105.4				1.4				
(-)	(-)	90.0				95.0				
(-)	(-)		(-)	(-)	(-)	8.3				
(-)		54.0				7.7				
(-)	(-)	47.4				93.0				
(-)	(-)	26.0				14.1				
(-)	(-)	12.7				8.5				
(-)	(-)									
(-)	(-)		(-)			5.8				
(-)	(-)		(-)			5.3				
(-)	(-)									

(-)		(-)											
(-)			21.0										
(-)		(-)											
(-)		(-)											
(-)			20.2						99.0				
(-)		(-)		(-)					37.6				
(-)		(-)	59.6						8.2		0.0	4	
(-)		(-)											
(-)											0.0	4	
(-)											0.0	4	
(-)											0.0	4	
(-)											0.0	4	
(-)											0.0	4	
(-)											0.0	4	
(-)											0.0	4	
(-)											0.0	4	
(-)											0.0	4	
(-)											0.0	4	
(-)											0.0	4	
(-)											0.0	4	
(-)											0.0	4	
(-)		(-)	1000.0	<					7.0				
(-)		(-)	455.8										
(-)		(-)	570.5										
(-)		(-)	567.0										
(-)		(-)		(-)		(-)		(-)					
(-)		(-)		(-)									
(-)		(-)		(-)		(-)		(-)	95.0		0.0	4	HBVDNA定量
(-)		(-)		(-)							0.0	4	HBVDNA定量
(-)											0.0	4	HBVDNA定量
(-)											0.0	4	HBVDNA定量
(-)											0.0	4	HBVDNA定量
(-)		(-)		(-)		(-)		(-)	29.3		0.0	4	
(-)		(-)		(-)					7.8				
(-)											0.0	4	
(-)		(-)		(-)					8.3				
(-)						(-)		(-)	7.6		0.0	4	
(-)		(-)				(-)			6.9		0.0	4	
(-)			12.7						4.3		0.0	4	
(-)			12.0						3.5		0.0	4	
(-)			16.7						3.8				
(-)			19.3						3.8				
(-)			20.4						3.6				
(-)		(-)											
(-)		(-)	23.8						94.0				
(-)		(-)	23.8						93.0				
(-)		(-)	11.8						14.1				
(-)		(-)		(-)					9.9				
(-)		(-)		(-)					8.1				
(-)		(-)	31.9						8.5				
(-)		(-)	12.0						7.3				
(-)		(-)							8.0				
(-)		(-)							7.9				
(-)				(-)					8.2				
(-)				(-)					7.4				
(-)				(-)					6.5				
(-)		(-)	13.7						91.0				
(-)		(-)	49.2						1.9				
(-)		(-)	21.0						2.0				
(-)		(-)											
(-)		(-)		(-)									
(-)		(-)	10.6										
(-)			10.6										
(-)				(-)									
(-)		(-)		(-)					87.0				
(-)		(-)		(-)					4.8				
(-)		(-)		(-)		(-)		(-)	4.1				
(-)		(-)											
(-)		(-)							3.4				
(-)		(-)		(-)									
(-)		(-)		(-)					3.3				
(-)											0.0	4	
(-)		(-)		(-)									
(-)				(-)									
(-)		(-)	85.8			(-)			93.0				
(-)		(-)	80.7			(-)		(-)	92.0				
(-)		(-)	71.5			(-)		(-)	92.0				
(-)		(-)	34.2						13.9				
(-)		(-)		(-)									

(-)	(-)	12.9				7.9				
(-)		19.8								
(-)	(-)	81.2		(-)	(-)	89.0				
(-)	(-)	80.8		(-)	(-)	88.0		0.7	3	HBV-DNA定量
(-)	(-)	201.5		(-)	(-)		(-)	2.6	3	HBVDNA-PCR
(-)	(-)	162.4		(-)	(-)		(-)	2.6	3	HBVDNA-PCR
(-)	(-)	135.6		(-)	(-)	52.0		2.6	3	HBVDNA-PCR
(-)	(-)	72.9		(-)	(-)		(-)	1.8	3	HBVDNA定量
(-)	(-)	122.3		(-)	(-)		(-)			
(-)	(-)	107.5		(-)	(-)		(-)	0.0	4	HBVDNA定量
(-)	(-)	96.3		(-)	(-)		(-)	0.0	4	HBcrAg　3.0ﾐﾏﾝ、HBVDNA定
(-)	(-)	173.7		(-)	(-)	2.0		0.0	4	HBcrAg　3.0ﾐﾏﾝ
(-)	(-)	54.2				1.6				
(-)	(-)	51.2		(-)	(-)	1.3				
(-)	(-)	125.7				1.3				
(-)	(-)	111.9		(-)	(-)					HBcrAg　<3.0
(-)	(-)	116.7		(-)	(-)					HBcrAg　<3.0
(-)	(-)	119.8		(-)	(-)					HBcrAg　<3.0
(-)	(-)	119.5								
(-)	(-)	105.4		(-)	(-)					HBcrAg　<3.0
(-)	(-)	76.7		(-)	(-)					HBcrAg　<3.0
(-)	(-)	64.4		(-)	(-)					HBcrAg　<3.0
(-)		61.1								
(-)	(-)		(-)			90.0				
(-)	(-)		(-)	(-)	(-)	8.0				
(-)	(-)		(-)			6.8				
(-)	(-)		(-)			6.7				
(-)	(-)									
(-)			(-)			4.4				
(-)	(-)	20.2				73.0				
(-)	(-)		(-)				(-)			
(-)	(-)	52.8					(-)			
(-)	(-)									
(-)		13.1					(-)			
(-)								0.0	4	
(-)	(-)		(-)							
(-)		10.3					(-)			
(-)			(-)				(-)			
(-)	(-)		(-)							
(-)		10.8								
(-)								0.0	4	
(-)								0.0	4	
(-)								0.0	4	
1.7	(-)									
1.7		39.7								
1.7	(-)	24.9		(-)	(-)	53.0				
1.7	(-)	1000.0	<				(-)			
1.7	(-)	128.0					(-)			
1.7	(-)	37.5				1.2				
1.7	(-)	45.8					(-)			
1.7	(-)	26.3		(-)	(-)		(-)			
1.7	(-)	28.1		(-)	(-)		(-)			
1.7	(-)	24.1		(-)	(-)		(-)			
(-)	(-)	2.5								
(-)	(-)	63.4				83.0				
(-)	(-)		(-)			79.0				
(-)	(-)	8.3				80.0				
(-)	(-)	27.5				84.0				
(-)	(-)	18.6				76.0				
(-)	(-)	13.8				3.9				
(-)	(-)									
(-)	(-)									
(-)	(-)	16.1								
(-)		23.4								
(-)		32.1								
(-)		20.0								
(-)	(-)		(-)			89.0				
(-)	(-)		(-)	(-)	(-)	88.0		0.0	4	HBVDNA定量
(-)			(-)							
(-)	(-)		(-)	(-)	(-)	88.0		0.0	4	HBVDNA定量
(-)		18.7								
(-)	(-)	271.5				85.0				
(-)	(-)	209.1				3.9				
(-)	(-)	191.7				3.2				
(-)		291.8				3.3				
(-)	(-)	113.4				2.1				
(-)		128.7				2.2				
(-)	(-)	85.8				1.2				
(-)		454.2								

(-)		(-)									0.0	4	
(-)			336.8										
(-)		(-)	5.0			(-)	78.0		98.0		2.6	3	HBVDNA-PCR
(-)		(-)	43.1						87.0				
(-)		(-)											
(-)			1000.0	<						(-)			
(-)											0.0	4	
(-)											1.0	3	
(-)											0.0	4	
(-)											0.0	4	
(-)											0.0	4	
(-)											0.0	4	
(-)											0.0	4	
(-)											0.0	4	
(-)											0.0	4	
(-)											0.0	4	
(-)											0.0	4	
(-)											0.0	4	
(-)											0.0	4	
(-)											0.0	4	
(-)											0.0	4	
(-)											0.0	4	
(-)		(-)											
(-)											0.0	4	
(-)											0.0	4	
(-)											0.0	4	
(-)											0.0	4	
(-)		(-)											
(-)											0.0	4	
(-)											0.0	4	
(-)											0.0	4	
(-)											0.0	4	
(-)	2000.00	<		(-)	733.8			(-)	18500.0				
(-)											6.4	1	HBVDNA定量、IgM-HBc抗体
(-)						(-)	100.0						
(-)											2.4	1	HBVDNA定量
(-)		(-)		(-)		(-)	99.0				2.2	1	HBVDNA定量
(-)		(-)		(-)		(-)	93.0				2.1	3	HBVDNA定量
(-)		(-)		(-)		(-)	92.0				0.0	4	HBVDNA定量
(-)		(-)		(-)		(-)	94.0				0.0	4	HBVDNA定量
(-)		(-)		(-)		(-)	74.0				0.0	4	HBVDNA定量
(-)		(-)		(-)					162.3		0.0	4	
(-)		(-)	62.7						10.5				
(-)							57.0						
(-)		(-)	54.6						70.0				
(-)		(-)	674.9							(-)			
(-)		(-)	681.8							(-)			
(-)		(-)											
(-)		(-)	137.4							(-)			
(-)		(-)	146.9							(-)			
(-)			344.5							(-)			
(-)			405.8							(-)			
(-)		(-)											
(-)		(-)											
(-)		(-)											
(-)		(-)											
(-)		(-)											
(-)				(-)					84.0				
(-)		(-)											
(-)		(-)		(-)		(-)		(-)	81.0		0.0	4	HBVDNA定量、HBcrAg　3.0[illegible]
(-)		(-)											
(-)		(-)		(-)					4.0				
(-)		(-)		(-)					3.3				
(-)		(-)		(-)									
(-)		(-)											
(-)		(-)		(-)									
(-)				(-)									
(-)				(-)									
(-)				(-)									
(-)		(-)	1000.0	<		(-)		(-)	77.0				
(-)		(-)	1000.0	<					57.0				
(-)		(-)	452.0						2.0				
(-)		(-)	439.2						1.7				
(-)		(-)	728.8						1.5				
(-)													HBcrAg　<3.0
(-)		(-)	1000.0	<					1.3				
(-)			952.3						1.3				
(-)			1000.0	<					1.1				
(-)			1000.0	<					1.1				

(-)			863.5							(-)			
(-)		(-)											
(-)			29.7						97.0				
(-)					0.5	>	49.0	(-)			0.0	4	HBe抗原[RIA]、HBe抗体[RIA]
(-)		(-)	19.1			(-)	51.0				0.0	4	HBVDNA定量
(-)		(-)	30.4			(-)	54.0				0.0	4	
(-)		(-)	23.5			(-)	52.0				0.0	4	
(-)		(-)	24.3			(-)	51.0				0.0	4	
(-)													HBcrAg　<3.0
(-)											0.0	4	
(-)				(-)									
(-)		(-)							6.3				
1.0		(-)											
1.0		(-)	5.0						96.0				
1.0		(-)											
1.0		(-)		(-)									
1.0		(-)		(-)					5.2				
1.0											0.0	4	
1.0			16.9			(-)			4.2				
(-)		(-)	806.6						1.2				
(-)		(-)	430.8							(-)			
(-)		(-)	702.9							(-)			
(-)		(-)	642.1						53.0				
(-)		(-)	441.1			(-)		(-)		(-)	0.0	4	
(-)		(-)	220.2							(-)			
(-)		(-)	117.3							(-)			
(-)		(-)	161.8							(-)			
(-)		(-)	162.0							(-)			
(-)			92.8							(-)			
(-)		(-)											
(-)			88.8							(-)			
(-)											2.6	3	HBVDNA-PCR
(-)											2.6	3	HBVDNA-PCR
(-)		(-)		(-)		(-)		(-)		(-)	2.6	3	HBVDNA-PCR
(-)											2.6	3	HBVDNA-PCR
(-)		(-)		(-)		(-)		(-)	99.0		0.0	4	HBVDNA定量
(-)		(-)		(-)		(-)		(-)	99.0		0.0	4	HBVDNA定量
(-)		(-)		(-)		(-)		(-)	99.0		0.0	4	HBVDNA定量
(-)											0.0	4	HBVDNA定量
(-)		(-)		(-)		(-)		(-)	99.0		0.0	4	HBVDNA定量、HBcrAg　3.5
(-)											0.0	4	HBVDNA定量、HBcrAg　3.6
(-)											0.0	4	HBVDNA定量
(-)		(-)		(-)		(-)		(-)	99.0		0.0	4	HBVDNA定量
(-)											0.0	4	HBVDNA定量
(-)											0.0	4	HBVDNA定量
(-)		(-)		(-)		(-)		(-)	99.0		0.0	4	HBVDNA定量
(-)											0.0	4	
(-)											2.1	3	
(-)											0.0	4	HBcrAg　3.4
(-)		(-)		(-)		(-)		(-)	97.9		0.0	4	
(-)											0.0	4	
(-)		(-)		(-)		(-)		(-)	91.1		2.1	3	
(-)											0.0	4	
(-)		(-)		(-)		(-)		(-)	87.1		0.0	4	
(-)											0.0	4	
(-)		(-)		(-)					10.5				
(-)		(-)		(-)		(-)		(-)	10.1		0.0	4	
(-)													HBcrAg　3.1
(-)											0.0	4	
(-)		(-)		(-)					9.5				
(-)		(-)		(-)					9.0				
(-)											0.0	4	
(-)		(-)		(-)		(-)		(-)	8.5		0.0	4	
(-)				(-)				(-)	9.5				
(-)		(-)											HBcrAg　<3.0
(-)		(-)		(-)		(-)		(-)	8.2				
(-)		(-)		(-)					7.1				
(-)		(-)											
(-)		(-)		(-)					7.4				
(-)		(-)		(-)					7.5				
(-)				(-)					8.6				
(-)				(-)					7.2				
(-)		(-)											
(-)		(-)		(-)					97.0				
(-)			10.0	>									HBs抗体CLIA
(-)				(-)									
(-)				(-)									
(-)			6.9										
(-)				(-)									

(-)		(-)	43.6										
(-)		(-)	42.1										
(-)		(-)	41.9						10.8				
(-)			19.1						8.4				
(-)		(-)	12.7						95.0				
(-)		(-)											
(-)		(-)		(-)					94.0				
(-)		(-)	316.3						18.6				
(-)		(-)	359.7						17.9				
(-)		(-)	267.5						8.9				
(-)		(-)	309.2						9.5				
(-)		(-)	274.3						9.0				
(-)		(-)	348.5						7.0				
(-)		(-)	421.4						8.1				
(-)			316.1										
(-)		(-)											
(-)		(-)	584.9										
(-)		(-)	1000.0	<		(-)		(-)	52.0				
(-)		(-)	963.0										
(-)			240.5										
(-)		(-)	798.1			(-)		(-)	1.1				
(-)		(-)	974.1										
(-)		(-)	1000.0	<					1.0				
(-)		(-)	777.9			(-)		(-)		(-)			
(-)		(-)	836.0										
(-)		(-)	781.0										
(-)			868.9							(-)			
(-)		(-)	775.3										
(-)		(-)	42.8						88.0				
(-)		(-)	24.4						1.3				
(-)		(-)		(-)					2.2				
(-)		(-)		(-)					1.7				
(-)		(-)											
(-)		(-)		(-)					1.3				
(-)		(-)		(-)					1.7				
(-)			13.3										
(-)				(-)					1.2				
(-)		(-)											
(-)		(-)											
(-)		(-)	1000.0	<					56.0				
(-)		(-)	1000.0	<						(-)			
(-)		(-)	1000.0	<					2.0				
(-)		(-)	1000.0	<					1.7				
(-)		(-)	1000.0	<					2.0				
(-)		(-)	1000.0	<					1.4				
(-)		(-)	1000.0	<					1.5				
(-)		(-)											
(-)			1000.0	<									
(-)			1000.0	<					1.4				
(-)		(-)											
2.4	500.00	<		(-)	347.4			(-)		(-)			
2.4											7.7	2	HBVDNA-PCR
2.4	2000.00	<		(-)	1600.0	<		(-)	58.0		7.7	2	HBVDNA-PCR、HBV(TMA)
2.4											3.5	1	HBVDNA-PCR
2.4	2000.00	<		(-)	1265.2			(-)	79.0		3.5	1	HBVDNA-PCR
2.4	2000.00	<		(-)	1234.6			(-)	75.0		3.2	1	HBVDNA-PCR
2.4											3.3	1	HBVDNA-PCR
2.4	2000.00	<		(-)	636.4			(-)	80.0		5.2	1	HBV(TMA)
2.4	2000.00	<		(-)	279.0			(-)	81.0		5.3	1	HBV(TMA)
2.4	2000.00	<		(-)	245.6			(-)	68.0		4.6	1	HBV(TMA)
2.4											2.1	1	HBVDNA定量
2.4											1.8	3	HBVDNA定量
2.4											2.1	3	HBVDNA定量
2.4	2000.00	<		(-)						(-)	2.1	3	HBVDNA定量
2.4											0.0	4	HBVDNA定量、HBcrAg　5.5
2.4	2000.00	<		(-)						(-)	2.1	3	
2.4											0.0	4	
2.4	2000.00	<		(-)	4.5			(-)		(-)	2.1	3	
2.4	4.10			(-)		(-)		(-)	8.4		0.0	4	
2.4	4.30			(-)		(-)		(-)	6.8		0.0	4	
2.4	0.56			(-)		(-)		(-)	3.1		0.0	4	
2.4	0.59			(-)		(-)		(-)	2.3		0.0	4	
2.4	0.39			(-)		(-)		(-)	2.4		2.1	3	
2.4	0.19			(-)		(-)		(-)		(-)	0.0	4	
2.4	0.26			(-)		(-)		(-)		(-)	0.0	4	
2.4											2.1	3	
2.4	0.24			(-)							0.0	4	
2.4											0.0	4	
2.4											0.0	4	

2.4											0.0	4	
2.4	0.29			(-)		(-)		(-)		(-)			
2.4	0.30			(-)							0.0	4	
2.4	0.26					(-)		(-)		(-)			
2.4	0.39					(-)		(-)		(-)	0.0	4	
2.4	0.26			(-)		(-)		(-)		(-)	0.0	4	
2.4	0.32			(-)		(-)		(-)		(-)	0.0	4	
2.4	0.39			(-)		(-)		(-)			0.0	4	
2.4											0.0	4	
2.4	0.43												
2.4	0.57			(-)							0.0	4	
2.4											0.0	4	
2.4	0.44			(-)							0.0	4	
2.4											0.0	4	
2.4	0.39												
2.4											0.0	4	
2.4											0.0	4	
2.4											0.0	4	
2.4											0.0	4	
2.4											0.0	4	
(-)		(-)		(-)					12800.0				
(-)		(-)	6.3						97.0		0.0	4	HBVDNA定量
(-)		(-)		(-)					57.2				
(-)		(-)		(-)					10.7				
(-)		(-)		(-)					8.9				
(-)				(-)					6.3		0.0	4	
(-)	500.00	<		(-)	377.2			(-)		(-)			
(-)											8.6	1	HBV(TMA)
(-)	500.00	<		(-)	348.1			(-)			7.1	1	IgM-HBc　(-)、HBV(TMA)
(-)	500.00	<		(-)	321.2			(-)		(-)			
(-)	500.00	<		(-)	298.8			(-)		(+/-)	0.7	3	HBV-DNA定量
(-)	500.00	<		(-)	311.4			(-)		(+/-)	0.7	3	HBV-DNA定量
(-)	500.00	<		(-)	280.0			(-)		(+/-)	0.7	3	HBV-DNA定量
(-)	500.00	<		(-)	402.1			(-)		(+/-)	2.6	3	HBVDNA-PCR
(-)	500.00	<		(-)	306.7			(-)		(+/-)	2.6	3	HBVDNA-PCR
(-)	500.00	<		(-)	305.5			(-)		(+/-)	2.6	3	HBVDNA-PCR
(-)	2000.00	<		(-)	1600.0	<		(-)	50.0		2.6	3	HBVDNA-PCR
(-)	2000.00	<		(-)	1600.0	<		(-)		(-)	2.6	3	HBVDNA-PCR
(-)	2000.00	<		(-)	1600.0	<		(-)		(-)	2.6	3	HBVDNA-PCR
(-)	2000.00	<		(-)	1432.8			(-)		(-)	2.6	3	HBVDNA-PCR
(-)	2000.00	<		(-)	1169.4			(-)	53.0		2.6	3	HBVDNA-PCR
(-)	2000.00	<		(-)	1125.1			(-)	50.0		2.6	3	HBVDNA-PCR
(-)	2000.00	<		(-)	1185.0			(-)		(-)	1.8	3	HBVDNA定量
(-)	2000.00	<		(-)	1148.3			(-)		(-)	0.0	4	HBVDNA定量
(-)	2000.00	<		(-)	1047.7			(-)		(-)	2.1	3	HBVDNA定量
(-)	2000.00	<		(-)	923.5			(-)	51.0		2.1	3	HBVDNA定量
(-)	2000.00	<		(-)	812.6			(-)		(-)	0.0	4	HBVDNA定量
(-)	2000.00	<		(-)	506.5			(-)	55.0		0.0	4	HBVDNA定量
(-)	2000.00	<		(-)	389.7			(-)	56.0		2.1	3	HBVDNA定量
(-)	2000.00	<		(-)	284.6			(-)		(-)	2.1	3	HBVDNA定量
(-)	2000.00	<		(-)	219.2			(-)		(-)	0.0	4	HBVDNA定量
(-)													HBcrAg　6.8<
(-)	2000.00	<		(-)	168.3			(-)	1.9				
(-)	2000.00	<		(-)	150.6			(-)	1.9		2.1	3	HBcrAg　6.8<
(-)	2000.00	<		(-)	122.8			(-)	1.6		0.0	4	
(-)	2000.00	<		(-)	102.8			(-)	1.4		0.0	4	
(-)	2000.00	<		(-)	81.4			(-)	1.4		0.0	4	
(-)	250.00	<		(-)	32.9			(-)	1.3		2.1	3	
(-)	132.85			(-)	20.3			(-)	1.1		0.0	4	
(-)	120.26			(-)	16.3			(-)		(-)	2.5	1	
(-)	97.00			(-)	15.2			(-)		(-)	0.0	4	
(-)	85.39			(-)	11.8			(-)		(-)	0.0	4	
(-)	73.72			(-)	10.4			(-)		(-)	0.0	4	
(-)	67.89			(-)	8.2			(-)		(-)	0.0	4	
(-)	50.10			(-)	6.6			(-)			0.0	4	
(-)	31.32			(-)	4.8			(-)			0.0	4	
(-)	19.37			(-)	2.2			(-)			0.0	4	
(-)	15.45			(-)	2.2			(-)			0.0	4	
(-)	13.78			(-)	2.0			(-)			0.0	4	
(-)	7.04			(-)	1.1			(-)			0.0	4	
(-)	0.06			(-)		(-)	76.0				0.0	4	
(-)		(-)		(-)		(-)	61.0				0.0	4	
(-)	0.10			(-)		(-)		(-)			0.0	4	
(-)	0.19			(-)	1.9			(-)			0.0	4	
(-)	0.59			(-)	7.7			(-)			0.0	4	
(-)	1.40			(-)	10.2			(-)			0.0	4	
(-)	1.79			(-)	8.0			(-)			0.0	4	
(-)	1.97			(-)	12.6			(-)			0.0	4	
(-)	1.70			(-)	5.0			(-)			0.0	4	

(-)	2.24			(-)	3.5			(-)			0.0	4	
(-)	2.51			(-)	3.1			(-)			0.0	4	
(-)	2.29			(-)	3.0			(-)			0.0	4	
(-)	2.17			(-)	2.4			(-)			0.0	4	
(-)	1.55			(-)	2.5			(-)			0.0	4	
14.3		(-)											
14.3		(-)	27.9						94.0				
14.3		(-)		(-)									
14.3		(-)	13.2						7.3				
14.3		(-)	12.9						6.4				
14.3		(-)		(-)					5.9				
14.3		(-)		(-)					5.9				
14.3		(-)		(-)					5.7				
14.3		(-)		(-)					6.2				
14.3		(-)		(-)					6.3				
14.3				(-)					7.4				
14.3				(-)					7.1				
14.3		(-)											
14.3				(-)					5.5				
(-)		(-)											
(-)		(-)	13.6						89.0				
(-)		(-)		(-)					7.2				
(-)		(-)		(-)									
(-)		(-)		(-)									
(-)		(-)		(-)									
(-)				(-)									
(-)				(-)									
(-)		(-)											
(-)		(-)		(-)					86.0				
(-)											0.0	4	HBVDNA定量
(-)		(-)											
(-)		(-)		(-)		(-)		(-)	7.0				
(-)		(-)		(-)					7.2				
(-)		(-)		(-)					5.9				
(-)									3.7				
(-)				(-)					4.4				
(-)		(-)							74.0				
(-)			5.2										
(-)		(-)	80.9							(-)			
(-)		(-)	25.3			(-)		(-)		(-)			
(-)		(-)	17.1							(-)			
(-)		(-)	30.8							(-)			
(-)		(-)	14.2							(-)			
(-)		(-)	15.3							(-)			
(-)		(-)	20.7							(-)			
(-)		(-)	20.5										
(-)			21.1							(-)			
(-)			17.1							(-)			
(-)		(-)	17.0										
(-)		(-)	16.9										
(-)										(-)	0.0	4	
(-)		(-)											
(-)		(-)											
(-)				(-)					98.0				
(-)		(-)	12.3			(-)		(-)	48.6		0.0	4	
(-)		(-)	9.6			(-)		(-)	44.7		**2.1**	1	
(-)		(-)	9.7			(-)		(-)	41.6		**2.1**	3	
(-)		(-)	7.9			(-)		(-)	37.4		0.0	4	
(-)		(-)		(-)		(-)		(-)	33.3		0.0	4	
(-)		(-)		(-)		(-)		(-)	35.5		0.0	4	
(-)		(-)											
(-)		(-)											
(-)		(-)		(-)					9.6		0.0	4	
(-)		(-)		(-)					7.9		0.0	4	
(-)		(-)		(-)		(-)	76.0		13400.0		3.7	3	HBV(TMA)
(-)		(-)		(-)					100.0				
(-)		(-)		(-)					100.0		0.0	4	
(-)		(-)	18.9						9.5		0.0	4	
(-)		(-)	43.8						8.7				
(-)		(-)	42.1						6.9				
(-)		(-)	47.1						7.8				
(-)			57.9						7.1				
(-)		(-)	1000.0	<					1.3				
(-)		(-)	424.8							(-)			
(-)		(-)	1000.0	<						(-)			
(-)		(-)	1000.0	<									
(-)		(-)	1000.0	<									
(-)			1000.0	<									
(-)			1000.0	<									

(-)		(-)	7.7						90.0				
(-)		(-)		(-)					15.1				
(-)		(-)		(-)					7.7				
(-)		(-)		(-)									
(-)				(-)									
(-)				(-)									
(-)				(-)									
(-)	995.90										2.6	3	HBVDNA-PCR
(-)		(-)	16.6						100.0		0.0	4	HBVDNA定量
(-)		(-)	16.3						10700.0		0.0	4	HBVDNA定量
(-)		(-)	6.6			(-)		(-)	208.4		0.0	4	HBcrAg　3.1
(-)		(-)		(-)		(-)		(-)	172.8		0.0	4	
(-)		(-)	15.6										
(-)		(-)	49.4						9.3				
(-)		(-)											
(-)		(-)	148.0						7.6		0.0	4	
(-)		(-)	140.5						8.5		0.0	4	
(-)			143.3						7.1				
(-)		(-)	96.4										
(-)		(-)											
(-)		(-)											
(-)		(-)		(-)		(-)		(-)	96.0				
(-)											0.0	4	HBVDNA定量
(-)											0.0	4	HBVDNA定量
(-)											0.0	4	HBVDNA定量、HBcrAg　3.0[illegible]
(-)		(-)		(-)					8.3				
(-)											0.0	4	
(-)		(-)											
(-)		(-)											
22.3											0.0	4	HBVDNA定量
22.3		(-)	9.3			(-)	93.0		90.0				
22.3		(-)	8.1						88.0				
22.3		(-)											
22.3		(-)		(-)					7.7				
22.3		(-)		(-)					6.8				
22.3		(-)		(-)					7.0				
22.3		(-)		(-)					6.6				
22.3		(-)		(-)					6.9				
22.3		(-)		(-)									
22.3		(-)		(-)									
22.3		(-)											
(-)		(-)											
(-)				(-)					10.3	(+)			HBc抗体S/CO
(-)											0.0	4	HBVDNA定量
(-)		(-)		(-)					92.0				
(-)		(-)		(-)		(-)			93.0				
(-)											0.0	4	HBVDNA定量
(-)		(-)		(-)					93.0				
(-)		(-)		(-)					9.3				
(-)		(-)		(-)					9.3				
(-)		(-)	34.2						7.7		0.0	4	
(-)		(-)	45.8						7.8				
(-)		(-)	31.2						7.8		0.0	4	
(-)		(-)											
(-)		(-)	226.0						7.8				
(-)		(-)											
(-)		(-)											
(-)		(-)		(-)		(-)		(-)	54.0				
(-)											0.0	4	HBVDNA定量
(-)		(-)		(-)						(-)	0.0	4	HBVDNA定量
(-)		(-)		(-)						(-)			
(-)				(-)									
(-)		(-)		(-)						(-)			
(-)				(-)						(-)			
(-)		(-)		(-)						(-)			
(-)				(-)									
(-)		(-)	1000.0	<					59.0				
(-)		(-)	762.4						1.1				
(-)		(-)	481.8						1.4				
(-)		(-)	680.9							(-)			
(-)		(-)	600.5										
(-)		(-)		(-)						(-)			
(-)			191.4										
(-)			132.5										
(-)			66.2										
(-)		(-)											
(-)			5.2							(-)			
(-)		(-)		(-)					1.1				
(-)		(-)		(-)					1.3				

(-)		(-)		(-)					1.5		0.0	4	
(-)		(-)		(-)					1.6				
(-)		(-)		(-)					1.3				
(-)		(-)		(-)					1.3				
(-)		(-)		(-)					1.1		0.0	4	
(-)		(-)		(-)					1.3		0.0	4	
(-)		(-)		(-)					1.2				
(-)		(-)											
(-)		(-)											
(-)		(-)											
(-)				(-)						(-)			
(-)				(-)						(-)			
(-)		(-)											
(-)		(-)		(-)					6.4				
(-)											2.1	3	
(-)				(-)					2.6		0.0	4	
(-)		(-)		(-)					2.4		0.0	4	
(-)		(-)	7.6			(-)		(-)	67.0				
(-)		(-)	8.2			(-)		(-)	61.0				
(-)		(-)	5.4						59.0				
(-)		(-)		(-)						(-)			
(-)		(-)											
(-)		(-)		(-)						(-)			
(-)		(-)		(-)						(-)			
(-)		(-)		(-)						(-)			
(-)		(-)											
(-)		(-)		(-)						(-)			
(-)		(-)		(-)						(-)			
(-)		(-)											
(-)				(-)						(-)			
(-)				(-)						(-)			
(-)		(-)		(-)					3.1				
(-)		(-)		(-)					2.6				
(-)		(-)		(-)									
(-)				(-)									
(-)				(-)									
(-)				(-)									
(-)		(-)		(-)									
(-)		(-)		(-)		(-)	96.0		90.0				
(-)		(-)		(-)					88.0				
(-)		(-)		(-)					86.0		0.0	4	HBVDNA定量
(-)				(-)					7.3				
(-)											0.0	4	
(-)		(-)		(-)					7.3				
(-)		(-)		(-)					4.4				
(-)		(-)											
(-)		(-)											
(-)		(-)											
(-)				(-)					4.3				
(-)											0.0	4	
(-)	2000.00	<		(-)	1600.0	<					7.6	2	HBVDNA-PCR
(-)											7.6	1	HBVDNA-PCR
(-)	2000.00	<		(-)	1600.0	<		(-)	96.0		3.4	1	HBVDNA-PCR
(-)	1775.50			(-)	2.4			(-)	97.0		2.6	3	HBVDNA-PCR
(-)		(-)											
(-)		(-)	8.6			(-)		(-)	97.0		0.0	4	HBVDNA定量、HBcrAg　3.1
(-)		(-)		(-)		(-)		(-)	9.6				
(-)		(-)	12.0			(-)		(-)	8.6				
(-)		(-)											
(-)		(-)		(-)					98.0				
(-)											0.0	4	HBVDNA定量
(-)											0.0	4	HBVDNA定量
(-)		(-)											
(-)		(-)		(-)		(-)	52.0		36.7				
(-)		(-)		(-)					7.4				
(-)		(-)		(-)					6.2		0.0	4	
(-)		(-)		(-)		(-)		(-)	6.5		0.0	4	HBcrAg　<3.0
(-)		(-)		(-)		(-)		(-)					HBcrAg　<3.0
(-)		(-)		(-)		(-)		(-)					HBcrAg　<3.0
(-)		(-)		(-)		(-)		(-)	6.4				HBcrAg　<3.0
(-)		(-)		(-)		(-)		(-)					
(-)		(-)											
(-)		(-)		(-)		(-)		(-)					HBcrAg　<3.0
(-)		(-)		(-)		(-)		(-)					HBcrAg　<3.0
(-)				(-)									
(-)		(-)		(-)						(-)			
(-)		(-)		(-)						(-)			
(-)		(-)	113.9						92.0				
(-)		(-)	210.2			(-)		(-)	86.0		2.6	3	HBVDNA-PCR

(-)		(-)	83.3			(-)		(-)	86.0		2.6	3	HBVDNA-PCR
(-)		(-)	187.9			(-)		(-)	85.0		2.6	3	HBVDNA-PCR
(-)		(-)	9.8			(-)		(-)	76.0				
(-)		(-)	22.6						73.0		0.0	4	HBVDNA定量
(-)		(-)	25.7							(-)			
(-)		(-)	33.3							(-)			
(-)		(-)	249.0							(-)			
(-)		(-)	349.0							(-)			
(-)		(-)	350.8							(-)			
(-)		(-)											
(-)											0.0	4	
(-)			140.5							(-)			
(-)		(-)											
92.1	2000.00	<		(-)					97.0				
92.1	2000.00	<		(-)	1600.0	<		(-)			7.7	2	HBVDNA-PCR
92.1	2000.00	<		(-)	1514.4			(-)			7.7	2	HBVDNA-PCR
92.1	100.00	<	10.0	>	1400.0	(+)	35.0	>			7.7	2	HBs抗原CLIA、HBs抗体CLIA、
92.1	100.00	<	10.0	>	1040.0	(+)	35.0	>			8.8	2	HBs抗原CLIA、HBs抗体CLIA、
92.1	2000.00	<		(-)	1600.0	<		(-)		(-)	8.8	2	HBVDNA定量
92.1											8.8	2	HBVDNA定量
92.1											7.4	1	HBVDNA定量
92.1											6.7	1	HBVDNA定量
92.1											5.7	1	HBVDNA定量
92.1											5.1	1	HBVDNA定量
92.1											4.5	1	HBVDNA定量
92.1											4.0	1	HBVDNA定量
92.1											3.7	1	HBVDNA定量
92.1											3.8	1	HBVDNA定量
92.1											3.5	1	HBVDNA定量
92.1											3.4	1	HBVDNA定量
92.1											3.4	1	HBVDNA定量
92.1											3.3	1	HBVDNA定量
92.1											3.1	1	HBVDNA定量
92.1	2000.00	<		(-)	1.4			(-)	11300.0		3.0	1	HBVDNA定量
92.1	2000.00	<		(-)		(-)		(-)	99.0		2.7	1	HBVDNA定量
92.1											2.4	1	HBVDNA定量
92.1	2000.00	<		(-)		(-)	78.0		300.0		0.0	4	
92.1	2000.00	<		(-)		(-)	73.0		273.9		2.1	3	
92.1											2.1	3	
92.1											2.1	3	
92.1	2000.00	<		(-)		(-)	66.0		265.7		0.0	4	
92.1	2000.00	<		(-)		(-)	74.0		278.9		2.1	3	HBcrAg　3.2
92.1	2000.00	<		(-)		(-)	70.0		278.7		2.1	3	HBcrAg　3.1
92.1	250.00	<		(-)		(-)	58.0		11.8		2.1	3	
92.1	250.00	<		(-)		(-)	70.0		11.0		0.0	4	
92.1	250.00	<		(-)		(-)	60.0		11.2		2.1	3	
92.1	250.00	<		(-)		(-)	68.0		11.8		0.0	4	
92.1	250.00	<		(-)		(-)	60.0		12.1		0.0	4	
92.1	250.00	<		(-)		(-)	71.0		9.9		0.0	4	
92.1	250.00	<		(-)		(-)	69.0		10.0		0.0	4	
92.1	250.00	<		(-)		(-)	69.0		10.4		0.0	4	
92.1	250.00	<		(-)		(-)	79.0		9.9		0.0	4	
92.1	250.00	<		(-)					8.9				
92.1	250.00	<		(-)									
92.1											0.0	4	
92.1	250.00	<		(-)							0.0	4	
92.1	250.00	<		(-)		(-)	71.0				0.0	4	
92.1	250.00	<		(-)		(-)	67.0				0.0	4	
92.1	250.00	<		(-)		(-)	60.0				0.0	4	
92.1	250.00	<		(-)		(-)	58.0				0.0	4	
92.1											1.0	3	
92.1	250.00	<											
92.1											8.9	1	
(-)	7.80			(-)					99.0				
(-)	6.30												
(-)	6.70			(-)		(-)		(-)	99.0		3.7	1	HBVDNA定量
(-)	5.40			(-)		(-)		(-)	99.0		2.1	3	HBVDNA定量
(-)											0.0	4	HBVDNA定量
(-)	1.50												
(-)	0.39			(-)		(-)		(-)	8.2				
(-)	0.45												
(-)		(-)		(-)							0.0	4	
(-)	0.07			(-)					8.8				
(-)											0.0	4	
(-)		(-)		(-)					7.9				
(-)		(-)		(-)					7.6				
(-)		(-)		(-)					6.4		2.1	3	
(-)		(-)		(-)		(-)		(-)			2.1	3	
(-)											0.0	4	

(-)		(-)		(-)					6.5				
(-)											0.0	4	
(-)											0.0	4	
(-)				(-)									
(-)		(-)											
(-)				(-)					5.0				
(-)		(-)	935.5						53.0				
(-)		(-)	1000.0	<		(-)		(-)		(-)			
(-)		(-)	664.0										
(-)			780.6										
(-)			782.2										
(-)			923.9										
(-)	135.80			(-)	2.0			(-)		(-)	5.5	1	HBVDNA-PCR、IgM-HBc抗体
(-)	2000.00	<		(-)	795.1			(-)	88.0				
(-)	2000.00	<	5.4		1458.2			(-)	18160.0		7.7	2	HBVDNA-PCR
(-)			6.7										
(-)	2000.00	<			767.2						7.4	1	HBVDNA-PCR
(-)	2000.00	<		(-)	1600.0	<					7.7	2	HBVDNA-PCR
(-)	2000.00	<		(-)	1600.0	<		(-)	16920.0				
(-)											7.7	2	HBVDNA-PCR
(-)	2000.00	<		(-)	1600.0	<		(-)	98.0		7.7	2	HBVDNA-PCR
(-)	2000.00	<		(-)					92.0		4.5	1	HBVDNA定量
(-)	2000.00	<		(-)					99.0		3.4	1	HBVDNA定量
(-)	2000.00	<		(-)					99.0		3.0	1	HBVDNA定量
(-)	2000.00	<		(-)					98.0		3.0	1	HBVDNA定量
(-)											2.6	1	HBVDNA定量
(-)											2.4	1	HBVDNA定量
(-)											2.2	1	HBVDNA定量
(-)											2.1	3	HBVDNA定量
(-)		(-)		(-)					99.0		2.1	3	HBVDNA定量
(-)											2.1	3	HBVDNA定量
(-)											0.0	4	HBVDNA定量
(-)		(-)	30.2						99.0		0.0	4	HBVDNA定量
(-)		(-)	44.8						99.0		0.0	4	HBVDNA定量
(-)		(-)	37.5						51.5				HBcrAg　3.6
(-)		(-)		(-)					56.4				
(-)		(-)		(-)					39.5				HBcrAg　3.5
(-)											0.0	4	
(-)													HBcrAg　3.6
(-)											0.0	4	
(-)		(-)		(-)		(-)		(-)	8.7				
(-)		(-)		(-)		(-)		(-)	8.3				
(-)		(-)		(-)		(-)		(-)	7.7				
(-)		(-)		(-)		(-)		(-)	6.1				
(-)		(-)		(-)					6.8				
(-)		(-)		(-)					5.2				
(-)		(-)											
(-)		(-)											
(-)											0.0	4	
(-)				(-)					5.1				
(-)		(-)											
(-)				(-)									
(-)		(-)											
(-)											1.0	3	
(-)											0.0	4	
(-)		(-)	64.2										
(-)				(-)					4.1				
(-)		(-)		(-)					2.2				
(-)		(-)		(-)					2.0				
(-)		(-)		(-)					1.6				
(-)		(-)		(-)					1.7				
(-)		(-)		(-)					1.6				
(-)			8.0	>									HBs抗体[PHA]、HBcrAg　<3
(-)		(-)											
(-)				(-)					1.2				
(-)		(-)											
(-)		(-)		(-)		(-)	97.0		100.0				
(-)		(-)		(-)					99.0				
(-)											0.0	4	HBVDNA定量
(-)											0.0	4	HBVDNA定量
(-)		(-)											
(-)		(-)		(-)					48.1				
(-)											0.0	4	
(-)											0.0	4	
(-)											0.0	4	
(-)		(-)		(-)					7.6				
(-)		(-)		(-)		(-)	88.0		7.2		0.0	4	
(-)		(-)		(-)					5.2				
(-)		(-)											

(-)		(-)		(-)					98.0				
(-)		(-)	7.5						98.0				
(-)		(-)		(-)					97.0				
(-)		(-)		(-)					97.0				
(-)			93.0										
(-)			192.8						98.0				
(-)		(-)	178.5										
(-)		(-)	148.7						53.9				
(-)		(-)	75.7						10.0				
(-)		(-)	52.8						9.9				
(-)		(-)	52.6						9.4				
(-)		(-)	73.0						9.4				
(-)		(-)	70.2						7.1				
(-)		(-)											
(-)			67.9										
(-)			60.5										
(-)		(-)											
(-)		(-)		(-)					94.0				
(-)											0.0	4	HBVDNA定量
(-)											0.0	4	HBVDNA定量
(-)		(-)		(-)					45.3				
(-)			11.7						7.5				
(-)			10.3										
(-)		(-)	21.8						10.6				
(-)		(-)		(-)					9.4				
(-)											0.0	4	
(-)		(-)		(-)					9.3				
(-)		(-)	19.5						9.2				
(-)		(-)											
(-)	2000.00	<		(-)					99.0				
(-)					1600.0	<		(-)					
(-)											7.7	2	HBVDNA-PCR
(-)	2000.00	<		(-)	1600.0	<		(-)	99.0		8.8	2	HBV(TMA)
(-)											8.8	2	HBVDNA定量
(-)											8.8	2	HBVDNA定量
(-)											8.8	2	HBVDNA定量
(-)	2000.00	<		(-)	1600.0	<		(-)			5.9	1	HBVDNA定量
(-)	2000.00	<		(-)	1600.0	<		(-)			5.5	1	HBVDNA定量
(-)											5.1	1	HBVDNA定量
(-)	2000.00	<		(-)	1600.0	<		(-)			3.8	1	HBVDNA定量
(-)											3.2	1	HBVDNA定量
(-)											3.1	1	HBVDNA定量
(-)											3.2	1	HBVDNA定量
(-)											2.8	1	HBVDNA定量
(-)											2.6	1	HBVDNA定量
(-)											2.1	3	HBVDNA定量
(-)	2000.00	<		(-)	269.5			(-)			2.1	3	HBVDNA定量
(-)											0.0	4	HBVDNA定量
(-)	2000.00	<		(-)	152.8			(-)			2.1	3	HBVDNA定量
(-)											0.0	4	HBVDNA定量
(-)											0.0	4	HBVDNA定量
(-)											2.1	3	HBVDNA定量
(-)											0.0	4	HBVDNA定量
(-)											0.0	4	HBVDNA定量
(-)											0.0	4	HBVDNA定量
(-)		(-)	7.9			(-)		(-)	99.0		0.0	4	HBVDNA定量、HBcrAg　3.7
(-)											0.0	4	HBVDNA定量
(-)											0.0	4	HBVDNA定量
(-)											0.0	4	HBVDNA定量
(-)											0.0	4	HBVDNA定量
(-)											0.0	4	HBVDNA定量
(-)		(-)		(-)		(-)		(-)	98.0		0.0	4	HBVDNA定量、HBcrAg　3.5
(-)											0.0	4	HBVDNA定量
(-)											0.0	4	HBVDNA定量
(-)											0.0	4	HBVDNA定量
(-)		(-)		(-)		(-)		(-)	98.0		0.0	4	HBVDNA定量
(-)											0.0	4	HBVDNA定量
(-)											0.0	4	HBVDNA定量
(-)		(-)		(-)		(-)		(-)	98.0		0.0	4	HBVDNA定量
(-)											0.0	4	HBVDNA定量
(-)											0.0	4	HBVDNA定量
(-)		(-)		(-)		(-)		(-)	98.0		0.0	4	
(-)													HBcrAg　3.4
(-)		(-)	8.9			(-)		(-)	65.1		0.0	4	
(-)											2.1	3	HBcrAg　3.3
(-)		(-)	14.4			(-)		(-)	62.8		0.0	4	
(-)													HBcrAg　3.3
(-)											0.0	4	
(-)		(-)	10.5			(-)		(-)	65.1		0.0	4	

(-)													HBcrAg　3.4
(-)		(-)	5.8			(-)		(-)	58.4		0.0	4	
(-)											0.0	4	
(-)		(-)	7.6			(-)		(-)	55.8		0.0	4	HBcrAg　3.2
(-)											0.0	4	
(-)		(-)	37.3			(-)		(-)			0.0	4	
(-)											0.0	4	
(-)		(-)	38.5			(-)		(-)	52.6		0.0	4	
(-)											2.1	3	
(-)		(-)	23.8			(-)		(-)	10.0		2.1	3	
(-)											0.0	4	
(-)											0.0	4	
(-)											0.0	4	
(-)		(-)	23.8			(-)		(-)	9.5		0.0	4	
(-)		(-)	23.0			(-)		(-)	8.8		0.0	4	
(-)		(-)	29.4			(-)		(-)	10.0		0.0	4	
(-)		(-)	24.7			(-)		(-)	9.2		0.0	4	
(-)		(-)	12.1						8.9		0.0	4	
(-)		(-)		(-)					8.8		2.1	3	
(-)		(-)		(-)					8.8		0.0	4	
(-)		(-)	10.1			(-)		(-)	8.1				
(-)		(-)		(-)					8.3		0.0	4	
(-)											0.0	4	
(-)				(-)					6.5				
(-)		(-)		(-)		(-)	62.0		98.0				
(-)		(-)		(-)		(-)	60.0		97.0				
(-)		(-)		(-)		(-)		(-)	95.0				
(-)		(-)		(-)		(-)		(-)	93.0		1.8	3	HBVDNA定量
(-)		(-)		(-)		(-)		(-)	18.0				
(-)		(-)	10.0						9.7				
(-)		(-)		(-)					9.9				
(-)		(-)											
		(-)	493.6						65.0				
		(-)	354.6						52.0				
		(-)	1000.0	<					2.0				
		(-)	344.7						1.4				
		(-)	63.7						1.0				
											0.0	4	
		(-)											
			76.5										
										(-)			
(-)		(-)		(-)						(-)			
(-)		(-)	913.3							(-)			
(-)		(-)	73.1						71.0		0.0	4	HBVDNA定量
(-)		(-)	61.1						1.6				
(-)		(-)	52.2						1.1				
(-)		(-)	44.4						1.0				
(-)			75.9										
(-)										(-)			
(-)			65.5										
21.8		(-)		(-)					66.0				
21.8		(-)		(-)		(-)		(-)	61.0		**2.6**	3	HBVDNA-PCR、HBV(TMA)
21.8		(-)	7.6			(-)		(-)	60.0		**1.8**	3	HBVDNA定量
21.8		(-)	7.3							(-)			
21.8													HBcrAg　<3.0
21.8		(-)	15.2						6.4				
21.8		(-)	11.4						5.9				
21.8		(-)		(-)					5.7				
21.8				(-)					4.6				
21.8		(-)		(-)							0.0	4	
(-)		(-)	19.0						82.0				
(-)		(-)	10.3						5.8				
(-)		(-)	13.8						4.5				
(-)		(-)	11.8						4.0				
(-)		(-)		(-)					3.9				
(-)		(-)		(-)					3.3				
(-)		(-)	44.4						2.9				
(-)				(-)									
100<		(-)	10.3										
100<		(-)											
100<		(-)		(-)					10.6				
100<		(-)		(-)		(-)		(-)	7.2				
100<		(-)		(-)					4.3		0.0	4	
100<		(-)											
100<				(-)					4.4				
(-)	2000.00	<	7.5		1600.0	<		(-)	94.0				
(-)	2000.00	<	7.2		1600.0	<		(-)			7.7	2	HBVDNA-PCR
(-)											7.7	2	HBVDNA-PCR
(-)											7.7	2	HBVDNA-PCR

(-)											8.8	2	HBV(TMA)
(-)											8.8	2	HBV(TMA)
(-)											7.2	1	HBV(TMA)
(-)	2000.00	<		(-)	1600.0	<		(-)			6.2	1	HBVDNA-PCR
(-)	2000.00	<		(-)	1600.0	<		(-)			5.5	1	HBVDNA-PCR
(-)	2000.00	<		(-)	1600.0	<		(-)			5.3	1	HBVDNA定量
(-)											4.4	1	HBVDNA定量
(-)	2000.00	<		(-)	1600.0	<		(-)			4.2	1	HBVDNA定量
(-)	2000.00	<		(-)	1600.0	<		(-)			3.8	1	HBVDNA定量
(-)	2000.00	<		(-)	1600.0	<		(-)			3.8	1	HBVDNA定量
(-)											3.5	1	HBVDNA定量
(-)											3.3	1	HBVDNA定量
(-)											3.1	1	HBVDNA定量
(-)											3.1	1	HBVDNA定量
(-)											2.8	1	HBVDNA定量
(-)											2.3	1	HBVDNA定量
(-)											2.8	1	HBVDNA定量
(-)											2.8	1	HBVDNA定量
(-)											2.2	1	HBVDNA定量
(-)											2.4	1	HBVDNA定量
(-)											2.1	3	HBVDNA定量
(-)											2.1	3	HBVDNA定量
(-)											2.1	1	HBVDNA定量
(-)											2.2	1	HBVDNA定量
(-)											2.1	3	HBVDNA定量
(-)											0.0	4	HBVDNA定量
(-)		(-)		(-)		(-)		(-)	100.0		0.0	4	HBVDNA定量
(-)		(-)		(-)		(-)		(-)	99.0		2.1	3	
(-)											2.1	3	
(-)		(-)		(-)		(-)		(-)	117.2		2.1	3	
(-)											0.0	4	
(-)											2.1	3	
(-)		(-)		(-)		(-)		(-)	92.9		0.0	4	
(-)											2.1	3	
(-)											0.0	4	
(-)		(-)		(-)		(-)		(-)	86.8		0.0	4	HBcrAg 3.6
(-)											0.0	4	
(-)											0.0	4	
(-)											0.0	4	
(-)	0.06			(-)		(-)		(-)	11.0		0.0	4	
(-)		(-)		(-)		(-)		(-)	10.2		2.1	3	
(-)		(-)		(-)					10.5		0.0	4	
(-)		(-)		(-)					9.4				
(-)		(-)		(-)		(-)		(-)	9.3		0.0	4	
(-)		(-)		(-)		(-)		(-)	9.6		0.0	4	
(-)											0.0	4	
(-)											0.0	4	
(-)													HBcrAg 3
(-)											0.0	4	
(-)	0.06			(-)					7.3		0.0	4	
(-)	0.09			(-)					7.9				
(-)				(-)					8.2		0.0	4	
(-)				(-)					8.5		0.0	4	
(-)				(-)					8.1		0.0	4	
(-)				(-)					9.2		0.0	4	
(-)				(-)					8.3		0.0	4	
(-)	0.05			(-)							0.0	4	
(-)	0.05			(-)							0.0	4	
(-)		(-)		(-)							0.0	4	
(-)				(-)					7.1		0.0	4	
(-)									6.8		0.0	4	
(-)									6.3		0.0	4	
(-)	2000.00	<		(-)					17000.0				
(-)	389.20												
(-)	20.60			(-)	1.4			(-)	12660.0		1.8	3	HBVDNA定量
(-)		(-)		(-)					99.0		0.0	4	HBVDNA定量
(-)		(-)	26.0			(-)		(-)	133.5		0.0	4	
(-)		(-)		(-)		(-)		(-)	11.8		0.0	4	
(-)		(-)	34.0						10.4				
(-)		(-)	34.0						8.0				
(-)			22.8										
(-)		(-)		(-)				(-)					
(-)			61.8										
(-)			39.0						11.5				
(-)		(-)	11.4										
(-)			11.0										
(-)				(-)									
(-)				(-)									
(-)		(-)	24.2					(-)					

(-)		(-)	27.5					(-)					
(-)		(-)	18.0						16.7				
(-)		(-)											
(-)				(-)					6.2				
(-)		(-)											
(-)		(-)											
(-)											0.0	4	
(-)											0.0	4	
(-)											0.0	4	
(-)											0.0	4	
(-)											0.0	4	
(-)											0.0	4	
(-)											0.0	4	
(-)											0.0	4	
(-)											0.0	4	
(-)											0.0	4	
(-)											0.0	4	
(-)											0.0	4	
(-)											0.0	4	
(-)											0.0	4	
(-)											0.0	4	
(-)											0.0	4	
(-)											0.0	4	
(-)											0.0	4	
(-)											0.0	4	
(-)											0.0	4	
(-)											0.0	4	
(-)											0.0	4	
(-)											0.0	4	
(-)											0.0	4	
(-)											0.0	4	
(-)											0.0	4	
(-)											0.0	4	
(-)											0.0	4	
		(-)	76.0						57.0				
		(-)	41.5										
		(-)											
											0.0	4	
		(-)	10.5							(-)			
											0.0	4	
											0.0	4	
		(-)		(-)						(-)			
(-)		(-)	125.3					(-)					
(-)		(-)	68.4						70.0		0.0	4	HBVDNA定量
(-)		(-)		(-)									
(-)		(-)	10.2										
(-)			11.1										
(-)				(-)									
(-)		(-)	49.4			(-)	99.0		99.0				
(-)		(-)		(-)					11.1				
(-)		(-)		(-)					9.5				
(-)				(-)					9.3				
(-)				(-)					9.3				
(-)		(-)		(-)									
(-)		(-)		(-)									
23.8	2000.00	<		(-)					15260.0				
23.8	2000.00	<		(-)	1600.0	<		(-)	15400.0		7.7	2	HBVDNA-PCR
23.8											8.8	2	HBVDNA定量
23.8	2000.00	<		(-)	1600.0	<		(-)	18000.0		8.8	2	HBVDNA定量
23.8											8.8	2	HBVDNA定量
23.8											5.1	1	HBVDNA定量
23.8											4.2	1	HBVDNA定量
23.8	2000.00	<		(-)	28.7			(-)	18000.0		3.3	1	HBVDNA定量
23.8	2000.00	<		(-)	18.7			(-)	18000.0		2.9	1	HBVDNA定量
23.8											2.7	1	HBVDNA定量
23.8											2.4	1	HBVDNA定量
23.8											2.1	3	HBVDNA定量
23.8											2.3	1	HBVDNA定量
23.8											2.1	3	HBVDNA定量
23.8											2.1	3	HBVDNA定量
23.8	2000.00	<		(-)	3.0			(-)	98.0		2.1	3	HBVDNA定量、HBcrAg　5.0
23.8	2000.00	<		(-)	3.1			(-)	98.0		2.1	3	HBVDNA定量、HBcrAg　4.9
23.8											0.0	4	
23.8											0.0	4	
23.8	2000.00	<		(-)	2.7			(-)	197.8		2.1	3	
23.8											0.0	4	
23.8	2000.00	<		(-)	1.6			(-)	166.4		2.1	3	
23.8	2000.00	<		(-)	1.4			(-)	147.7		2.1	3	
23.8	2000.00	<		(-)		(-)		(-)	168.2		2.1	3	

23.8	250.00	<		(-)	(-)		(-)	10.7		2.1	3	
23.8	250.00	<		(-)	(-)		(-)	10.0		0.0	4	
23.8	123.98			(-)	(-)		(-)	10.2		0.0	4	
23.8	186.79			(-)	(-)		(-)	9.1		0.0	4	
23.8	201.81			(-)				8.3		0.0	4	
23.8	144.32			(-)	(-)		(-)	8.2		2.1	3	
23.8	107.08			(-)	(-)		(-)	8.0		0.0	4	
23.8	16.15			(-)			(-)			0.0	4	
23.8	10.78											
23.8	13.29											
23.8	4.08											
23.8	4.70											
23.8		(-)								0.0	4	
23.8		(-)								0.0	4	
23.8					(-)		(-)			0.0	4	
23.8					(-)		(-)			0.0	4	
23.8		(-)		(-)				4.6		0.0	4	
23.8		(-)		(-)				5.1		0.0	4	
23.8		(-)		(-)						0.0	4	
23.8		(-)		(-)						0.0	4	
(-)		(-)	54.0						(-)			
(-)		(-)	483.5		(-)		(-)	61.0				
(-)		(-)	223.3		(-)		(-)	80.0		0.0	4	HBVDNA定量
(-)										0.0	4	HBVDNA定量
(-)										0.0	4	HBVDNA定量
(-)												HBcrAg　3.0ﾐﾏﾝ
(-)		(-)	194.3					6.9				
(-)		(-)	350.0					7.9				
(-)		(-)	751.0					6.1				
(-)		(-)		(-)				93.0				
(-)										0.0	4	HBVDNA定量
(-)										0.0	4	HBVDNA定量
(-)		(-)		(-)				95.0		0.0	4	HBVDNA定量
(-)										0.0	4	HBVDNA定量
(-)		(-)		(-)	(-)	51.0		94.0		0.0	4	HBVDNA定量
(-)		(-)		(-)	(-)		(-)	94.0		0.0	4	HBVDNA定量
(-)		(-)		(-)	(-)	52.0		22.2		0.0	4	HBcrAg　3.0ﾐﾏﾝ
(-)										0.0	4	
(-)		(-)		(-)	(-)	61.0		21.9		0.0	4	
(-)		(-)		(-)	(-)	63.0		19.8		0.0	4	HBcrAg　3.0ﾐﾏﾝ
(-)										0.0	4	
(-)		(-)		(-)				20.1		0.0	4	
(-)		(-)		(-)	(-)	79.0		9.6		0.0	4	
(-)		(-)		(-)	(-)	81.0		9.8		0.0	4	HBcrAg　<3.0
(-)		(-)		(-)				10.8				
(-)		(-)		(-)				8.1		0.0	4	
(-)		(-)		(-)	(-)	76.0		9.4		0.0	4	
(-)										0.0	4	
(-)										0.0	4	
(-)												HBcrAg　<3.0
(-)		(-)		(-)				6.3				
(-)				(-)				7.5				
(-)				(-)				8.0				
30.3		(-)		(-)					(-)			
30.3			292.7					92.0				
30.3		(-)	51.5		(-)		(-)	83.0		0.0	4	HBVDNA定量
30.3		(-)	43.0					81.0				
30.3			50.9					74.0				
30.3		(-)	25.9					1.4				
30.3		(-)	23.6					1.3				
30.3		(-)	24.3					1.1				
30.3		(-)	17.1					1.3				
30.3		(-)	23.3					1.0				
30.3		(-)	44.5						(-)			
30.3			37.9									
30.3			59.5						(-)			
(-)		(-)	1000.0	<				100.0				
(-)		(-)	190.0		(-)	100.0			(-)			
(-)		(-)	1000.0	<				10.3				
(-)		(-)	1000.0	<				8.5				
(-)		(-)	1000.0	<				8.6				
(-)			1000.0	<				8.7				
(-)			1000.0	<				7.3				
		(-)	223.3					97.0				
		(-)	204.7					99.0				
		(-)		(-)				11.1				
		(-)		(-)				10.8				
		(-)		(-)				7.9				
(-)		(-)										

(-)									**3.7**	3	HBV(TMA)
(-)	(-)	1000.0	<				56.0				
(-)	(-)	920.2						(-)			
(-)	(-)	828.0						(-)			
(-)	(-)	594.9						(-)			
(-)	(-)	939.6									
(-)	(-)										
(-)		1000.0	<					(-)			
(-)		889.4						(-)			
(-)	(-)		(-)				8.2				
(-)									0.0	4	
(-)	(-)	23.5					6.5				
(-)			(-)				6.9				
(-)	(-)		(-)				11800.0				
(-)	(-)		(-)	(-)	97.0		11800.0		0.0	4	HBVDNA定量
(-)	(-)		(-)	(-)	97.0		12780.0				
(-)	(-)		(-)	(-)	99.0		12020.0		0.0	4	HBVDNA定量
(-)	(-)		(-)	(-)	98.0		10780.0		0.0	4	HBVDNA定量
(-)	(-)		(-)	(-)	97.0		10800.0		0.0	4	HBVDNA定量
(-)	(-)		(-)								
(-)			(-)								
(-)			(-)								
(-)	(-)										
(-)	(-)	23.5					65.0				
(-)	(-)		(-)					(-)			
(-)	(-)		(-)					(-)			
(-)	(-)										
(-)	(-)		(-)					(-)			
(-)	(-)										
(-)	(-)										
(-)	(-)		(-)				95.0				
(-)	(-)		(-)				95.0		0.0	4	HBVDNA定量
(-)	(-)		(-)				9.1				
(-)									0.0	4	IgM-HBc抗体　0.18　ｲﾝｾｲ
(-)	(-)						8.1				
(-)			(-)								
(-)							7.1				
(-)			(-)								
(-)			(-)				5.9				
(-)			(-)				5.5				
	(-)	1000.0	<				80.0				
	(-)	149.6					1.2				
	(-)	127.3					1.0				
	(-)	169.2						(-)			
	(-)	105.8						(-)	0.0	4	
		85.3						(-)			
(-)	(-)	559.9						(-)			
(-)	(-)	424.2					2.0				
(-)	(-)	30.2					7.3				
(-)	(-)	12.7					7.9				
(-)	(-)	10.4					8.0				
(-)	(-)		(-)				6.9				
(-)	(-)		(-)				7.2				
(-)			(-)				6.8				
(-)			(-)				5.0				
(-)	(-)	174.9		(-)	**63.0**			(-)			
(-)	(-)										
(-)		76.9						(-)			
(-)	(-)	354.0									
(-)	(-)	478.0									
(-)		421.1									
(-)	(-)	473.7						(-)			
(-)									0.0	4	
(-)		513.6									
(-)	(-)										
(-)									0.0	4	
(-)									0.0	4	
(-)									0.0	4	
(-)									0.0	4	
(-)	(-)	75.0					58.0				
(-)	(-)										
(-)	(-)	65.2						(-)			
(-)	(-)	406.8						(-)			
(-)	(-)	162.9						(-)			
(-)	(-)	147.4						(-)			
(-)	(-)										
(-)	(-)		(-)					(-)			
(-)	(-)		(-)	(-)		(-)		(-)			
(-)			(-)								

(-)				(-)									
(-)		(-)		(-)					1.4				
(-)		(-)		(-)									
(-)		(-)							1.1				
(-)		(-)		(-)									
(-)		(-)		(-)					95.0				
(-)		(-)									0.0	4	HBVDNA定量
(-)		(-)	8.4						19.9				
(-)		(-)		(-)					14.6				
(-)		(-)	10.5						8.8				
(-)		(-)		(-)					8.4				
(-)											0.0	4	
(-)				(-)									
(-)		(-)				(-)			6.3		0.0	4	
(-)		(-)		(-)					5.2				
(-)		(-)											
11.1		(-)	22.3						78.0				
11.1		(-)		(-)					1.7				
11.1		(-)		(-)					2.0				
11.1		(-)		(-)					1.6				
11.1		(-)		(-)									
11.1		(-)		(-)									IgM-HBc抗体　0.06　ｲﾝｾｲ
11.1				(-)					1.3				
11.1		(-)											
11.1		(-)		(-)									
(-)		(-)		(-)		(-)		(-)	99.0		3.0	1	HBVDNA定量
(-)											3.2	1	HBVDNA定量
(-)											0.0	4	HBVDNA定量
(-)											0.0	4	HBVDNA定量
(-)				(-)									
(-)											2.1	3	HBVDNA定量
(-)		(-)		(-)		(-)		(-)	100.0		2.1	1	HBVDNA定量
(-)											0.0	4	HBVDNA定量
(-)											0.0	4	HBVDNA定量
(-)											0.0	4	HBVDNA定量
(-)											0.0	4	HBVDNA定量
(-)											0.0	4	HBVDNA定量
(-)		(-)		(-)		(-)		(-)	99.0		0.0	4	HBVDNA定量
(-)		(-)		(-)		(-)		(-)	99.0		0.0		HBVDNA定量
(-)		(-)		(-)		(-)		(-)	99.0		0.0	4	HBVDNA定量、HBcrAg　3.0ﾐ
(-)		(-)		(-)		(-)		(-)	121.3		0.0	4	HBcrAg　3.0ﾐﾏﾝ
(-)	0.06			(-)					11.7		0.0	4	
(-)													HBcrAg　<3.0
(-)		(-)		(-)		(-)		(-)	10.5		0.0	4	
(-)													HBcrAg　<3.0
(-)		(-)		(-)		(-)		(-)	11.3		0.0	4	
(-)													HBcrAg　<3.0
(-)		(-)		(-)					10.0				
(-)		(-)		(-)		(-)		(-)	11.3				
(-)		(-)		(-)					7.5				
(-)											0.0	4	
(-)		(-)		(-)					9.7				
(-)											0.0	4	
(-)		(-)		(-)									
(-)				(-)					9.8				
(-)				(-)					6.7				
(-)				(-)					7.0				
(-)	2000.00	<		(-)					97.0		9.1	2	HBVDNA定量
(-)					1600.0	<		(-)					
(-)											5.5	1	HBVDNA定量
(-)	2000.00	<		(-)	31.2			(-)	18660.0		3.3	1	HBVDNA定量
(-)											3.0	1	HBVDNA定量
(-)											2.9	1	HBVDNA定量
(-)											2.6	1	HBVDNA定量
(-)											2.3	1	HBVDNA定量
(-)											2.3	1	HBVDNA定量
(-)											2.1	3	HBVDNA定量
(-)											2.1	3	HBVDNA定量
(-)		(-)		(-)	5.1			(-)	99.0		2.1	3	HBVDNA定量
(-)											2.1	3	HBVDNA定量
(-)		(-)	11.1		4.0			(-)	13640.0		2.1	3	HBVDNA定量
(-)		(-)	34.6		1.5			(-)	17680.0		2.1	3	HBVDNA定量
(-)											0.0	4	HBVDNA定量
(-)		(-)	7.1			(-)		(-)	17720.0		0.0	4	HBVDNA定量
(-)											2.1	3	HBVDNA定量
(-)											0.0	4	HBVDNA定量
(-)		(-)	5.5			(-)		(-)	14660.0		2.1	3	
(-)											0.0	4	
(-)		(-)	11.2			(-)		(-)	133.2		0.0	4	

(-)									2.1	3	
(-)									0.0	4	
(-)									2.1	3	
(-)									2.1	3	
(-)	(-)		(-)	(-)			239.6		0.0	4	
(-)									0.0	4	
(-)	(-)	29.8					12.0		0.0	4	
(-)	(-)	10.1		(-)		(-)			0.0	4	
(-)			(-)				10.0		0.0	4	
(-)			(-)				10.3		0.0	4	
(-)	(-)		(-)				10.8		0.0	4	
(-)	(-)						9.8				
(-)	(-)	27.5					8.2		0.0	4	
(-)	(-)	30.5					7.7		0.0	4	
(-)		32.6					7.6				
(-)									0.0	4	
(-)									0.0	4	
(-)	(-)										
(-)	(-)										
(-)	(-)		(-)				86.0				
(-)	(-)	7.5					73.0				
(-)	(-)		(-)				2.9				
(-)	(-)	23.4									
(-)			(-)								
(-)			(-)								
(-)	(-)										
(-)	(-)										
(-)	(-)	11.5					97.0				
(-)									**2.1**	3	HBVDNA定量
(-)	(-)		(-)				6.5				
(-)	(-)		(-)								
(-)			(-)								
(-)			(-)								
(-)			(-)								
(-)	(-)		(-)					(-)			
(-)			(-)				55.0				
(-)			(-)					(-)			
(-)									0.0	4	
(-)	(-)										
(-)	(-)	10.0	>				1.0	>			HBs抗体CLIA、HBc抗体S/CO
(-)	(-)		(-)					(-)			
(-)	(-)		(-)					(-)			
(-)	(-)							(-)			
(-)	(-)		(-)								
(-)	(-)	46.4					12280.0				
(-)	(-)		(-)				117.9				
(-)									0.0	4	
(-)	(-)	13.8		(-)	60.0		9.7				
(-)	(-)	14.3					8.9				
(-)	(-)	16.9					6.9				
(-)	(-)	16.0									
(-)	(-)	30.6									
(-)	(-)		(-)				56.0				
(-)									0.0	4	HBVDNA定量
(-)	(-)		(-)	(-)		(-)		(-)			
(-)	(-)		(-)					(-)			
(-)	(-)		(-)								
(-)	(-)		(-)					(-)			
(-)			(-)								
(-)	(-)		(-)								
(-)			(-)					(-)			
(-)			(-)					(-)			
(-)			(-)					(-)			
(-)			(-)					(-)			
2.1	(-)										
2.1	(-)	12.2					60.0				
2.1		463.5					1.0				
2.1	(-)	522.7					1.1				
2.1	(-)	281.3						(-)			
2.1	(-)	591.3						(-)			
2.1	(-)										
2.1	(-)										
2.1	(-)										
(-)	(-)										
(-)			(-)				59.0				
(-)									0.0	4	HBVDNA定量
(-)	(-)		(-)	(-)		(-)	63.0		0.0	4	HBVDNA定量
(-)	(-)		(-)					(-)	0.0	4	HBVDNA定量
(-)	(-)		(-)					(-)			

(-)		(-)		(-)									
(-)		(-)		(-)						(-)			
(-)		(-)											
(-)		(-)											
(-)				(-)									
(-)				(-)									
(-)				(-)						(-)			
(-)				(-)									
(-)		(-)		(-)					91.0				
(-)											0.0	4	HBVDNA定量
(-)											0.0	4	HBVDNA定量
(-)		(-)		(-)					10.3		0.0	4	
(-)		(-)		(-)					10.2				
(-)		(-)	17.7			(-)		(-)	7.8				
(-)		(-)							7.6				
(-)		(-)	10.6						5.5				
(-)		(-)											
(-)		(-)		(-)					6.0				
(-)											0.0	4	
(-)		(-)											
(-)		(-)		(-)					6.9				
(-)		(-)		(-)					5.9		0.0	4	
(-)		(-)											
(-)		(-)	31.7						92.0				
(-)		(-)		(-)					2.2				
(-)											**2.1**	3	
(-)		(-)		(-)					1.7				
(-)											0.0	4	
(-)		(-)	134.2						1.4		0.0	4	
(-)											0.0	4	
(-)											0.0	4	
(-)											0.0	4	
(-)											0.0	4	
(-)											0.0	4	
(-)											0.0	4	
(-)											0.0	4	
(-)											0.0	4	
(-)											0.0	4	
(-)											0.0	4	
(-)											0.0	4	
(-)											0.0	4	
(-)		(-)	256.0	=<									HBs抗体[PHA]
(-)													HBcrAg　<3.0
(-)		(-)	286.6						1.2				
(-)													HBcrAg　<3.0
(-)			114.7							(-)			
(-)		(-)											
(-)			64.6							(-)			
(-)			65.2							(-)			
(-)		(-)	15.9						98.0				
(-)		(-)											
(-)		(-)		(-)					8.7				
(-)		(-)		(-)									
(-)		(-)		(-)									
(-)				(-)									
(-)				(-)									
(-)		(-)	1000.0	<					12960.0				
(-)		(-)	6.3						188.0				
(-)				(-)									
(-)				(-)									
(-)		(-)	23.0										
(-)		(-)	24.2										
(-)			26.1						9.4				
(-)											0.0	4	
(-)		(-)											
(-)	2000.00	<				(-)	99.0				**7.1**	1	HBVDNA定量、IgM-HBc抗体
(-)		(-)	8.7								**2.5**	1	HBVDNA定量
(-)		(-)		(-)							**2.1**	3	HBVDNA定量
(-)		(-)	138.3			(-)	93.0				0.0	4	HBVDNA定量
(-)											0.0	4	HBVDNA定量
(-)													HBcrAg　3.0ﾐﾏﾝ
(-)		(-)	31.2			(-)	70.0		169.9		0.0	4	
(-)		(-)	43.2								0.0	4	
(-)		(-)	19.5			(-)	87.0		180.2				HBcrAg　<3.0
(-)		(-)	13.1			(-)	84.0		10.8				
(-)		(-)		(-)		(-)	82.0		10.9				
(-)		(-)	10.6			(-)	79.0		10.3				
(-)											0.0	4	
(-)				(-)					8.7				

(-)		(-)				(-)					0.0	4	HBcrAg　<3.0
(-)		(-)									0.0	4	
(-)		(-)		(-)					7.9				
(-)		(-)		(-)					8.7				
(-)		(-)		(-)					8.2				
(-)						(-)	90.0				0.0	4	
(-)			10.4										
(-)				(-)									
(-)				(-)									
(-)				(-)									
(-)			11.7						8.7				
(-)			21.1						7.0				

(-)		(-)		(-)					81.0				
(-)		(-)		(-)					3.4		**2.1**	1	
(-)		(-)		(-)		(-)		(-)	3.3		0.0	4	
(-)		(-)		(-)		(-)		(-)	2.6				HBcrAg　<3.0
(-)		(-)		(-)		(-)		(-)			0.0	4	
(-)		(-)		(-)		(-)		(-)			0.0	4	
(-)		(-)		(-)									
(-)	0.14												
(-)				(-)					1.1		**2.4**	1	
(-)		(-)	14.3			(-)		(-)	4.3		**1.0**	3	
(-)		(-)		(-)		(-)		(-)	7.1		0.0	4	
(-)						(-)	62.0				0.0	4	
(-)				(-)					8.2		0.0	4	
(-)		(-)											

(-)	2000.00	<		(-)					70.0		9.1	2	HBVDNA定量
(-)											7.3	1	HBVDNA定量
(-)											6.4	1	HBVDNA定量
(-)											5.9	1	HBVDNA定量
(-)											4.1	1	HBVDNA定量
(-)											3.1	1	HBVDNA定量
(-)	252.80			(-)		(-)	99.0		14600.0		2.7	1	HBVDNA定量
(-)											2.7	1	HBVDNA定量
(-)	48.50			(-)		(-)	98.0		13740.0		2.6	1	
(-)	32.40			(-)		(-)	98.0		100.0		2.2	1	
(-)											2.5	1	
(-)											2.1	1	
(-)	27.90		5.4			(-)	100.0		271.9		2.1	3	
(-)	13.60		6.5			(-)	100.0		283.4		2.1	3	
(-)	10.30		9.8			(-)	100.0		279.7		2.1	3	
(-)	14.70		7.1			(-)	100.0		283.2		2.1	3	
(-)	11.40												
(-)	11.60		7.0			(-)	100.0		273.9		2.1	3	HBcrAg　3.4
(-)	7.80		13.3			(-)	100.0		289.2		2.1	3	
(-)	6.80		10.9			(-)	100.0		271.7		0.0	4	
(-)	1.46			(-)		(-)	97.0		11.1		2.1	3	HBcrAg　3.4
(-)	1.20												
(-)	0.49			(-)		(-)	98.0		10.1		2.1	3	
(-)	0.17		48.3			(-)	99.0		11.6		2.1	3	
(-)	0.15		75.4			(-)	99.0		11.8		0.0	4	
(-)	0.19		76.4			(-)	99.0		10.8		0.0	4	
(-)	0.25		27.0			(-)	99.0		10.7		2.1	3	
(-)	0.31			(-)		(-)	96.0		11.8		2.1	3	
(-)											2.1	3	
(-)	0.17			(-)		(-)	99.0		10.8		2.1	3	
(-)	0.29												
(-)		(-)	77.3						11.9				
(-)													HBcrAg　<3.0
(-)		(-)	240.7			(-)	99.0		8.9		0.0	4	
(-)		(-)	126.5			(-)	99.0		9.9		0.0	4	
(-)		(-)	124.2						10.6		0.0	4	
(-)		(-)	98.6						10.3		0.0	4	HBcrAg　<3.0
(-)											0.0	4	
(-)											2.1	3	
(-)											2.1	3	
(-)		(-)	89.4						9.2				HBcrAg　<3.0
(-)		(-)									0.0	4	
(-)		(-)	119.7						9.5		0.0	4	
(-)		(-)									0.0	4	
(-)											0.0	4	

(-)		(-)		(-)					53.0				
(-)											0.0	4	HBVDNA定量
(-)		(-)											
(-)		(-)		(-)						(-)	0.0	4	
(-)		(-)		(-)						(-)			
(-)		(-)	81.1							(-)			
(-)		(-)		(-)									
(-)		(-)		(-)									

(-)				(-)									
(-)				(-)									
(-)		(-)	16.0						50.0				
(-)		(-)		(-)						(-)			
(-)		(-)		(-)						(-)			
(-)		(-)		(-)						(-)			
(-)		(-)		(-)						(-)			
(-)		(-)	241.0						52.0				
(-)		(-)											
(-)		(-)											
(-)		(-)	186.2										
(-)		(-)	157.8							(-)			
(-)			217.5							(-)			
(-)			217.1										
15.2		(-)	5.0						97.0				
15.2		(-)		(-)					52.2				
15.2		(-)		(-)					10.0				
15.2		(-)		(-)					9.7				
15.2		(-)		(-)					8.2				
15.2				(-)					8.7				
15.2				(-)					6.7				
(-)		(-)											
(-)		(-)		(-)						(-)			
(-)		(-)		(-)					1.2				
(-)		(-)		(-)		(-)		(-)		(-)	0.0	4	
(-)		(-)	46.7							(-)			
(-)		(-)		(-)						(-)			
(-)		(-)		(-)						(-)			
(-)		(-)		(-)						(-)			
(-)		(-)		(-)						(-)			
(-)													HBcrAg <3.0
(-)		(-)		(-)						(-)			
(-)		(-)											
(-)				(-)						(-)			
(-)				(-)						(-)			
(-)				(-)						(-)	0.0	4	
(-)		(-)		(-)					69.0				
(-)		(-)	171.6							(-)			
(-)		(-)	729.4						1.6				
(-)		(-)	766.7						2.2				
(-)			1000.0	<									
(-)		(-)											
(-)				(-)					53.0				
(-)		(-)											
(-)											0.0	4	
(-)		(-)		(-)						(-)			
(-)		(-)		(-)						(-)			
(-)				(-)						(-)			
(-)		(-)											
(-)				(-)						(-)			
(-)				(-)						(-)			
(-)		(-)	1000.0	<					63.0				
(-)		(-)	304.5							(-)			
(-)		(-)											
(-)		(-)	167.3							(-)			
(-)		(-)	212.1							(-)			
(-)		(-)	256.5							(-)			
(-)		(-)	253.7							(-)			
(-)		(-)	252.4							(-)			
(-)		(-)	213.9							(-)			
(-)		(-)	269.1							(-)			
(-)		(-)	192.9							(-)			
(-)		(-)	229.8							(-)			
(-)			213.8							(-)			
(-)			218.5							(-)			
(-)			196.2							(-)			
(-)			232.2							(-)			
(-)		(-)	26.8						88.0				
(-)		(-)	41.6						7.5				
(-)		(-)		(-)					4.9				
(-)		(-)		(-)					5.7				
(-)		(-)		(-)					5.5				
(-)				(-)									
(-)		(-)											
(-)				(-)		(-)		(-)	6.8				
(-)											**2.1**	3	
(-)		(-)											
(-)		(-)											
(-)		(-)											

(-)		(-)							8.2				
(-)		(-)	225.8							(-)			
(-)		(-)	115.8						1.0				
(-)		(-)	99.8						1.1				
(-)		(-)	100.8						1.1				
(-)		(-)	101.7						1.1				
(-)			101.7						1.0				
(-)		(-)	104.0										
(-)			107.3						1.0				
(-)			93.4							(-)			
(-)			87.5							(-)			
(-)	3.00			(-)					14.0				
(-)											**2.1**	3	
(-)	3.90										**2.1**	3	
(-)											**2.1**	3	
(-)		(-)											
(-)		(-)	14.1						11.0				
(-)		(-)	19.4						10.6		0.0	4	
(-)		(-)	23.1						7.1		0.0	4	
(-)		(-)									0.0	4	
(-)		(-)		(-)							0.0	4	
(-)		(-)		(-)							0.0	4	
(-)		(-)		(-)							0.0	4	
(-)		(-)		(-)					7.4				
(-)				(-)					6.9				
(-)				(-)					6.4				
(-)		(-)		(-)					1.2				
(-)											0.0	4	
(-)													HBcrAg　3.0ﾐﾏﾝ
(-)		(-)		(-)						(-)			
(-)		(-)											
(-)		(-)											
(-)		(-)											
(-)		(-)											
(-)		(-)											
(-)		(-)											
(-)		(-)											
(-)				(-)						(-)			
(-)				(-)						(-)			
(-)		(-)		(-)						(-)			
(-)		(-)		(-)						(-)			
(-)		(-)		(-)					2.8				
(-)		(-)		(-)						(-)			
(-)				(-)									
(-)		(-)	6.3						28.4				
(-)											0.0	4	
(-)		(-)		(-)					16.4		0.0	4	HBcrAg　3.0ﾐﾏﾝ
(-)											**2.1**	3	
(-)		(-)		(-)		(-)		(-)	25.6				HBcrAg　<3.0
(-)											0.0	4	
(-)											0.0	4	
(-)		(-)											
(-)		(-)		(-)					8.6				
(-)		(-)											
(-)		(-)											
(-)		(-)											
(-)		(-)											
(-)				(-)									
(-)		(-)		(-)						(-)			
(-)		(-)	10.8							(-)			
(-)			40.0							(-)			
(-)		(-)											
(-)		(-)		(-)					1.1				
(-)		(-)	35.1						1.0				
(-)		(-)								(-)			
(-)		(-)								(-)			
(-)			77.6										
(-)			79.5										
(-)			92.6										
(-)			102.5										
1.1		(-)	16.9						17.9				
1.1		(-)	11.6						10.1				
1.1		(-)		(-)					10.7				
1.1		(-)	10.7										
1.1		(-)											
		(-)		(-)					4.7				
											**2.3**	1	
		(-)		(-)		(-)	94.0		5.2		**2.4**	1	HBcrAg　<3.0
		(-)		(-)					8.5				

		(-)	66.4						7.1				
		(-)											
		(-)		(-)					6.2				
(-)		(-)		(-)					77.3				
(-)											0.0	4	
(-)				(-)					9.0				
(-)											0.0	4	
(-)		(-)		(-)					12.0				
(-)		(-)							7.9		0.0	4	
(-)		(-)							7.6		0.0	4	
(-)		(-)							7.1		0.0	4	
(-)				(-)					4.5				
(-)		(-)											
(-)		(-)	11.7						14.5				
(-)		(-)	20.3						10.1				
(-)		(-)	20.8						9.9				
(-)		(-)		(-)					8.8				
(-)			33.9										
(-)			17.4										
74.0	0.10			(-)						(-)			
74.0	0.09			(-)		(-)		(-)		(-)	0.0	4	HBcrAg　<3.0
74.0	0.07			(-)							0.0	4	
74.0	0.08			(-)		(-)		(-)		(-)	0.0	4	
74.0	0.07			(-)		(-)		(-)		(-)	0.0	4	
74.0	0.05			(-)									
74.0		(-)		(-)									
74.0		(-)		(-)									
74.0		(-)		(-)									
74.0			1000.0	<									
(-)		(-)		(-)						(-)			ここは未感染
(-)		(-)	82.0			(-)	87.0		8.5		**4.8**	1	
(-)		(-)		(-)		(-)	90.0		10.1		**2.1**	3	
(-)													HBcrAg　<3.0
(-)		(-)	19.5			(-)	88.0		10.5		0.0	4	
(-)											0.0	4	
(-)		(-)	47.9			(-)	90.0		11.0				
(-)		(-)	118.4			(-)	83.0		9.3		0.0	4	
(-)		(-)	88.3			(-)	85.0		9.4		0.0	4	
(-)		(-)	37.8						8.6				
(-)													HBcrAg　<3.0
(-)											0.0	4	
(-)		(-)		(-)									
(-)		(-)							8.7				
(-)		(-)							8.0				
(-)				(-)									
(-)		(-)	21.6						10.9				
(-)		(-)		(-)					10.3				
(-)		(-)											
(-)			44.2										
(-)			36.6										
(-)		(-)											
(-)			31.8										
(-)			39.2										
(-)		(-)	79.6						2.2				
(-)		(-)	48.6							(-)			
(-)			23.1							(-)			
(-)		(-)		(-)					7.3				
(-)											**2.1**	3	
(-)		(-)											
(-)				(-)					5.7				
(-)				(-)					5.3				
		(-)		(-)					9.0				
		(-)		(-)		(-)		(-)	9.0		0.0	4	
		(-)		(-)		(-)		(-)	8.7				
		(-)		(-)									
		(-)		(-)					6.2				
			40.2										
			65.5						4.9				
(-)		(-)		(-)					8.8				
(-)											0.0	4	
(-)											0.0	4	
(-)		(-)		(-)					7.4				
(-)		(-)											
(-)				(-)					7.0				
(-)				(-)					7.5		0.0	4	
(-)				(-)					7.0		0.0	4	
(-)				(-)					6.0				
(-)		(-)							5.2		0.0	4	

(-)		(-)												
(-)		(-)												
(-)		(-)	637.3						1.4					
(-)		(-)	1000.0	<					1.2					
(-)			1000.0	<						(-)				
(-)	250.00	<		(-)					8.5					
(-)	250.00	<		(-)	1340.1			(-)	8.2		9.1	2		
(-)	250.00	<		(-)	1567.5			(-)	8.3		7.2	1		
(-)	250.00	<		(-)	1482.2			(-)	8.7		6.0	1		
(-)	250.00	<		(-)	1583.1			(-)	8.7		5.5	1		
(-)	250.00	<		(-)	1523.3			(-)	9.4		4.3	1		
(-)	250.00	<		(-)	1373.4			(-)	9.7		3.8	1		
(-)	250.00	<		(-)	1236.6			(-)	9.5		3.3	1		
(-)	250.00	<		(-)	338.4			(-)	11.3		3.0	1		
(-)	169.17			(-)	86.5			(-)	8.9		2.6	1		
(-)	91.69			(-)	56.4			(-)	9.1		2.3	1		
(-)	67.55			(-)	81.3			(-)			2.4	1		
(-)	0.87			(-)		(-)		(-)			2.1	3		
(-)		(-)		(-)		(-)	92.0				2.1	3		
(-)		(-)		(-)		(-)	81.0				2.1	3		
(-)		(-)		(-)		(-)	65.0				2.1	3		
(-)		(-)		(-)		(-)		(-)			0.0	4		
(-)		(-)		(-)		(-)		(-)			1.8	3		
(-)		(-)		(-)		(-)		(-)			0.0	4		
(-)		(-)		(-)		(-)		(-)			0.0	4		
(-)		(-)		(-)		(-)	51.0						HBcrAg	3.9
(-)		(-)		(-)		(-)		(-)					HBcrAg	3.9
(-)		(-)		(-)		(-)		(-)					HBcrAg	3.8
(-)		(-)		(-)		(-)		(-)					HBcrAg	3.8
(-)		(-)		(-)		(-)		(-)						
(-)		(-)		(-)		(-)		(-)						
(-)		(-)		(-)		(-)		(-)						
(-)		(-)		(-)		(-)		(-)					HBcrAg	3.8
(-)		(-)		(-)		(-)		(-)					HBcrAg	3.7
(-)		(-)		(-)		(-)		(-)					HBcrAg	3.7
(-)		(-)		(-)		(-)		(-)					HBcrAg	3.9
(-)		(-)		(-)		(-)		(-)					HBcrAg	3.7
(-)		(-)		(-)		(-)		(-)						
(-)		(-)	19.1			(-)		(-)	9.2					
(-)		(-)		(-)					10.6					
2.6		(-)		(-)					9.8					
2.6											0.0	4		
2.6		(-)		(-)					10.9					
2.6		(-)	10.2						8.8					
(-)	0.05			(-)					9.6		0.0	4		
(-)	0.05			(-)		(-)	71.0		9.4		0.0	4		
(-)											0.0	4		
(-)											0.0	4		
(-)		(-)		(-)		(-)	72.0				0.0	4		
(-)		(-)		(-)		(-)	70.0							
(-)											0.0	4		
(-)											0.0	4		
(-)											0.0	4		
(-)		(-)		(-)		(-)	73.0							
(-)		(-)		(-)										
(-)		(-)		(-)		(-)		(-)		(-)				
(-)		(-)		(-)					7.0					
(-)		(-)												
(-)		(-)		(-)					7.1					
(-)		(-)		(-)					8.5					
(-)				(-)					8.4					
(-)				(-)					6.3					
(-)		(-)		(-)					8.9					
(-)						(-)	50.0				0.0	4		
(-)		(-)		(-)					7.9				HBcrAg	<3.0
(-)		(-)	14.7						6.4					
(-)		(-)	13.1						7.1					
(-)			15.6						7.7					
(-)		(-)	15.9						7.8					
(-)			16.7						7.9					
(-)				(-)					6.0					
(-)		(-)	76.6						1.3					
(-)		(-)	104.0						1.4					
(-)		(-)	114.3						1.1					
(-)			87.3						1.0					
(-)			92.4							(-)				
(-)			77.4							(-)				
(-)		(-)		(-)					10.1					
(-)				(-)										

(-)			12.8									
(-)				(-)								
(-)		(-)		(-)					4.6			
(-)											**2.2**	1
(-)						(-)		(-)				
(-)		(-)		(-)					8.6			
(-)			80.7									
(-)			204.3						5.5			
(-)		(-)		(-)					1.0			
(-)		(-)										
(-)		(-)										
(-)									1.2			
(-)											0.0	4
(-)				(-)								
(-)		(-)		(-)								
(-)		(-)		(-)								
(-)				(-)								
(-)				(-)								
(-)		(-)										
(-)				(-)					5.6			
(-)											0.0	4
(-)		(-)							4.5			
(-)		(-)		(-)								
(-)		(-)										
(-)				(-)					3.6			
(-)		(-)										
(-)				(-)								
(-)		(-)										
(-)				(-)					2.7			
(-)		(-)										
(-)				(-)					8.0			
(-)		(-)		(-)					6.4			
(-)				(-)								
(-)									5.5			
(-)		(-)										
(-)				(-)					8.2			
(-)											0.0	4
(-)		(-)										
(-)		(-)		(-)					7.9			
(-)											0.0	4
(-)				(-)					8.1			
(-)			48.6						7.9			
71.9		(-)										
71.9				(-)								
71.9				(-)					1.3			
71.9				(-)					1.1		0.0	4
71.9											0.0	4
71.9				(-)								
71.9											0.0	4
(-)		(-)										
(-)				(-)					8.8			
(-)				(-)								
(-)				(-)					6.8			
(-)	41.15											
(-)			12.6			(-)	75.0				**2.8**	1
(-)		(-)										
(-)				(-)					5.4			
(-)											**2.5**	1
(-)											0.0	4
(-)											0.0	4
(-)		(-)										
(-)				(-)					8.7			
(-)											**3.1**	1
(-)		(-)										
(-)		(-)		(-)							1.2	1
(-)		(-)	17.1						5.8		0.0	4
(-)		(-)	11.6						4.5		0.0	4
(-)		(-)		(-)					3.8		0.0	4
(-)		(-)		(-)					3.4		0.0	4
(-)		(-)		(-)					2.8		0.0	4
(-)		(-)		(-)					3.0		0.0	4
(-)		(-)		(-)					2.2		0.0	4
(-)		(-)										
(-)			23.7							(-)	**1.0**	3
(-)											0.0	4
(-)											0.0	4
